# Dumbbell‐Structured Plasmonic‐Enhanced Optical Nanoprobes Boosting Photo‐Magnetic‐Acoustic Multimodal Imaging‐Guided Photodynamic‐Photothermal Synergistic Treatment and Immunogenic Death in Nasopharyngeal Carcinoma

**DOI:** 10.1002/advs.75357

**Published:** 2026-04-16

**Authors:** Baikang Zhuang, Yubiao Yang, Wen Han, Yi Tang, Chengxin Yao, Fuli Zhao, Wenxiao Fang, Jinjie Li, Xiaolan Huo, Yiqian An, Yuanzhi Shao, Botao Gao, Jinchang Yin

**Affiliations:** ^1^ Guangdong Key Laboratory for Biomedical Measurements and Ultrasound Imaging National‐Regional Key Technology Engineering Laboratory for Medical Ultrasound School of Biomedical Engineering Shenzhen University Medical School Shenzhen University Shenzhen China; ^2^ State Key Laboratory of Optoelectronic Materials and Technologies School of Physics Sun Yat‐sen University Guangzhou China; ^3^ School of Systems Science and Engineering School of Science Sun Yat‐sen University Shenzhen China; ^4^ Institute of Biological and Medical Engineering Guangdong Academy of Sciences Guangzhou China

**Keywords:** gold nanorods, immunoregulation, multimodal imaging, NIR optical imaging, photothermal and photodynamic therapy, rare‐earth oxides, tumor‐targeted nanoprobes

## Abstract

Herein, we report a high‐performance optical nanoprobe featuring dumbbell‐shaped mesoporous silica‐coated gold nanorods, loaded with rare earth‐doped gadolinium oxide nanocrystals and photosensitizer indocyanine green, and functionalized with targeting peptides. The nanoprobe exhibits enhanced near‐infrared photoluminescence performance while enabling efficient photothermal conversion, photodynamic effect, photoacoustic imaging, and magnetic resonance imaging enhancement. Theoretical simulations demonstrate that the local surface plasmon resonance effect of gold nanorods exerts a significant enhancement on the optical performance of nanoprobes. Moreover, metal atoms play a pivotal role in modulating excited states and enhancing the intersystem crossing of indocyanine green. In vitro experiments confirm that the nanoprobe exhibits favorable biocompatibility and tumor‐targeting capacity; under 808 nm laser irradiation, it enables synergistic therapy by generating reactive oxygen species and inducing hyperthermia, with the mechanism underlying synergistic treatment elucidated from both physical and biological perspectives. The nanoprobe facilitates near‐infrared photo‐magnetic‐acoustic‐thermal multimodal imaging, achieves efficient photothermal‐photodynamic synergistic therapy on tumor‐xenografted mice upon the laser irradiation, exerts effective inhibition on tumor growth, and mitigates tumor recurrence by inducing immunogenic cell death. This study offers insights into the design of novel high‐performance optical nanoprobes and highlights the application potential of this nanoprobe in the precise diagnosis and synergistic treatment of nasopharyngeal carcinoma.

## Graphical abstract

This study reports a novel rationally‐designed optical nanoprobe based on dumbbell‐shaped mesoporous silica‐coated gold nanorods, loaded with rare‐earth oxides, photosensitizers, and tumor‐targeted peptides, enabling plasmonic‐enhanced multimodal imaging and PTT‐PDT synergy. Integrated simulations and theoretical calculations reveal the in‐depth enhancement mechanism. The nanoprobe presents high‐sensitivity tumor imaging, exerts effective inhibition on tumor growth and activates immune responses via immunogenic cell death, highlighting great potential in precise theranostics of nasopharyngeal carcinoma.

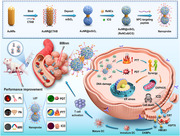

## 1 Introduction

Nasopharyngeal carcinoma (NPC) is a malignant tumor originating from the mucosal epithelium of the nasopharynx [1, 2]. However, conventional treatments including radiotherapy, chemotherapy, and surgery face severe NPC‐specific clinical challenges, such as deep nasopharynx location, limited resection, radiotherapy‐related mucosal damage, high metastasis, and severe off‐target effects [2, 3]. These unmet clinical needs urgently require highly effective, low‐toxicity, targeted, and minimally invasive therapeutic strategies for NPC. Benefiting from the deep tissue penetration and minimal photodamage of near‐infrared (NIR) light, NIR phototheranostic technology [4, 5, 6, 7] composed of optical imaging and phototherapy has emerged as a promising minimally invasive modality for NPC. Upon NIR light irradiation, probes enriched in NPC lesions via active targeting can efficiently convert photon energy into local hyperthermia and reactive oxygen species (ROS), thus sufficiently triggering photothermal therapy (PTT) and photodynamic therapy (PDT) effects against tumor cells with minimal damage to surrounding normal tissues [8, 9, 10, 11]. Developing targeted multimodal probes for NPC, applicable in molecular imaging and combined PTT‐PDT therapy, enriches current treatment methods and also significantly enhances therapeutic efficacy, offering substantial clinical potential [9, 10, 11, 12, 13, 14]. Nevertheless, most reported NIR phototheranostic nanoprobes still suffer from two critical bottlenecks. Insufficient photoluminescence, photothermal conversion and ROS generation efficiency result in limited imaging sensitivity and therapeutic efficacy at safe laser doses. In addition, the mechanisms underlying optical performance enhancement and the synergistic interaction between PTT and PDT in tumor cells remain poorly elucidated, which severely hinders the rational design of high‐performance nanoprobes and their clinical translation [14, 15]. Therefore, it is urgent to develop NPC‐targeted nanoprobes with boosted optical performance and systematically elucidate the physical and biological mechanisms underlying performance enhancement and PTT–PDT synergistic therapy. These are essential prerequisites for achieving efficient and safe NPC phototheranostics.

To address the above bottlenecks, rational design of plasmonic nanoprobes based on gold nanostructures has become a research hotspot in biophotonics [16, 17, 18]. The unique localized surface plasmon resonance (LSPR) effect of gold nanorods can generate strong localized electric field (LEF) enhancement around nanoparticles, which can effectively boost the optical conversion efficiency of adjacent fluorophores and photosensitizers, providing a physical basis for the simultaneous enhancement of multimodal imaging performance and phototherapeutic efficacy [19, 20, 21]. Meanwhile, by optimizing the component and structure of plasmonic nanoprobes, multifunctional modules can be integrated into a single nanoparticle, which is conducive to realizing the synergistic effect of PTT and PDT, thus greatly improving antitumor efficacy under low laser doses [22, 23]. Previous studies have realized the regulation of LSPR effects in gold nanorods (AuNRs) through structural design, and achieved enhanced optical and photoacoustic properties [16, 17, 18, 24]. For instance, Jokerst et al. achieved synergistic performance enhancement by the controlled assembly and dissociation of citrate‐capped gold nanorod [24]. However, most of these studies mainly focused on the regulation of single‐modal optical performance, few of them have realized the simultaneous enhancement of NIR‐II photoluminescence, photothermal conversion, ROS generation, and optoacoustic contrast in a single NPC‐targeted nanoprobe. More importantly, the physical mechanism of LEF‐mediated optical enhancement and the biological mechanism of PTT‐PDT synergistic tumor killing have rarely been systematically elucidated from both physical and biological perspectives, which is the major innovative feature of this study.

**FIGURE 1:**
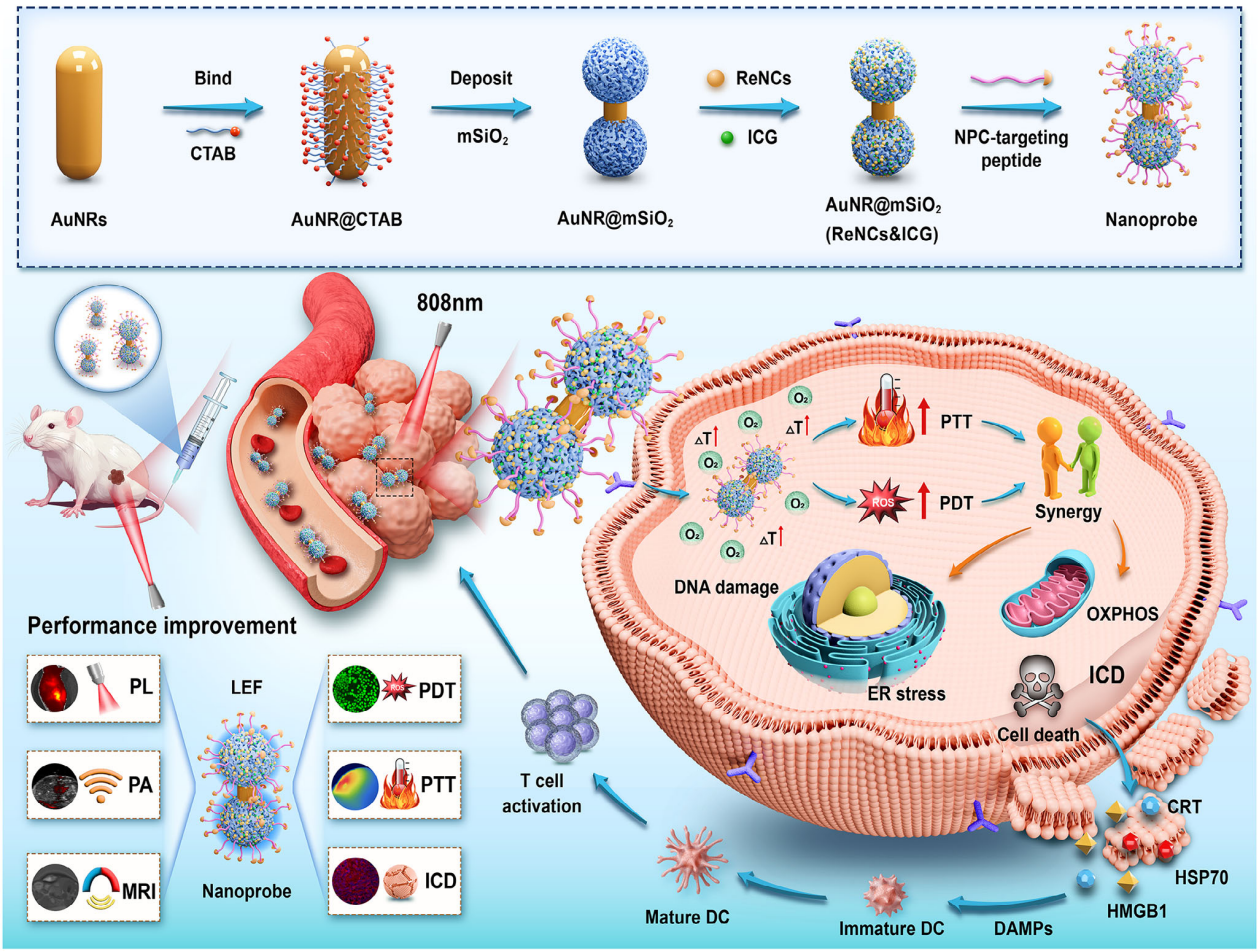
Schematic illustration of the preparation and performance improvements of dumbbell‐shaped nanoprobes for multimodal imaging and tumor phototheranostics.

In this study, we rationally designed and fabricated an NPC‐targeted multifunctional plasmonic composite nanoprobe with a dumbbell‐like structure, which simultaneously realized LEF‐boosted multimodal imaging and highly efficient PTT‐PDT synergistic therapy for NPC. Specifically, mesoporous silica (mSiO_2_)‐coated AuNRs were used as the plasmonic core to generate strong LEF enhancement; rare‐earth‐doped gadolinium oxide nanocrystals (ReNCs) and the photosensitizer indocyanine green (ICG) were incorporated onto the silica shell and within its mesoporous channels, while nasopharyngeal carcinoma (NPC)‐targeting peptides were covalently attached to the surface of the nanoprobe via silane coupling to achieve active tumor targeting (Figure 1). The prepared nanoprobes exhibited excellent NIR II photoluminescence (PL), efficient photothermal and photoacoustic conversion, enhanced photodynamic properties, and superior magnetic resonance imaging (MRI) contrast, rendering them highly promising for high‐sensitivity multimodal imaging and efficient phototheranostics. Finite‐difference time‐domain (FDTD) algorithm confirmed that the strong LEF generated by AuNRs under NIR irradiation significantly amplified the PL of ReNCs and enhanced the photodynamic performance of ICG, revealing the physical mechanism of optical performance enhancement. In vitro cellular experiments confirmed the excellent biocompatibility, NPC‐targeted uptake, and significant PTT‐PDT synergistic cytotoxicity of the nanoprobes. Notably, we systematically explored the molecular mechanism of PTT‐PDT synergistic tumor killing through mathematical modeling and proteomics analysis, revealing the PTT‐PDT interaction mechanism of the synergistic therapy from both physical and biological perspectives. Furthermore, studies in NPC xenograft mouse models demonstrated that the nanoprobe enabled precise NPC‐targeted NIR photo‐acoustic‐magnetic‐thermal multimodal imaging and achieved remarkable antitumor therapeutic efficacy, completely eradicating the tumors while preventing recurrence and metastasis through the induction of immunogenic cell death (ICD). This study not only provides a high‐performance theranostic nanoprobe for NPC, but also offers new insights into the rational design of plasmonic‐enhanced multimodal phototheranostic platforms, which holds important guiding implications for the clinical translation of biophotonic tumor therapy.

## 2 Results and Discussion

### 2.1 Assembly and Characterization of Nanoprobes

**FIGURE 2:**
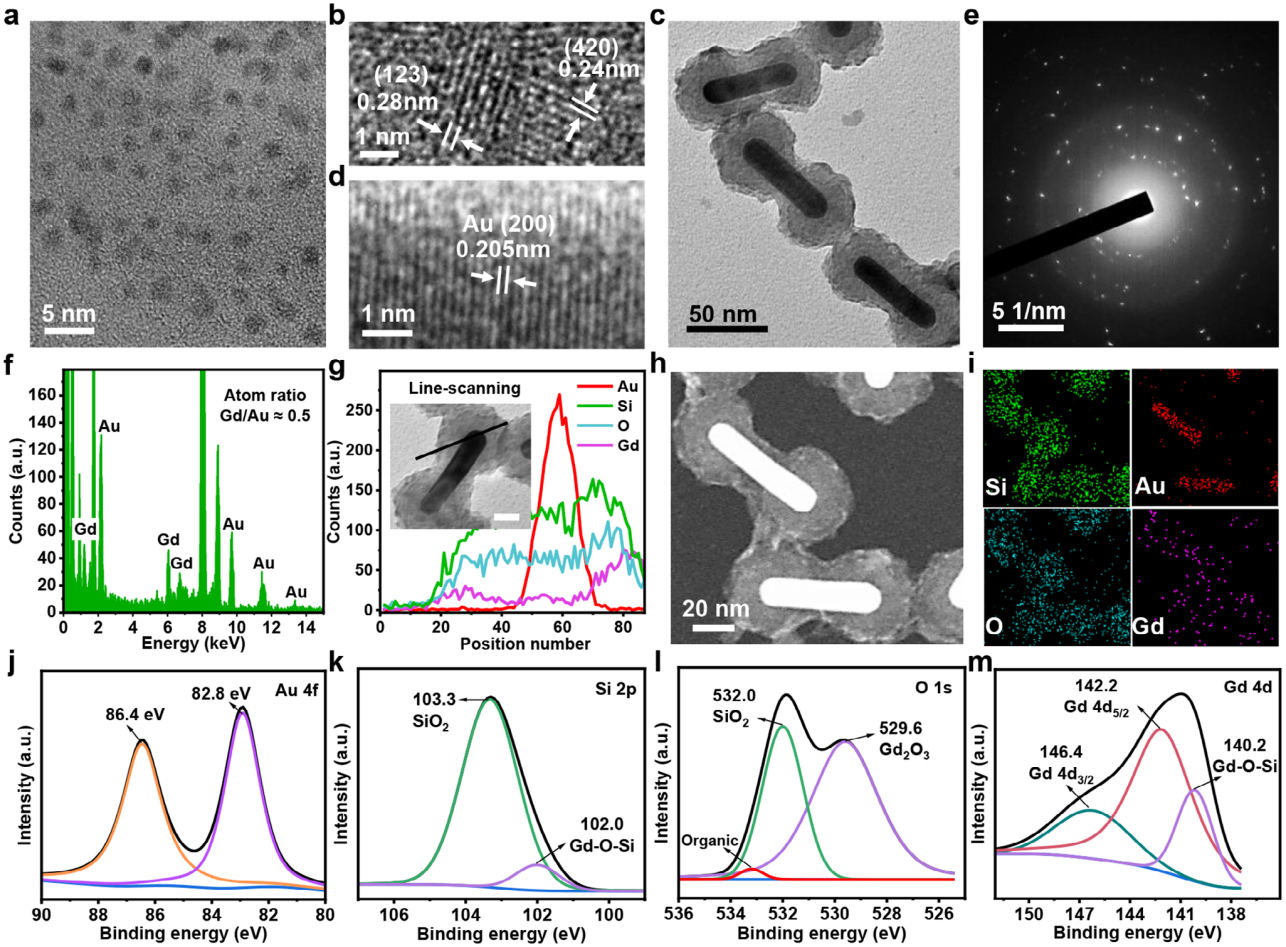
Characterization of nanoprobes. (a) Typical TEM and (b) HR‐TEM images of ultrafine ReNCs; (c) TEM and (d) HR‐TEM images of the dumbbell‐shaped composite nanoprobes; (e) Selected area electron diffraction pattern; (f) EDX spectrum of the nanoprobe, indicating that the atomic ratio of rare‐earth to Au is approximately 0.5; (g) EDX line‐scanning profiles analyzed across the end region as viewed along the line in the inset of TEM image; (h) High‐angle annular dark‐field scanning TEM (HAADF‐STEM) image of the used nanoprobe for the corresponding (i) EDX elemental mapping images of Si, O, Au, and Gd elements. (j‐m) HR‐XPS spectrum of Au 4f, Si 2p, O 1s, and Gd 4d for the composite nanoprobe.

Figure 2a illustrates the successful preparation of ReNCs via high‐boiling polyol dehydration, resulting in particles approximately 3 nm in size with excellent dispersion and uniformity. The high‐resolution transmission electron microscopy (HR‐TEM) image of a single nanoparticle (Figure 2b) reveals distinct lattice fringes, indicating the excellent crystallinity of the particles. The 2D lattice of ReNCs mainly corresponds to the 420 and 123 crystal plane indices, confirming their cubic crystal structure according to HR‐TEM and Fourier transform analysis of a single rare‐earth particle (Figure S1). AuNRs were synthesized via a seed growth method, followed by coating of mSiO_2_ at both ends of the nanorods using sol–gel process with controlled surfactant concentration. After refluxing in saline‐ethanol solution to clean the mesoporous channels and activate the surface binding sites, ultrafine ReNCs were anchored within the mesopores via ultrasonic treatment. The mSiO_2_ was then aminated, followed by sequential loading of ICG and NPC‐targeting peptides (Figures S2–S4). Quantum mechanical calculations confirm that gadolinium oxide forms stable bonds with silica (Figures S5 and S6 and Tables S2–S5), while ICG molecules, in addition to being aminated to silica, also interact with the silica matrix, achieving a stable composite nanoprobe system (Figures S7–S10). The prepared composite nanoprobe shows a dumbbell‐shaped morphology (Figure 2c). Due to the stepwise loading of ReNCs, ICG, and NPC‐targeting peptides into mSiO_2_, the mesoporous channels of the dumbbell structure are not clearly visible (Figures S11 and S12). Figure 2d,e reveals clear lattice fringes of AuNRs, whereas the diffraction spots of ReNCs are faint owing to their small size.

Energy dispersive X‐ray spectroscopy (EDX) demonstrates that the atomic ratio of rare‐earth elements to gold is approximately 1:2, indicating an excellent loading efficiency of ReNCs in the nanoprobe (Figure 2f). Line scanning and elemental mapping present that rare‐earth elements are primarily loaded on both pore channels and surface of the mesoporous dumbbell (Figure 2g‐i; Figure S12). High‐resolution X‐ray photoelectron spectroscopy (XPS) spectra provide further insights into the electronic states of the elements. Due to the dumbbell structure of the nanoprobe, gold element near the surface produces detectable signal peaks (Figure 2j). The XPS spectra of silicon, oxygen, and gadolinium confirm the bonding state between silica and gadolinium oxide (Figure 2k‐m), demonstrating that the dumbbell‐structured composite nanoprobe exhibits excellent structural stability.

### 2.2 Optical Performance Enhancement of Nanoprobes

**FIGURE 3:**
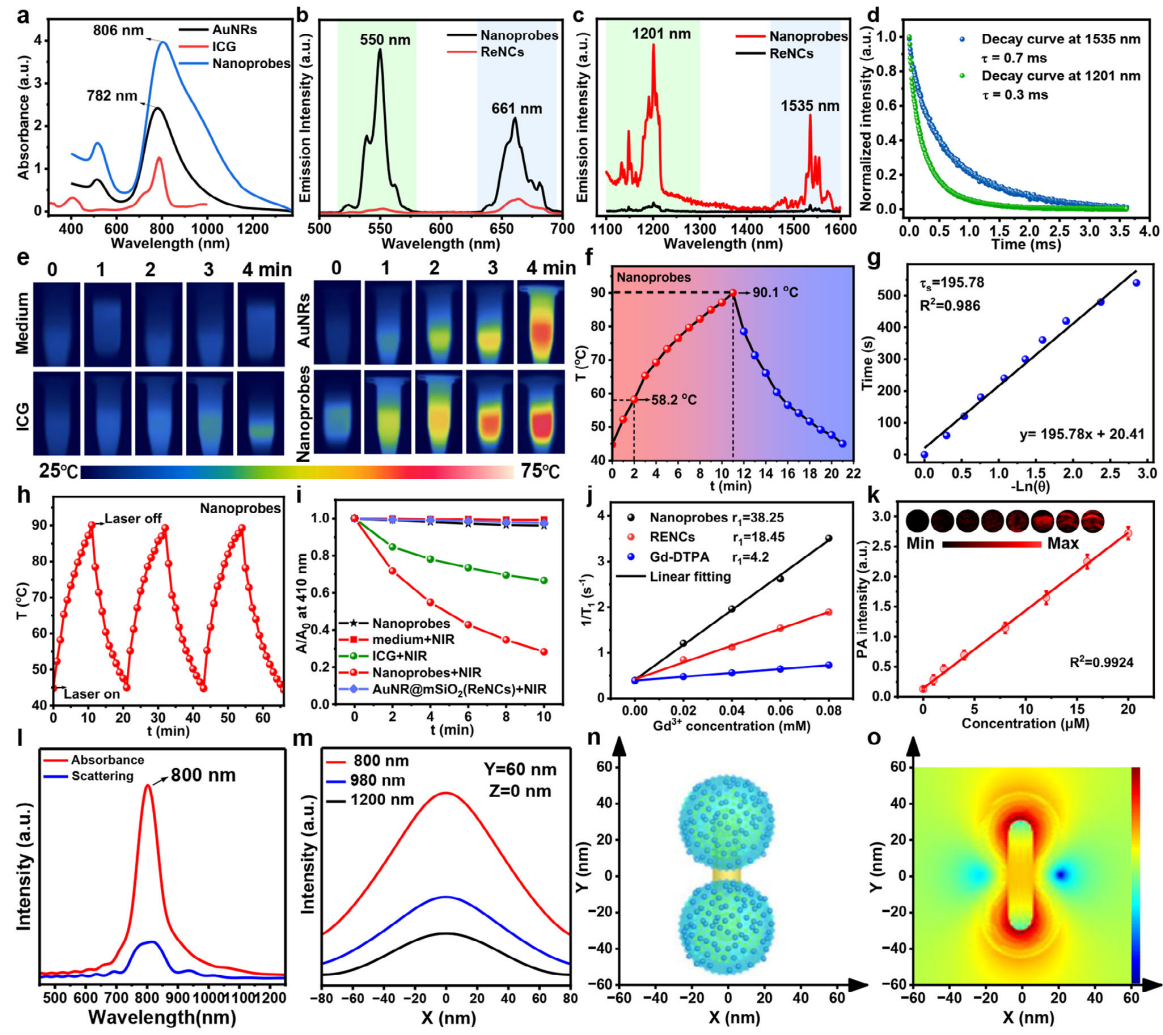
Performance enhancement of nanoprobes. (a) Absorption spectra of the nanoprobes, AuNRs and ICG solutions; (b) Upconversion PL emission and (c) Near‐infrared second‐window (NIR‐II) PL emission spectra of nanoprobes and ReNCs under 980 nm laser excitation; (d) Fluorescence decay curves of nanoprobes for the emission peaks at 1201 and 1535 nm; (e) Thermal images of the medium, ICG, AuNRs, and nanoprobes solution under 808 nm laser irradiation (0.5 W/cm^2^); (f) Time‐dependent temperature curve; (g) Time constant of heat transfer measured using linear regression of the cooling profile; (h) Temperature variation of the nanoprobe solution during three photothermal heating cycles under 808 nm laser irradiation; (i) Time‐dependent change in relative absorption intensity of the active oxygen indicator DPBF in the nanoprobe solution; (j) MR T_1_ relaxivity profiles of nanoprobes compared with pure ultrafine ReNCs and clinical Gd‐DTPA; (k) Photoacoustic signal intensities as a function of nanoprobe concentrations. The insert images represent corresponding imaging sections acquired at increasing concentrations. Data are presented as mean ± SD; (l) NIR absorption cross section simulated with FDTD algorithm. (m) Electric field enhancement curve along the X axis through different incident excitations. (n) Simulated structure models of the nanoprobe, and (o) the corresponding LEF distribution.

Figure 3a presents the absorption spectrum of the composite nanoprobes. After integrating AuNRs, ICG, and ReNCs into mSiO_2_ nanoparticles, the optimal absorption peak of the nanoprobe is tuned to 806 nm, which aligns with the emission wavelength of the 808 nm laser. Due to the strong absorption of nanoprobes in the visible range, ReNCs, when excited at 980 nm, show a decrease in the upconversion emission peaks of holmium (Ho^3+^) and erbium (Er^3+^) ions at 550 and 661 nm, respectively. However, red emission at 661 nm persists to a certain extent (Figure 3b). In contrast to upconversion luminescence, followed integrating with AuNRs and ICG, ReNCs present enhanced emissions of Ho^3+^ and Er^3+^ ions at 1201 and 1535 nm (Figure 3c). This enhancement arises from the LEF produced by AuNRs at the 980 nm absorption, which significantly amplifies the emissions of ReNCs in NIR region. The emission peaks at 1201 and 1535 nm display extended luminescence lifetimes of 0.3 and 0.7 ms, respectively, reflecting the excellent potential for biological optical imaging of nanoprobes.

The composite nanoprobes exhibit remarkable photothermal conversion efficiency in aqueous solutions, with temperatures exceeding 58°C after just 2 min of irradiation with the 808 nm laser (Figure 3e,f; Figures S13 and S14). Based on the photothermal cooling rate, the photothermal conversion efficiency of nanoprobes is calculated to be 45.2% (Figure 3g). The photothermal cycling curve further demonstrates the stability of photothermal performance of nanoprobes (Figure 3h). When mixed with ROS indicators, the intensity of ROS production under 808 nm laser irradiation is indicated by a significant reduction in the absorption peak of indicator, confirming that the nanoprobes exhibit enhanced photodynamic performance compared to ICG alone (Figure 3i). ReNCs are renowned for their exceptional luminescent and electromagnetic properties, which contribute to enhanced magnetic resonance imaging. The longitudinal relaxivity of the isolated ReNCs was measured, revealing a nuclear magnetic relaxation performance significantly superior to that of the commercial contrast agent Gd‐DTPA, owing to the ultrahigh surface area of ultrasmall ReNCs. Upon loading ReNCs onto mSiO_2_, the relaxivity is further enhanced due to the spatial confinement effects [25]. of the mesoporous structure (Figure 3j). Furthermore, the nanoprobes can generate photoacoustic signals through laser‐induced thermal expansion. As the solution concentration increases, the photoacoustic signals progressively appear, and the signal intensity of nanoprobes is significantly stronger than that of pure AuNRs or ICG molecules, offering distinct advantages for tumor photoacoustic‐enhanced imaging (Figure 3k).

To validate the LSPR effect of AuNRs on ICG‐mediated PDT and rare‐earth‐based NIR PL, we employed the FDTD algorithm to calculate the LEF distribution around the nanoprobe under a range of structural parameters (Figure 3l; Figure S15 and Table S6). The simulated absorption cross‐section of the nanoprobe shows peak positions between 790 and 815 nm, consistent with the experimentally measured longitudinal absorption peak (Figure 3a). The absorption exhibits distinct electron‐hole transfer in light of quantum mechanical calculations (Figure S16), indicating the LSPR effect calculated via FDTD algorithm [26, 27]. When the incident excitation wavelength is set to 800 nm, with the Y and Z positions fixed at 60 and 0 nm outside the composite nanoprobe surface, the LEF along the X‐axis is significantly enhanced compared to the electric field at an incident excitation wavelength of 1200 nm (Figure 3m). Meanwhile, the excitation wavelength of 980 nm also results in a substantial LEF enhancement, which is a crucial factor for enhancing PL of ReNCs. The enhanced LEF is primarily concentrated at both ends of the gold nanorod, consistent with the dumbbell‐shaped structure of the nanoprobe (Figure 3n,o; Figures S17–S20). ICG is distributed in the channel pores and on the surface of mSiO_2_, with ReNCs larger than 3 nm mostly adhering to the silica surface, while those smaller than 3 nm are anchored in the mSiO_2_ channel pores at both ends of the AuNRs. Given that both ICG and ReNCs are located in the regions of maximum LEF enhancement, they can fully exploit the amplified LEF to enhance the photodynamic effect of ICG and NIR PL of ReNCs. The 808 nm laser used matches the LSPR peak of the nanoprobe and the absorption peak of ICG, generating the resonance effect that enhances the photodynamic effect. Moreover, the nanoprobe maintains high absorption at 980 nm (Figure 3a), which corresponds to the absorption peak of Yb^3+^ ions in ReNCs. The LSPR effect leads to a more pronounced charge separation, enhancing the excitation efficiency of Yb^3+^ ions at the dumbbell ends and then improving the NIR‐II PL efficiency.

### 2.3 Photodynamic Enhancement Mechanism and Excited‐State Characteristics

**FIGURE 4:**
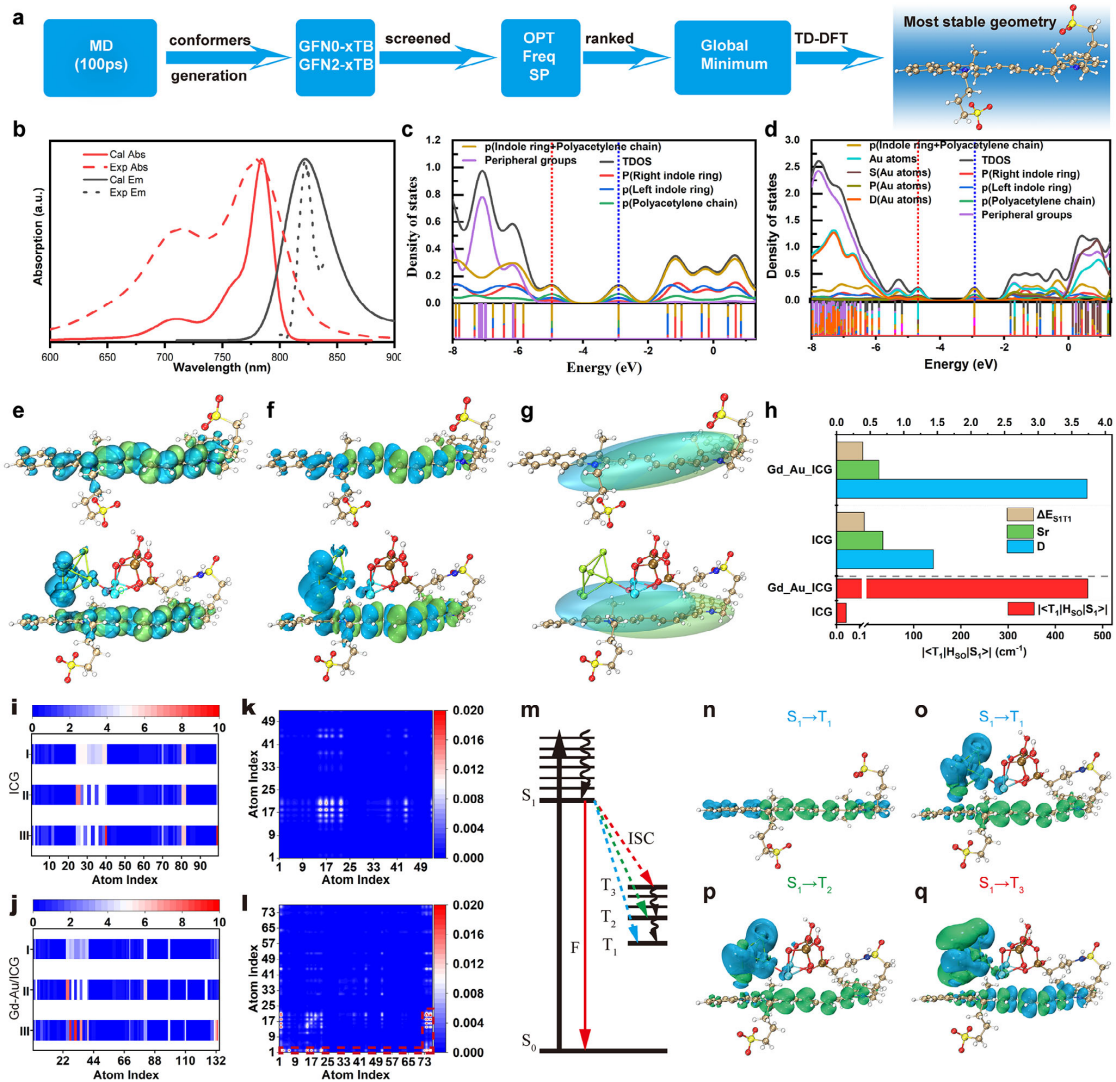
Electronic structure and electron excitation analysis. (a) Conformational searching procedure for ICG molecule. (b) The simulated (solid lines) and experimental (dashed lines and dotted lines) absorption and emission spectra for ICG in water. (c, d) DOS graphs of ICG and Gd_Au/ICG (simplified AuNR@SiO_2_(Gd_2_O_3_/ICG) model). Vertical dash lines in red and blue mark out positions of HOMOs and LUMOs energy level. Distances between vertical red lines and vertical blue lines represent the HOMO‐LUMO energy gaps. Insets on vertical dash lines in red and blue show isosurface maps of HOMOs and LUMOs. (e) The distributions of hole (blue) and electron (green), (f) charge density differences (CDD), and (g) smoothed distributions of hole (cyan) and electron (green) for ICG and Gd_Au/ICG. Green and blue regions in CDD maps denote the increase and decrease of electron density, respectively. (h) The calculated energy gaps (ΔE_S1T1_) and spin‐orbit coupling matrix elements (<T_1_|H_SO_|S_1_>) as well as D and Sr index values. D index represents the centroid distance of hole and electron distribution. Sr index denotes the overlap content of hole and electron distribution. (i) The electron‐hole atom heat maps for ICG and (j) Gd_Au/ICG exhibit the contribution of labelled non‐hydrogen atoms to the distributions of hole (I), electron (II) and their overlap (III). (k) Graphical representation of TDM for ICG and (l) Gd_Au/ICG at the S_1_ state. The red rectangles denote the main hole‐electron localization for ICG and Gd_Au/ICG. (m) The energy level diagrams and the deactivation pathways for ICG and Gd_Au/ICG. (n) The isosurfaces of charge density difference between singlet states and triplet states plotted on the optimized structures of ICG and (o‐q) Gd_Au/ICG. The blue and green isosurfaces indicate the decrease and increase in electron density during intersystem crossing.

To gain a better understanding of how the nanoprobes functioned as the highly efficient PDT agents, quantum mechanical calculations were employed to investigate the electronic excitation behaviors of the S_1_ states and the intersystem crossing (ISC) between these S_1_ states and T_n_ of ICG in the presence of AuNR@mSiO_2_(Gd_2_O_3_). After conformational search for ICG molecule (Figure 4a), the primary conformation with the nearest global energy minima of ICG molecule in water was determined (Figure S21a), which can be validated by the calculated absorption and emission spectra using TD‐DFT methods. The calculated maximum absorption and emission wavelengths are 800 and 812 nm for ICG (Figure 4b), aligning well with our experimental results (780 and 822 nm), signifying that the chosen conformations and DFT/TD‐DFT methods are appropriate for our study.

Based on the optimized structure of ICG, simplified model for our nanoprobe (i.e., Gd_Au/ICG) was constructed using sulfa linkers in accordance with the prepared nanocomposites (Figure S21b). Further calculation reveals that the absorption peak for ICG is mainly contributed from the highest occupied molecular orbital (HOMO) to the lowest unoccupied molecular orbital (LUMO) (over 99%), while HOMO‐1→LUMO transition contributes over 97% of the absorption peak for Gd_Au/ICG. Both HOMO and LUMO for ICG have nodal planes on the polyacetylene chain and indole ring (Figure S22), demonstrating the characteristic of π MOs in conjugated organic systems. Notably, part of HOMO‐1 for Gd_Au/ICG is located on Au and the LUMO is similar to that for ICG, which implies the participation of Au in the process of electronic excitation, which can be confirmed by partial density of states (Figure 4c,d). The HOMO and the LUMO for ICG primarily consist of p orbitals, specifically those in the polyacetylene chain. Au makes nearly equal contribution to LUMO‐1 for Gd_Au/ICG in contrast to polyacetylene chain and peripheral group. Moreover, contribution of angular momentum S of Au is more than P and D. It is concluded that the absorption peak for ICG is attributed to π→π* excitation, while the presence of Au induces additional n→π* excitation.

The hole (blue) and electron (green) distributions in Figure 4e validate the character of π→π* transition for ICG and π→π* as well as n→π* for Gd_Au/ICG in accordance with the above MO and DOS analysis. The charge density difference (CDD) map (Figure 4f) and Sr distribution (Figure S23) of ICG alone reveal that the increased charge density (green) and the decreased charge density (blue) locate on the polyacetylene chain and indole ring, alternately. Furthermore, combined the Sr index value of over 69% and D index value of less than 1.4 angstrom confirm primary nature of localized excitation for ICG (Figure 4h). In comparison, increased D index (3.73 Å), reduced Sr value (63%), and obviously separated smoothed distribution (Figure 4g) of hole and electron together confirm the presence of charge transfer excitation due to the metal Au.

In addition, the transition density matrix (TDM) contributed by specific atoms were also calculated to analyze the hole‐electron localization and the probability for the S_0_→S_1_ transition (Figure 4i‐l). The detailed label order for non‐hydrogen atoms can be found in Figure S24. Figure 4i,k indicates that the excitation distribution of the ICG is relatively localized within the atoms in polyacetylene chain and show a typical localized excitation nature. In comparison, the introduction of metals Au and Gd leads to increasingly delocalized excitations on not only polyacetylene chain but also indole ring of ICG and further induces the transfer from Au atoms to polyacetylene chain, as denoted by the red squares in Figure 4k. This observation enables a better understanding of the electron donor role that Au plays in the electronic excitation process of Gd_Au/ICG.

Photodynamic performance is directly related to ISC process between S_1_ and T_n_ [28], directly proportional to square of spin‐orbit coupling matrix element (SOCME, <T_n_|H_SO_|S_1_>), but inversely proportional to energy gaps between S_1_ and T_n_ (ΔE_S1Tn_) (Figure 4h; Table S7). Compared to ICG, the energy gap ΔE_S1T1_ of Gd_Au/ICG falls from 0.94 to 0.84 eV, while the SOCME experiences a substantial increase from 0.042 to 468.616 cm^−1^, representing a remarkable 11157% increment. Besides, S_1_ state can also cross to T_2_ and T_3_ state evaluated by still large SOCME values (over 63 cm^−1^) and small ΔE_S1Tn_, but ICG alone loses these two pathways due to the elevated triplet state energy and extremely low SOCME values (See Table S7). It can be concluded that the atoms, Gd and Au, largely facilitate the ISC promotion by the enhanced SOC heavy metal effects [29]. Corresponding photophysical relaxation pathways for the photodynamic effects can be presented as Figure 4m. To gain a deeper understanding of the ISC process, we computed the charge density difference distribution graphs between S_1_ and T_n_ in Figure 4n‐q. Given that there exists the transition from S_1_→T_1_ for ICG, the electron density for ICG increases in the polyacetylene chain and decreases in the two indole rings, requiring the occurrence of intramolecular charge transfer, which is not in accordance with our electron excitation analysis, thus implying the very small probability of occurrence. When the transitions from S_1_→T_1_ and S_1_→T_2_ for Gd_Au/ICG happen, electron density in both polyacetylene chain and indole rings will be increased, and electron density in the fragment of Au atoms will be mainly reduced, indicating the happen of strong charge transfer from Au atom to ICG, which could be easily satisfied according to the previous hole‐electron analysis. Similarly, Figure 4q needs localized excitation of both ICG itself and Au itself, which the former condition could be satisfied but the latter need very high excitation energy, thus cannot be met easily according to the DOS distribution (Figure 4d). The two more effective ISC pathways bring by Gd and Au make our nanoprobe more potential candidate as an excellent therapeutic reagent.

### 2.4 Cellular Uptake and Synergistic Therapeutics Upon NPC Cells

**FIGURE 5:**
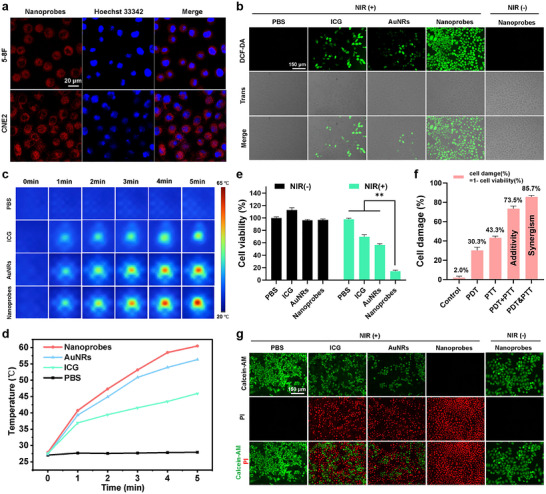
Cellular uptake and synergistic therapeutics of nanoprobes in vitro. (a) Confocal laser scanning microscopy (CLSM) images of 5–8F and CNE2 cancer cells upon incubation with nanoprobes for 4 h. (scale bar: 20 µm); (b) Fluorescence images of 5–8F and CNE2 cells stained with DCFH‐DA after different treatments under 808 nm laser irradiation (0.5 W cm^−2^, 5 min); (c) Infrared thermal images and (d) time‐dependent temperature curve of 5–8F cells incubated with nanoprobes and irradiated with 808 nm laser (0.5 W cm^−2^); (e) Cell viability of 5–8F cells after different treatments; (f) Cancer cell damage evaluation via cell counting kit‐8 (CCK‐8) assays under the control group (PBS, laser), ICG‐dominant PDT, AuNR‐mediated PTT, additive damage of PDT plus PTT (PDT + PTT) measured separately and nanoprobe‐mediated synergistic damage of PDT and PTT (PDD&PTD) measured simultaneously; (g) Live/dead fluorescence images of laser (0.5 W cm^−2^, 5 min) and nanoprobes treated 5–8F cells stained with calcein‐AM (green) and PI (red). Mean ± SD, n = 3, *p < 0.05, **p < 0.01, ***p < 0.001, ns: not significant.

The nanoprobe exhibits remarkable PL in the NIR region. Due to the broad NIR emission spectrum of the ICG molecules in the nanoprobe, signals can be detected in the red fluorescence channel during cellular imaging. Figure 5a demonstrates that the nanoprobe shows outstanding fluorescence in both the high‐metastasis NPC cell line 5–8F cells and the poorly differentiated NPC cell line CNE2 cells, primarily accumulating in the cytoplasm. Fluorescence images overlap with bright‐field microscopy images, with no noticeable morphological changes or vesicle formation indicative of apoptosis, suggesting that the nanoprobe is effectively internalized by cells through the action of the NPC‐targeting peptide and does not exhibit apparent cytotoxicity (Figures S25–S28). Following a 2 h co‐incubation of the nanoprobe with NPC cells, ROS production was measured using DCFH dye (Figure 5b; Figures S29 and S30). Hydrogen peroxide (H_2_O_2_)‐treated cells were set as the positive control to validate the reliability of ROS detection. Upon 808 nm laser irradiation (0.5 W/cm^2^), no ROS signal was detected in control cells, whereas cells co‐incubated with the nanoprobe produced a significant ROS signal after 5 min of irradiation, confirming the robust PDT potential of nanoprobes. ROS production was also observed in other NPC cell types (Figure S31).

Additionally, infrared thermal imaging was used to monitor the cell temperature under laser exposure. Compared to the control group, the nanoprobes induce a higher temperature rise, with cell temperatures exceeding 60°C after 5 min of irradiation (Figure 5c,d). Under the integrated photothermal and photodynamic effects of nanoprobes, CCK‐8 assays (Figure 5e,f) reveal a cell mortality rate of 85.7%, significantly higher than the addictive of mortality rates observed with AuNRs‐mediated PTT and ICG‐dominant PDT, demonstrating a pronounced synergistic effect under low‐power NIR laser irradiation. Following the 2 h co‐incubation with nanoprobes and 808 nm laser irradiation (0.5 W/cm^2^), live/dead cell staining was performed using Calcein‐AM and propidium iodide (PI) to evaluate cell viability and cytotoxicity (Figure 5g; Figure S32). After 808 nm laser irradiation, CNE2 cells displayed green fluorescence, indicating live cells. In contrast, cells co‐incubated with the nanoprobe and irradiated for 5 min turned red, signifying nearly complete cell death. The quantitative results showed that the nanoprobe‐mediated photothermal‐photodynamic synergistic treatment induced a significantly higher dead cell ratio (up to 86%) compared to individual PDT or PTT treatments, which was consistent with the CCK‐8 assay results and indicated the potent cellular killing ability of the nanoprobes upon NIR laser irradiation (Figure S33). This further confirms the efficacy of nanoprobe in inactivating NPC cells and highlights its excellent synergistic photothermal‐photodynamic effects. Whether exposed to laser irradiation or treated with the nanoprobe alone, cells remain viable, validating the superior biocompatibility of the nanoprobe.

### 2.5 Physical and Biological Mechanisms Underlying the Synergy

**FIGURE 6:**
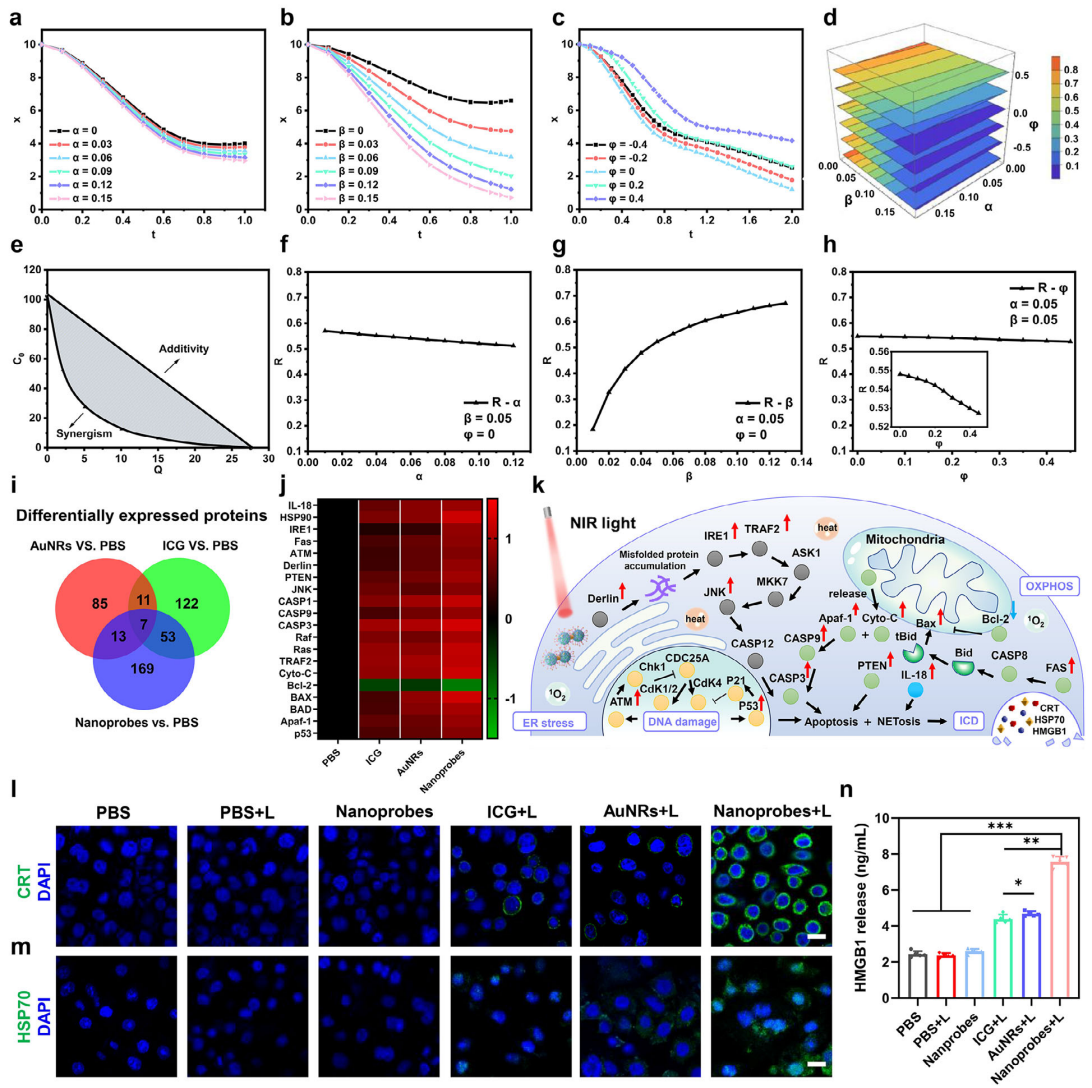
Analysis of mathematical modeling, proteomics, and validation. (a‐c) Schematic diagram of curves showing the change of tumor cell number density with treatment time under different α, β, and φ values, respectively; (d) Schematic diagram of the section view of N/K with φ, β, and α as variables; (e) Schematic diagram of the synergistic effect R = S_SA_ / S_∆_ based on the isobologram; (f‐h) Schematic diagram of the curve showing the effects of α, β, and φ values on R; (i) Venn analysis of differentially expressed proteins in four treatment groups under laser irradiation. Herein, ICG and AuNRs represent ICG molecules or AuNRs anchoring into mSiO_2_ and followed modification with NTP peptides; (j) A heatmap showing identified proteins contributing to cell death; (k) A schematic illustrating the network of possible pathways underlying the synergistic effects. (l) CLSM images of CRT exposure and (m) HSP70 expression (scale bar: 20 µm); (n) HMGB1 release from 5 to 8F cells after various treatments (n = 3). Mean ± SD, n = 3, *p < 0.05, **p < 0.01, ***p < 0.001, ns: not significant.

To further explore the synergistic effects of photothermal and photodynamic therapies on cells, we conducted both physical and biological investigations using mathematical modeling and proteomics experiments. Biological data were gathered from the photothermal‐photodynamic synergistic inactivation of NPC cells, from which key molecular biology parameters were derived. These parameters were then employed in simulations to model the synergistic interaction between PTT and PDT effects, elucidating both physical and biological synergistic factors. Utilizing a tumor growth model with a time delay and a pharmacokinetic approach based on the diffusion equation [30, 31], we optimized critical parameters influencing tumor treatment and photothermal‐photodynamic synergy. Specifically, we focused on three critical factors: the photothermal heating rate (α) of the photosensitizer (Figure 6a), the enhanced ROS production rate (β) of the photosensitizer under LSPR effects (Figure 6b), and the time delay (φ) between PDT and PTT (Figure 6c). These factors were assessed for their impact on the change in cellular density (N) during treatment. Additionally, the maximum environmental carrying capacity (K) was considered, with the ratio of N/K serving as a key indicator of treatment efficacy (Figure 6d; Figures S34 and S35). The N/K ratio decreases with increasing β and α, indicating a higher tumor cell kill rate and more effective treatment. For the treatment phase difference (φ), N/K is minimized at φ = 0, where there is no phase difference between PDT and PTT, correlating with the observed optimal tumor cell eradication. To validate the synergistic effects of photothermal and photodynamic therapies, we employed the Loewe additivity isobologram (Figure 6e). In this model, concave curves indicate synergistic effects between the two therapies, while straight lines represent an additive effect. The synergy is quantified by the ratio R = S_SA_ / S_∆_, where S_SA_ is the shaded area in Figure 6e and S_∆_ is the area of the triangle formed by the additive effect line and the coordinate axes. Figure 6f‐h shows the values of R under different α, β, and φ parameters, with β being the most sensitive parameter affecting R relative to α and φ. This suggests that the ROS generation rate (β) under plasmonic enhancement is a critical factor influencing the synergistic effect of photothermal‐photodynamic therapy, providing valuable guidance for optimizing the preparation parameters of the nanoprobe. Before applying the Allee model, we analyzed the photothermal‐photodynamic synergistic behavior using the commonly used logistic model. The results were consistent with those obtained from the Allee model, confirming the validity of our synergistic model (Figures S36–S38).

To further investigate the molecular mechanisms of photothermal‐photodynamic therapy, proteomics experiments were conducted to analyze the interactions between the nanoprobes and proteins. The Venn diagram summarizes differentially expressed proteins among various treatment groups, and the heatmap illustrates the upregulation and downregulation of key proteins related to cell death pathways (Figure 6i,j). Analysis reveals that the high temperatures and ROS generated by the combined photothermal‐photodynamic treatment disrupted the normal physiological balance of cells, inducing endoplasmic reticulum stress, oxidative stress, mitochondrial dysfunction, and DNA damage. These alterations trigger apoptosis, NETosis, and the release of ICD‐related signals, further activating the immune system to attack tumor cells (Figure 6k). Then we detected key damage‐associated molecular patterns (DAMPs) including surface‐exposed calreticulin (CRT), heat shock protein (HSP70), released high‐mobility group box 1 (HMGB1) as biomarkers of ICD (Figure 6l‐n). The relative fluorescence intensity of CRT and HSP70 was quantitatively analyzed (Figure S39), and the release level of HMGB1 was determined by quantitative detection of ELISA kit. The significant CRT expression, HSP70 exposure and HMGB1 release in tumor cells demonstrated that the nanoprobes induced strong ICD effects to boost immune responses. Furthermore, we conducted the transwell assay to investigate the influence of DAMPs exposure or cellular contents release on dendritic cell (DC) maturation. After co‐incubating DCs with 5–8f cells under various treatments, nanoprobes and laser treatment group promoted the CD80^+^ CD86^+^ DC population to 39.6%, which was significantly higher than that in the control group (Figure S40). All these results indicate that the nanoprobes upon laser irradiation could trigger potent immunogenicity and DC maturation, which would strongly activate downstream anti‐tumor immune pathways.

### 2.6 Photo‐Magnetic‐Acoustic‐Thermal Multimodal Imaging

**FIGURE 7:**
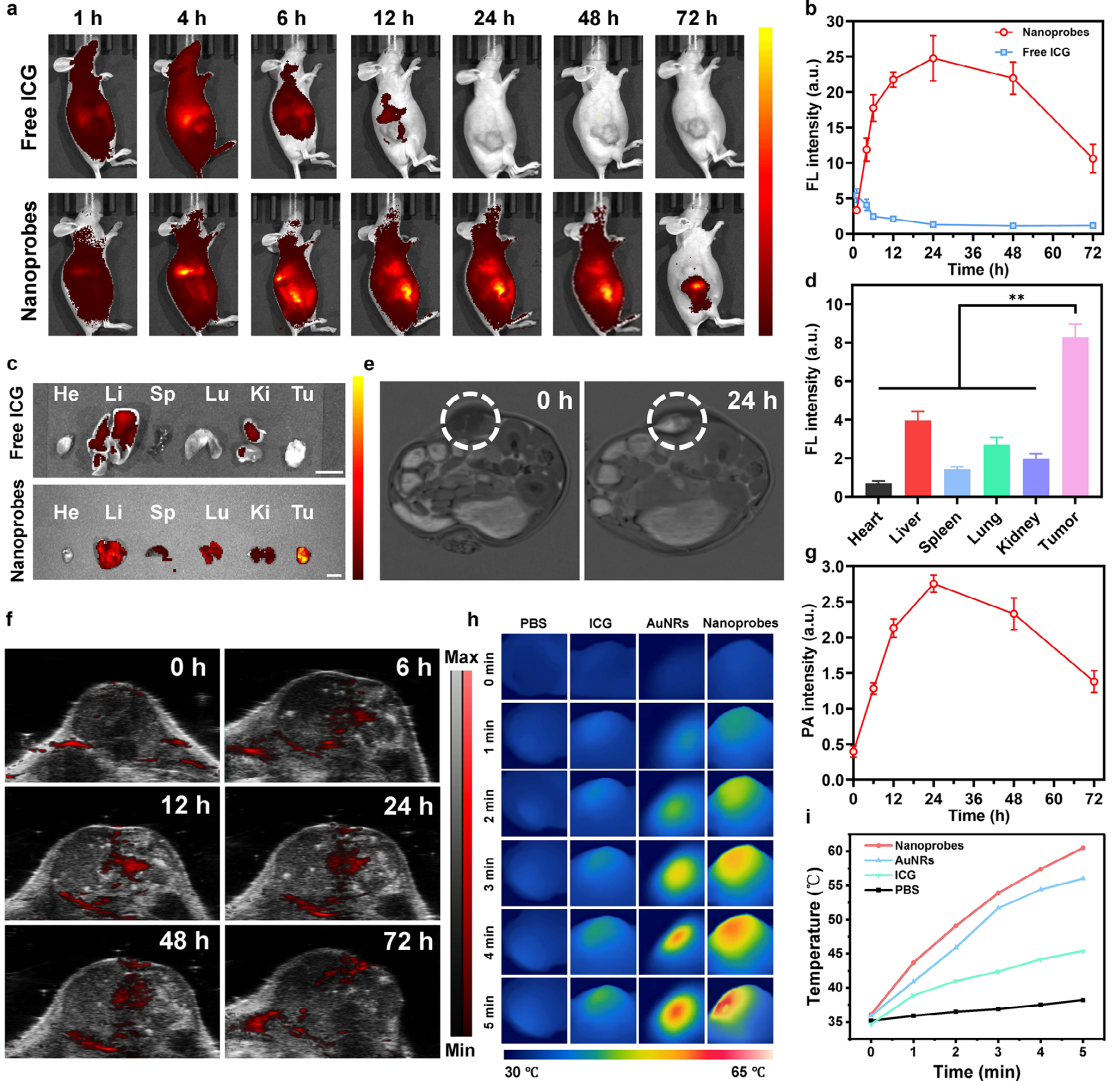
Multimodal imaging performance of nanoprobes in vivo. (a) Time‐dependent NIR fluorescence imaging and (b) the average fluorescence intensity of tumor‐bearing mice in 72 h after tail vein injection of the nanoprobe solution compared to the free ICG group; (c) NIR fluorescence imaging and (d) the average fluorescence intensity of tumor tissues and major organs including heart, liver, spleen, lung and kidney of tumor‐bearing mice 24 h after tail vein injection of the nanoprobe solution, scale bar: 1.0 cm; (e) Representative MRI images of tumor‐bearing mice 24 h after tail vein injection of the nanoprobe solution; (f) Time‐dependent NIR photoacoustic imaging and (g) the average photoacoustic signal intensity of tumor‐bearing mice in 72 h after tail vein injection of the nanoprobe solution; (h) Infrared thermal imaging and (i) temperature change curves of the tumors within 5 min under 808 nm laser irradiation (0.5 W/cm^2^) 24 h after tail vein injection of PBS, ICG, AuNRs and nanoprobes groups, Herein, ICG and AuNRs represent ICG molecules or AuNRs anchoring into mSiO_2_ and followed modification with NTP peptides. Mean ± SD, n = 5, *p < 0.05, **p < 0.01, ***p < 0.001, ns: not significant.

The composite nanoprobe exhibits exceptional PL properties in the NIR region (Figure S41), making it a promising tool for the early diagnosis of tumors. To evaluate its efficacy, we established an NPC‐xenografted mouse model, intravenously administered the optical nanoprobes, and conducted tumor‐targeted optical imaging (Figure 7a,b; Figure S42). Compared to the control group, the nanoprobes exhibit significant fluorescence enrichment at the tumor site. At 24 h post‐injection, the fluorescence signal is at its peak, indicating strong tumor targeting and accumulation capabilities. Notably, fluorescence signals remain detectable at 72 h. After sacrificing the mice at 24 h post‐injection, we excised various organs and tumor tissues. Fluorescence signals in the tumor were markedly stronger than in normal tissues (Figure 7c,d; Figure S43). Additionally, leveraging the MR relaxation enhancement of composite nanoprobes, we assessed the T_1_‐weighted MRI signal at the tumor site 24 h after intravenous injection. The MRI contrast was notably enhanced compared to the control group (Figure 7e). We also conducted time‐course NIR photoacoustic imaging on the NPC‐xenografted mice, observing that the photoacoustic signal intensity at the tumor site followed a similar temporal pattern to the in vivo fluorescence imaging, peaking at 24 h (Figure 7f,g). Given the excellent photothermal conversion efficiency of nanoprobes, we irradiated the tumor with an 808 nm laser (0.5 W/cm^2^) for 5 min and monitored the tumor using NIR photothermal imaging. The tumor temperature rapidly rose to 55°C (Figure 7h,i), demonstrating the potential of nanoprobes in PTT for synergistic use with PDT, effectively eliminating tumor tissue.

### 2.7 Therapeutic Effects of Nanoprobes in NPC‐Bearing Mice

**FIGURE 8:**
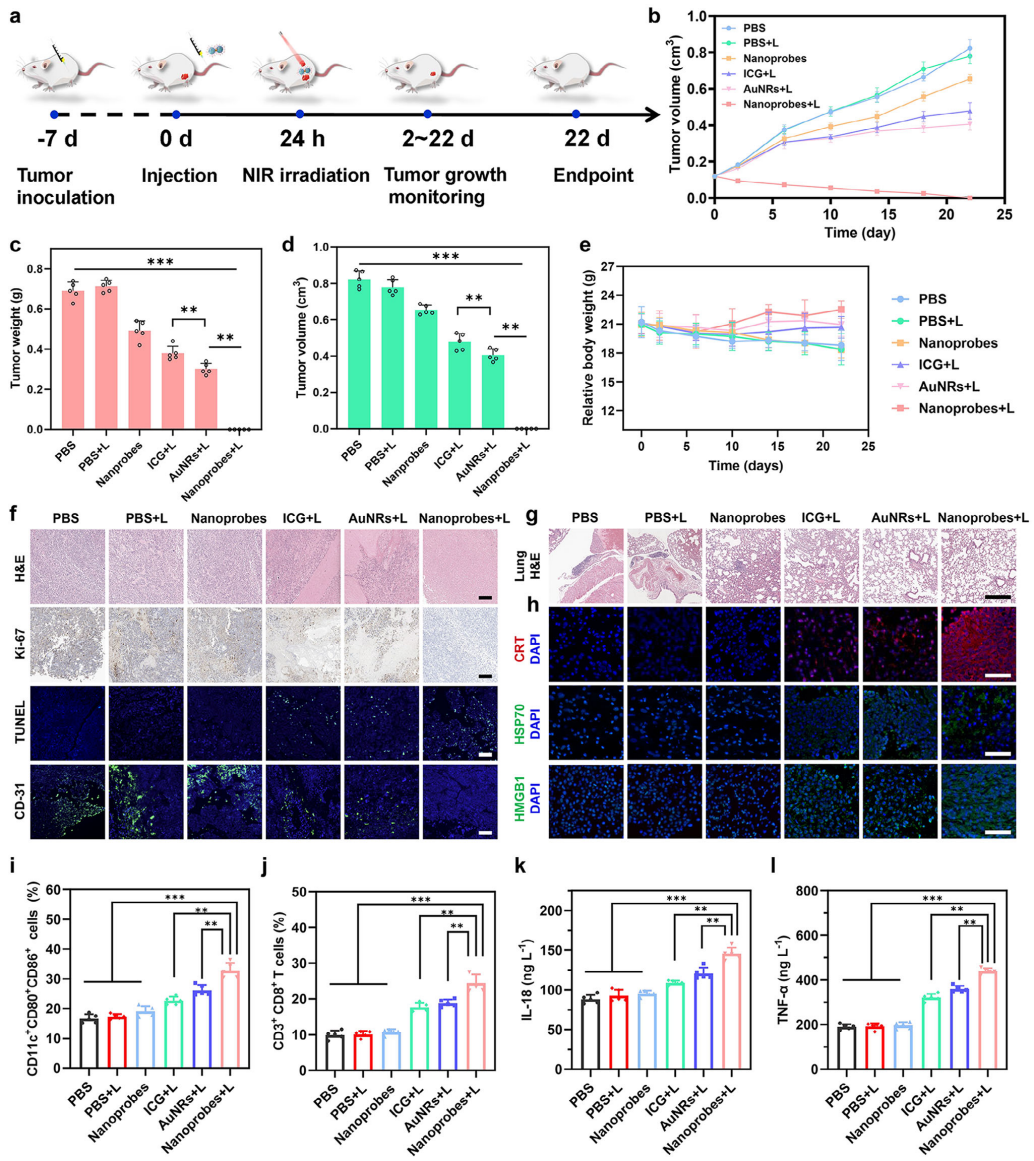
Therapeutic effects and immune responses of nanoprobes in vivo. (a) Schematic diagram of the treatment process for unilateral NPC‐bearing mice; (b) Time‐dependent average tumor growth curves of NPC‐bearing mice with different treatments; (c) Tumor weights and (d) tumor volumes of mice 22 days after different treatments; (e) Body weights of NPC‐bearing mice following different treatments; (f) H&E staining images, and Ki67, TUNEL, CD31 immunofluorescence images of tumor tissues after different treatments, scale bar: 200 µm; (g) H&E staining images of lung from sacrificed NPC‐bearing mice following different treatments, scale bar: 200 µm; (h) Immunofluorescence staining images of CRT, HSP70, and HMGB1 of tumor tissues after different treatments respectively, scale bar: 50 µm; Quantifications of (i) maturated dendritic cells (CD11c^+^CD80^+^CD86^+^) and (j) CD3^+^CD8^+^ cytotoxic T cells in NPC‐ bearing mice with different treatments analyzed with flow cytometry; Quantitative analysis of (k) IL‐18 and (l) TNF‐α in serum after different treatments. Mean ± SD, n = 5, *p < 0.05, **p < 0.01, ***p < 0.001.

NPC‐xenografted mice were established and subjected to a single session of phototherapy with 808 nm laser irradiation and nanoprobe treatment, when the tumor volume in NPC‐bearing mice reached 100 mm^3^. Nanoprobes solution was intravenously injected into mice, and the tumors received laser irradiation treatment 24 h later, followed by a three‐week observation period (Figure 8a). The tumor growth in the mice was significantly inhibited following low‐dose photothermal‐photodynamic therapy, with no recurrence over the three‐week period, and the tumors were completely eradicated (Figure 8b; Figure S44). In contrast, tumors in the control group show a marked growth trend. While individual PDT or PTT treatments led to partial tumor suppression, they were insufficient to fully eliminate the tumors, highlighting the efficacy of the synergistic treatment (Figure 8c,d; Figure S45). Moreover, the body weight of mice showed no significant differences among different groups, and all mice in nanoprobes and laser irradiation group remained healthy throughout the observation period, indicating excellent biocompatibility of nanoprobes (Figure 8e). Immunohistochemical and immunofluorescence analyses were performed on the tumor tissue. Hematoxylin and eosin (H&E) and Ki67 images reveal significant tumor necrosis and halt proliferation following treatment with the nanoprobe and laser irradiation, compared to the control group. TUNEL and CD31 immunofluorescence assays demonstrate marked apoptosis in tumor cells of treated mice, without evidence of angiogenesis (Figure 8f; Figure S46). Further immunohistochemical analysis of organs harvested from mice reveal lung metastasis in the control group, particularly in the PBS and laser‐only treated mice (Figure 8g). In contrast, the organs involving heart, liver, spleen, lungs, and kidneys of the cured mice appear normal, with no signs of deformation or necrosis (Figure S47). Immunofluorescence detection of ICD markers also reveals strong fluorescence signal changes for CRT, HMGB1, and HSP70 in tumor tissue following photothermal‐photodynamic treatment. The results confirm the activation of the immune system through ICD process, highlighting the capacity of nanoprobes to effectively inhibit NPC metastasis upon laser irradiation (Figure 8h; Figure S48).

To further explore the immune mechanisms induced by nanoprobes and laser irradiations in vivo, immunological analyses of the tumor‐infiltrating immune cells and related cytokines in serum were conducted. We analyzed the markers (CD80 and CD86) of mature DCs using flow cytometry (Figure 6i), and the percentage of mature DCs reached 32.8%, indicating that the nanoprobes and laser treatment remarkably promoted the maturation of DCs in tumors and tumor‐draining lymph nodes. In addition, CD8^+^ T cells and CD4^+^ T cells were also activated efficiently (Figure 6j; Figure S49), showing the excellent capability of systemic immune activation by primary tumor treatment. Simultaneously, we performed ELISA assays to evaluate the levels of pro‐inflammatory cytokines in terms of interleukin‐18 (IL‐18), tumor necrosis factor‐α (TNF‐α) and interferon‐γ (IFN‐γ) in serum. The secretion of these antitumor cytokines in nanoprobes and laser irradiation treatment group was significantly enhanced (Figure 6k,l; Figure S50). These results further highlight that nanoprobe‐mediated PTT‐PDT synergistic therapy could effectively stimulate the infiltration of immune cells and activate the systemic anti‐tumor immune responses in vivo.

## 3 Conclusion

In conclusion, we have developed a high‐performance composite optical nanoprobe for the synergistic treatment of NPC. By elaborately assembling mesoporous silica‐coated gold nanorods, rare earth‐doped gadolinium oxide nanocrystals, and the photosensitizer ICG, the dumbbell‐shaped composite nanoprobe exhibits significantly enhanced performance, including NIR fluorescence, photothermal conversion efficiency, photodynamic activity, photoacoustic, and magnetic resonance imaging. In vitro experiments demonstrate that under NIR laser irradiation, the nanoprobe generates ROS and induces hyperthermia, which significantly promotes NPC cell death. Theoretical simulations combined with experimental validation reveal that the LSPR effect of AuNRs is critical for enhancing the performance of nanoprobes and mediating photothermal‐photodynamic synergy. Quantum mechanical calculations further reveal Au elements induce charge transfer by transferring electron to ICG during electronic excitation, and together with rare earth Gd largely facilitate the multi‐path ISC process of ICG through the heavy atom effects, enhancing the photodynamic performance. Moreover, the nanoprobe possesses excellent biocompatibility, enables targeted and efficient internalization by NPC cells, and elicits no significant cytotoxicity. In vivo investigations confirm that the nanoprobes exhibit multimodal imaging involving fluorescence, photoacoustic, and magnetic resonance imaging of NPC‐xenografted mice and exert significant inhibition on tumor growth upon laser irradiations, with no tumor recurrence or distant metastasis observed. Further immunofluorescence analysis and evaluation of immune effects confirm that the nanoprobes promote immune system activation and enhance anti‐tumor immune responses. This study provides novel insights for the design of advanced high‐performance optical nanoprobes, offers an innovative strategy for the precision treatment of NPC, and holds substantial potential for clinical translation.

## 4 Materials and Methods

### 4.1 Assembly of Composite Optical Nanoprobes

The prepared ReNCs and dumbbell‐shaped AuNR@mSiO_2_ were integrated via an ultrasonic nano‐assembly technique. Before mixing them, we dispersed AuNR@mSiO_2_ in hydrochloric alcohol solution. The solution was refluxed at 60°C for 24 h, ensuring that the remaining CTAB residue was eliminated thoroughly. Then, we mixed 10 mL of AuNR@mSiO_2_ solution and 10 mL of ReNCs colloids, pouring them into a round‐bottom flask. The flask was fixed and continuously agitated in a water bath, and the mixture was intermittently sonicated at 37°C for 24 h. The assembled nanoparticles were collected and purified by three cycles of dispersing them ultrasonically in alcohol and deionized water, respectively, followed by specific centrifugation. The nanoparticle solution was concentrated into a centrifugal filter device to further remove impurities. The purified nanocomposites were dispersed in 30 mL of anhydrous toluene, and 0.5 mL of 3‐aminopropyltriethoxysilane (APTES) was added dropwise to the dispersion. The mixture was then refluxed with constant gently‐stirring overnight to achieve amination of the silica surface. After the reaction, the aminated nanoparticles were collected via centrifugation, thoroughly washed, and dried under vacuum.

For the ICG attachment, 10 mg of indocyanine green (ICG) was dissolved in 3 mL of dimethyl sulfoxide (DMSO). One milligram of dicyclohexylcarbodiimide (DCC) and 2 mg of N‐hydroxysuccinimide (NHS) were added as coupling agents, and the mixture was stirred at room temperature for 6 h to activate the sulfonic groups of ICG. Next, 50 mg of composite nanoparticle, dispersed in 2 mL of DMSO, was slowly added to the activated ICG solution. The solution was stirred continuously for 4 h to facilitate the sulfonylamidation reaction between amino groups on silica and activated sulfonic groups on ICG. Subsequent peptide conjugation was also achieved by reacting the amino‐functionalized particles with 50 µL of carboxylated nasopharyngeal carcinoma‐targeting peptide (NTP, 1 mg/mL) involving a small amount of DCC and NHS in darkness for another 8 h. The final product was purified through three cycles of centrifugation and stored in 10 mL deionized water at 4°C.

### 4.2 Photo‐Magnetic‐Acoustic‐Thermal Multimodal Imaging Assays

In vivo fluorescence imaging was conducted using IVIS Spectrum (PerkinElmer) instrument under the excitation wavelength ranging from 430 to 675 nm and the emission wavelength ranging from 720 to 840 nm. Acquired images were analyzed using the equipped Living image software (version 4.7.0).

For in vivo magnetic resonance imaging (MRI), NPC‐bearing mice were anesthetized by inhalation and then scanned using a 3.0 T T1‐MRI device equipped with a small animal‐specific coil. T1‐MRI imaging of the tumor was performed at different time points after the tail vein injection of nanoprobes. The scanning parameters are as follows: TR/TE = 500/8.4 ms, FOV = 50 × 50 mm^2^, matrix size = 252 × 248, number of averages = 4, scan time = 232 s.

For in vivo photoacoustic imaging, NPC‐bearing BALB/c mice were treated with nanoprobe solutions (100 µL, 20 mg/kg) through intravenous injection (n = 3), and PA imaging is accomplished by using an animal in vivo photoacoustic imaging system (Vevo LAZR, FUJIFILM Visual Sonics). PA images of the nanoprobe solutions at different concentrations were obtained under 808 nm laser irradiation. After injection with nanoprobe solutions, PA images of the tumor were collected 6, 12, 24, 48, and 72 h later, respectively.

Real‐time thermal imaging of the tumor site was conducted using an infrared thermal camera (TiS65, Fluke) to monitor temperature changes during treatment.

### 4.3 In Vivo Phototherapy Assays

The animal study protocol was approved by the Institutional Animal Care and Use Committee (IACUC) of Shenzhen University Medical School (Approval No. IACUC‐202500011). All experimental procedures were conducted in accordance with the relevant guidelines and regulations for animal welfare and ethics in China and Guangdong Province. Nasopharyngeal carcinoma xenograft models were established in BALB/c mice. When tumor volumes reached approximately 100 mm^3^, tumor‐bearing mice were randomly divided into six groups (n = 5 per group) for the following treatments: (1) PBS control group: Intravenous injection of 100 µL of PBS solution; (2) Laser‐only group: Localized 808 nm NIR laser irradiation (0.5 W/cm^2^, 5 min) with PBS administration; (3) Nanoprobes group without laser irradiation: Intravenous injection of nanoprobes solution (100 µL, 20 mg/kg); (4) ICG treatment group: Intravenous injection of NTP peptides‐modified mSiO_2_ (ICG) solution (100 µL, 20 mg/kg) followed by 808 nm laser irradiation (0.5 W/cm^2^, 5 min) 24 h post‐injection. (5) AuNRs treatment group: Intravenous injection of NTP peptides‐modified AuNR@mSiO_2_ solution (100 µL, 20 mg/kg) followed by 808 nm laser irradiation (0.5 W/cm^2^, 5 min) 24 h post‐injection. (6) Nanoprobes treatment group: Intravenous injection of nanoprobes solution (100 µL, 20 mg/kg) followed by localized 808 nm NIR laser irradiation (0.5 W/cm^2^, 5 min) 24 h post‐injection. Herein, ICG and AuNRs represent ICG molecules or AuNRs anchoring into mSiO_2_ and followed modification with NTP peptides.

Tumor dimensions were measured using digital calipers, with tumor volume calculated as (width^2^ × length)/2 and plotted against time. Tumor size and mouse body weights were recorded every other day to assess potential therapeutics efficiency. On day 22 post‐treatment, mice were humanely euthanized for terminal analysis, including tumor excision and weighing, as well as collection of major organs (heart, liver, spleen, lungs, and kidneys) for histopathological examination. In addition to monitoring tumor size and the physical condition of the mice, we also performed immunohistochemical (H&E staining) and immune‐fluorescence assays (Ki67, TUNEL, CRT, HSP70, and HMGB1 staining) on tumor tissues from mice receiving photoimmunotherapy 1–2 days post‐treatment. The humane endpoints for animal experiments were strictly implemented, including the criteria for euthanasia such as excessive body weight loss, severe behavioral abnormalities, obvious dyspnea, and failure to eat or drink independently.

### 4.4 In Vivo Analysis of Immune Effects

After different treatments of NPC‐bearing BALB/c white mice, the tumors or tumor draining lymph nodes (tdLNs) were collected and digested in RPMI 1640 media that contained collagenase and DNase at 37°C for 1 h. DCs harvested from tdLNs were stained with FITC‐CD11c, APC‐CD86, and PE‐CD80 for flow cytometry assay to evaluate DCs maturation. Cells in tumors were stained with PE‐CD3, FITC‐CD4, and APC‐CD8 to detect the CD8^+^ and CD4^+^ T cells levels by flow cytometry assay. Blood samples were collected from NPC‐bearing BALB/c white mice (n = 5) after 3 days of different treatments. The serum levels of IL‐18, TNF‐α, and IFN‐γ were measured by corresponding ELISA kit.

### 4.5 Additional Experiments

Other detailed experimental methods were listed in the Experimental and calculation methods of the Supporting Information. The preparation of rare earth‐doped gadolinium oxide nanocrystals and mesoporous silica‐coated mesoporous silica‐coated gold nanorods is shown in Section 1. Quantum mechanical calculations, excited‐state analysis, and numerical simulations of electromagnetic field are presented in Section 2. Cell experiments and proteomics analysis are described in Section 3. Mathematical modeling methods are provided in Section 4.

### 4.6 Statistical Analysis

Experimental data are presented as mean ± standard deviation (SD). Statistical analysis and quantitative graph generation were performed using GraphPad Prism 8 and Origin 2018 software. One‐way analysis of variance (ANOVA) followed by Tukey's post hoc test was applied to determine significance levels. Statistical significance was assumed at a value of *p < 0.05, **p < 0.01, ***p < 0.001. The detailed statistical analysis was described in Section S5 of experimental and calculation methods in the Supporting Information.

## Funding

This work was financially supported by the Guangdong Basic and Applied Basic Research Foundation (2024A1515030116, 2022A1515010006 and 2020A1515010426), the National Natural Science Foundation of China (62005322), IBME, GDAS Director's Fund Project (0525185001) and GDAS' Project of Science and Technology Development (2023GDASZH‐2023010102).

## Conflicts of Interest

None of the authors have a conflict of interest to disclose.

## Supporting information




**Supporting File**: advs75357‐sup‐0001‐SuppMat.pdf.

## Data Availability

The data that supports the findings of this study are available in the supplementary material of this article.
