# Supplementary material for: Dumbbell‐Structured Plasmonic‐Enhanced Optical Nanoprobes Boosting Photo‐Magnetic‐Acoustic Multimodal Imaging‐Guided Photodynamic‐Photothermal Synergistic Treatment and Immunogenic Death in Nasopharyngeal Carcinoma
Source: Adv Sci (Weinh). 2026 Apr 16;13(39):e75357. doi: 10.1002/advs.75357 (PMC13335434; doi:10.1002/advs.75357)
Supplement: Supplementary file 1 — Supporting File: advs75357‐sup‐0001‐SuppMat.pdf. [file ADVS-13-e75357-s001.pdf]

Supplementary Information

Dumbbell-structured plasmonic-enhanced optical nanoprobes boosting photo-magnetic-acoustic multimodal imaging-guided photodynamic-photothermal synergistic treatment and immunogenic death in nasopharyngeal carcinoma

Baikang Zhuang[1], Yubiao Yang[1], Wen Han[1], Yi Tang[1], Chengxin Yao[1], Fuli Zhao[2], Wenxiao Fang[3], Jinjie Li[4], Xiaolan Huo[1], Yiqian An[1], Yuanzhi Shao[3,*], Botao Gao[4,*] and Jinchang Yin[1,*]

[1]*Guangdong Key Laboratory for Biomedical Measurements and Ultrasound Imaging, National-Regional Key Technology Engineering Laboratory for Medical Ultrasound, School of Biomedical Engineering, Shenzhen University Medical School, Shenzhen University, Shenzhen 518060, China*

[2]*State Key Laboratory of Optoelectronic Materials and Technologies, School of Physics, Sun Yat-sen University, Guangzhou, 510275, China*

[3]*School of Systems Science and Engineering, School of Science, Sun Yat-sen University, Shenzhen 518107, China*

[4]*Institute of Biological and Medical Engineering, Guangdong Academy of Sciences, Guangzhou 510632, China*

**Corresponding authors. E-mail addresses: yinjch6@szu.edu.cn (Jinchang Yin), gaobotao1984@outlook.com (Botao Gao), stssyz@mail.sysu.edu.cn (Yuanzhi Shao)*

B.Z. and Y.Y. contributed equally to this work.

Experimental and calculation methods

Section 1: Preparation of nanoprobes

1. Fabrication of ultrafine rare earth-doped gadolinium oxide nanocrystals

Ultrafine rare earth-doped gadolinium oxide nanocrystals (ReNCs) were prepared via direct colloidal precipitation using high-boiling-point polyols. Typically, 600 mg of mixed rare earth nitrate was dissolved in 10 ml of diethylene glycol (DEG). The molar ratio of Gd: Yb: Er: Ho is 92: 6: 1: 1. After the resulting solution was vigorously stirred at 800 rpm for 1 h at 80 °C, 1.125 mL of aqueous NaOH solution (1 mmol L^{-1}) was added quickly. The mixture was stirred at 140 °C for 1 hour, followed by 180 °C for 4 hours. The resulting ReNCs were purified via three cycles of ultrasonic dispersion and centrifugation with acidic ethanol and deionized water, The transparent colloidal product was diluted to 1% with pure water and preserved at 4 °C.

2. Synthesis of dumbbell-shaped mesoporous silica-coated gold nanorods

Mesoporous silica-coated gold nanorods (AuNR@mSiO_2) nanocomposite precursors were synthesized through a three-step protocol. First, monodisperse gold nanorods (AuNRs) were prepared via a seed-mediated growth approach. For the seed solution, 0.364 g of cetyltrimethylammonium bromide (CTAB) was dissolved in 8.34 mL of deionized water. To this solution, 0.5 mL of $HAuCl_4$ (6 mM) and 1.16 mL of ice-cold $NaBH_4$ (10 mM) were sequentially added. After vigorous stirring for 2 min, the mixture was incubated in a water bath at 27 °C for 2 h. For the growth solution, 1.82 g of CTAB was dissolved in 42 mL of deionized water; under constant stirring, 6 mL of $HAuCl_4$ (6 mM), 1 mL of concentrated HCl (37%), and 1.2 mL of ascorbic acid (50

mM) was added. Finally, 50 μL of the seed solution was introduced into the growth solution, followed by gentle stirring for 10 s, and the mixture was incubated in a 27 °C water bath for 12 h.

The resulting AuNRs were collected by centrifugation, washed, and then encapsulated with silica via a sol–gel method. 20 mL of the AuNRs solution was ultrasonically diluted with 10 mL of deionized water, and 0.1 mL of NaOH solution (0.030 mol/L) was added. Under gentle stirring (100 rpm), 0.04 mL of a 20% tetraethyl orthosilicate (TEOS) ethanol solution was injected every 30 min for 2 h, followed by continuous stirring for another 12 h. The AuNRs coated with an ultrathin silica layer was collected by centrifugation and alternately washed with ethanol and deionized water twice. Subsequently, 20 mL of the solution was ultrasonically diluted with 20 mL of deionized water, along with 0.029 g of CTAB and 0.6 mL of NaOH solution (0.030 mol/L). Under gentle stirring, 0.8 mL of 20% TEOS ethanol solution was added in four aliquots (one every 30 min), and the mixture was further stirred for 12 h. The resulting AuNR@mSiO_2 was purified via three cycles of ultrasonic dispersion and centrifugation: first in NH_4NO_3 solution, then in hot acidic ethanol, and finally in deionized water to remove the CTAB template.

The dumbbell structure and morphology of the as-prepared AuNR@mSiO_2 nanoparticles were characterized using a FEI Tecnai-G2 Spirit transmission electron microscope (TEM) equipped with a field emission gun, operating at an acceleration voltage of 120 kV. The absorption spectra of the nano-dumbbell solutions (loaded in 1 cm quartz cuvettes) were recorded using a UV-visible-NIR spectrophotometer (UV-

3150).

Section 2: Theoretical calculations and numerical simulations

1. Calculating the interaction between Gd_2O_3 and SiO_2 based on first principles

To prove that the Gd_2O_3 particles would not go out of the mesoporous silica system, the theoretical study was performed. According to the previous works of our laboratory [S1,2], the similar systems have been researched. To further explore the interaction of Gd_2O_3 particles and silica, several modifications were made.

In the beginning, an amorphous silica model file was imported. Considering the limited calculation capability, a silica model sized $12.2349\ \text{Å} \times 12.2349\ \text{Å} \times 30\ \text{Å}$ is selected randomly from the initial model. The geometry optimization was operated after a few modifications, which include adjusting hydrogen, deleting irrational structures like H_2O and Si atoms with three hydroxyls and add O atoms to Si atoms which are linked with fewer than four O atoms. In geometry optimization, Perdew-Burke-Ernzerhof exchange-correlation potential (GGA-PBE) [S3] was used. Monkhorst-Pack grid was set as corse. The basis set was DN. The convergence tolerance was set as follows: 10^{-4} Ha (Hartree, equals to 27.2114 eV) for total energy, 0.02 Ha/ Å for max force and 0.05 Å for max displacement. Smearing was set as 0.05 to realize convergence. Then, a thin layer of gold base was added and optimized.

The second step was to add Gd_6O_9 cluster and optimize. The parameters were the same as previous. Three orientations of the cluster were modeled, which were demonstrated in Figs. S5-7, marked as Gd-O (Fig. S5), Gd-OH (Fig. S6) and O-H (Fig.

S7) respectively. As the name indicates, Gd-O means a gadolinium atom in Gd_6O_9 cluster approaching the oxygen atom in SiO_2, Gd-OH means a gadolinium atom in Gd_6O_9 cluster approaching the oxygen atom in hydroxyl on the surface of SiO_2, and O-H means an oxygen atom in Gd_6O_9 cluster approaching the hydrogen atom in hydroxyl on the surface of SiO_2.

In the finishing process, the electronic properties were calculated respectively in three models. GGA-PBE was adopted, Monkhorst-Pack grid was set as $3 \times 3 \times 1$, The basis set was DND. The convergence tolerance was set as 10^{-6} Ha for total energy. Smearing was set as 0.05 to realize convergence.

2. Calculating the interaction between ICG and SiO_2 based on quantum mechanics

We employed semi-empirical quantum mechanics (SQM) to calculated the interaction between ICG and SiO_2. A commonly used approach within this domain is QM method based on solving the Schrödinger equation. While QM methods are undoubtedly accurate in theory, they require significant computational time when applied to excessively large systems. Consequently, they cannot balance accuracy and computational time simultaneously for large-scale systems. In contrast, SQM methods, which incorporate empirical parameters, strike a balance between time and computational cost and accuracy, thereby achieving an approximate yet efficient approach. First, various photosensitizer components were independently constructed, including ICG, silica surface (short), silica surface (long), and various gadolinium oxide molecules. It was ensured that each component achieved successful structural

optimization, i.e., all four criteria-maximum atomic force, root-mean-square (RMS) atomic force, maximum atomic displacement, and RMS atomic displacement converged. To investigate the interaction relationships between components and the influence on functional groups, the components were combined and subjected to structural optimization using semi-empirical quantum mechanics methods. The calculation files were exported; weak interactions were analyzed using Multiwfn, and visualization was performed using VMD.

3. Excited-state calculations of gold nanorods

First, a gold nanorod was constructed via VMD modeling, characterized by an absorption peak close to that of the experimentally obtained gold nanorod and a relatively small atomic weight to facilitate calculations. Subsequently, the excited states of the gold nanorod were computed using the CAM-B3LYP functional. Thereafter, the absorption spectra of the gold nanorod and electron-hole transfer images were generated using Multiwfn software. Due to the distinct separation of electrons and holes in plasmon resonance absorption, with their distribution predominantly localized at both ends, calculations of electron-hole transfer enable a mechanistic explanation of whether the absorption peak arises from the plasmonic effect, followed by corresponding analysis.

4. Numerical simulations

The electromagnetic field around the nanoprobe was calculated using the time domain finite-difference algorithm. The complex dielectric constants of gold were taken from the literature of Johnson and Christy [S4]. The size and structure parameters

of AuNR@mSiO$_2$ (ReNCs&ICG) were created referring to transmission electron spectroscopy (TEM) measurements. The background was set as water. The refractive index of water (H_2O), silica (SiO_2), ReNCs and ICG ($C_{43}H_{48}N2O_6S_2.Na$) are set as 1.33, 1.5, 1.8 and 1.65, respectively, referred from Palik's handbook [S5]. The silica template of the dumbbell ball was modeled as a periodic structure with a mesoporous unit cell. The ordered water pores were randomly set inside the silica matrix with refractive index of 1.33. A perfectly matched layer (PML) boundary conditions and total-field scattered-field (TFSF) sources (50 fs) were employed. The monitors were used to record electric field intensity profiles.

5. Excited-state calculations of ICG in the presence of Au and Gd

To locate the global energy minimum of Indocyanine Green (ICG), conformational searches have been conducted and a series of rational methods have been adopted. Molecular dynamics (MD) simulations were performed using the semi-empirical program package xtb to generate 2000 initial guess structures [S6]. The simulation duration was set to 100 picoseconds (ps), and the simulation temperature was 400 Kelvin (K). The obtained initial guess structures were optimized in batches and sequentially using the GFN0-xTB and GFN2-xTB methods, aiming to eliminate conformations with relatively high energies [S7]. At the B3LYP/6-31G level* and combined with the Grimme D3BJ dispersion correction (abbreviated as GD3BJ), the remaining structures were optimized via density functional theory (DFT) calculations to obtain the thermal correction values of Gibbs free energy [S8]. The Solvation Model Based on Density (SMD) was employed to account for the solvent effect of water [S9]. Meanwhile, frequency calculations were performed at the same functional and basis set

level to obtain reliable structures without imaginary frequencies. Using the ORCA program, single-point energy calculations were carried out on the optimized structures with the double-hybrid functional and quadruple-ζ basis set (def2-QZVPP) to improve calculation accuracy [S10-12]. The implicit solvent model based on SMD was also used in this process to simulate the solvent environment. The molecular geometric conformations were ranked according to the Gibbs free energy, which was calculated as the sum of the single-point energy and the thermal correction value of Gibbs free energy. Additionally, the Molclus software was used to determine the major conformations at room temperature via Boltzmann distribution calculations [S13].

The calculation of the absorption spectra of ICG was performed at the CAM-B3LYP/Def2-TZVP level [S11,14], incorporating the GD3BJ correction and the SMD solvent model for water. For the calculation of ICG's emission spectra, it was based on the optimized S_1 excited state structure, using the CAM-B3LYP/Def2-TZVP level with the inclusion of the GD3BJ correction and the SMD solvent model for water. In order to make the calculation results better match the experimental results, we manually blue-shifted the absorption spectrum by 20 nm, approximately 0.04 eV, and manually red-shifted the calculated emission spectrum by 10 nm.

Based on the optimized structure of ICG and with reference to the prepared nanocomposite, the simplified structure of AuNR@SiO_2@Gd_2O_3/ICG (abbreviated as Gd_Au/ICG) was modeled. The structures of ICG and Gd_Au/ICG were further optimized using the PBE0 exchange-correlation functional [S15]. Specifically, for gadolinium (Gd) atoms, the quasi-relativistic effective core potential (ECP53MWB) including 53 core electrons was employed, accompanied by the corresponding (7s6p5d)/[5s4p3d] (Gd) basis set [S16]; for Au atoms, the ECP60MWB effective core potential and its corresponding basis set were used; for all other atoms (C, H, O, N, Si,

and S), the Def2-TZVP basis set was adopted. At the same computational level, the stability of these optimized structures (corresponding to local energy minima) was verified by confirming the absence of imaginary frequencies in the structures.

Based on the ground-state optimized structures, 10 low-lying singlet excited states and 10 low-lying triplet excited states of ICG and Gd_Au/ICG were calculated via time-dependent density functional theory (TD-DFT) at the PBE0/def2-TZVP/ECP53MWB/ ECP60MWB level. Subsequent analyses and calculations were performed using the Multiwfn program [S17], including: Analyses of density of states (DOS), HOMO-LUMO orbitals, hole-electron distributions, charge density difference (CDD), and transition density matrix (TDM) plots; calculations of various parameters such as HOMO-LUMO energy gap, ΔES_1T_n, D index, and Sr index. Among these, the partial density of states (PDOS) was plotted based on the C-squared population analysis method in main function 10 of the Multiwfn program, realized through the contributions of basis functions to molecular orbitals (MOs) [S17]. Angular momentum P in the defined fragments (e.g., right or left Indole rings, polyacetylene chain, Au atoms) and peripheral groups were further specified to investigate their effect on the total DOS. Additionally, Gaussian functions were used to broaden all DOS corresponding to discrete molecular orbital energy levels within the energy range of -8 to 1.3 eV, to more intuitively illustrate the relationships between PDOS. The related isosurface maps were generated using the Visual Molecular Dynamics (VMD) software [S18].

The spin-orbit coupling (SOC) matrix was obtained using the ORCA program. All-electron calculations were performed, incorporating the second-order Douglas-Kroll-Hess (DKH2) method [S19]. For Gd and Au atoms, the segmented all-electron relativistically contracted (SARC) basis sets [S20] were employed, in conjunction with the DKH-recontracted def2-TZVP basis sets; all other atoms utilized the DKH-def2-

TZVP basis sets. The calculations adopted the PBE0 functional with D3BJ correction, and the auxiliary basis sets utilized the SARC/J Coulomb-fitting basis sets. To accelerate the computation, the resolution-of-identity-chain-of-sphere exchange (RIJCOSX) approximation [S21] was adopted, and the COSX grid size was set to the specifications corresponding to the program keywords GridX6 and NoFinalGridX. The SMD model was used to account for solvent (water) effects.

Section 3: Cell experiments and proteomics analysis

1. Cellular Culture and uptake

Poorly differentiated nasopharyngeal squamous cell carcinoma (CNE2, RRID: CVCL_6889) and highly metastatic nasopharyngeal carcinoma (5-8F, RRID: CVCL_C528) cell lines were obtained from Shanghai Jinyuan Biotechnology Co., Ltd. (Shanghai, China). These cells were incubated in RPMI 1640 (Gibco) containing 10% fetal bovine serum (FBS) and antibiotics; normal nasopharyngeal epithelium NP69 cells were cultured in keratinocyte serum-free medium (K-SFM, Gibco) with bovine pituitary extract (BPE, 0.05 mg/mL, Gibco), epidermal growth factor (EGF, 0.005 μg/mL, Gibco) and antibiotics and maintained at 37 °C in a humidified atmosphere containing 5% CO_2. Cells in logarithmic growth phase were digested using 0.25% trypsin (Gibco) and seeded in 35-mm Petri dishes with 20 mm glass bottom wells at a density of 1×10^6 cells per dish and incubated for 24 h. The dishes were washed three times with phosphate-buffered saline (PBS) and replaced with fresh medium containing 10 μM of the nanoprobe solution for another 4 h incubation. In addition, blank control cells were supplemented with fresh media containing no nanoprobes. After that, the

adherent cells were then washed three times with PBS to remove residual nanoprobes and dead cells and subjected to fluorescence confocal microscopy imaging. Cell samples were excited by a 405 nm laser and recorded at detection channel of 633 nm for the nanoprobes using a Leica TCS SP5 confocal laser scanning microscope (Leica Inc., America).

2. Live/dead cell imaging and ROS detection in tumor cells

NPC cells were seeded in 96-well plates at a density of 1.0×10^4 cells/well and allowed to adhere overnight. Cells were treated with PBS (control), ICG solution (10 μM), AuNRs solution (10 μM) and nanoprobes solution (10 μM) for 2 h, followed by 808 nm NIR laser irradiation (0.5 W/cm^2, 5 min) to induce reactive oxygen species (ROS) generation. For ROS detection, hydrogen peroxide (H_2O_2)-treated cells were set as the positive control. Cells were incubated with 5 μM of DCFH-DA for 30 min after 5 min laser irradiation, washed with PBS, and fluorescence intensity was measured using a microplate reader (excitation: 488 nm; emission: 525 nm). Cell viability was assessed by adding CCK-8 reagent after 4 h post-treatment, then incubating for 2 h, and measuring absorbance at 450 nm.

Live/dead cell imaging was performed with nanoprobes-treated cells, stained with 2 μM of Calcein-AM and 4.5 of μM propidium iodide (PI) for 15 minutes and imaged using a confocal microscope (Leica SP8). Live cells showed green fluorescence (excitation: 488 nm, emission: 500-550 nm) while dead cells showed red fluorescence (excitation: 561 nm, emission: 570-620 nm). All experiments were performed in triplicate.

The temperature was monitored by photothermal imaging when the cells were treated with 808 nm laser (0.5W/cm^{-2}) every minute using an infrared thermal camera (Tis65, Fluke).

3. Proteomics analysis

Tumor cell samples with treatments of irradiation of 808 nm laser (0.5 W/cm^2, 5 min) from the PBS, ICG, AuNRs, nanoprobes groups (n = 3) were collected and subjected to quantitative proteomics analysis at FitGene (Guangzhou, China). Proteins were considered to be differentially expressed if the fold-change between the experimental and control groups was greater than 2 or less than 0.5 ($p < 0.05$). Herein, ICG and AuNRs represent ICG molecules or AuNRs anchoring into $mSiO_2$ and followed modification with NTP peptides.

4. *In Vitro* Detection of Immunogenic Cell Death (ICD)

NPC cells were seeded and treated with PBS (control), ICG solution (10 μM), AuNRs solution (10 μM) and nanoprobes solution (10 μM) for 2 h, followed by 808 nm NIR laser irradiation (0.5 W/cm^2, 5 min). After different treatments, the cells were incubated for another 16 h. Then the cells were washed by PBS, fixed with 4% paraformaldehyde and permeabilized with 0.1% Triton X-100, blocked with 2% BSA, incubated with anti-CRT antibody or anti-HSP70 antibody for 2h and the secondary antibody (FITC IgG) for 1 h. Finally, the cells were counterstained with DAPI and observed under CLSM. The extracellular released HMGB1 proteins were detected by an ELISA kit.

5. *In vitro dendritic cell stimulation experiments*

5-8Fcells were seeded in upper compartment of the transwell system and incubated

with 40 μg/mL nanoprobes for 2 h, with or without 808 nm NIR laser irradiation (0.5 W/cm^2, 5 min). Then the immature DCs were added in the lower compartment and cocultured for 24 h. After different treatments, DCs were stained with PE anti-mouse CD86, APC anti-mouse CD80 for 30 min at 4 °C and DC maturation status was analyzed by a flow cytometry.

Section 4: Mathematical modeling and methods

Tumor growth is analogous to population growth in ecosystems and can be well described by population dynamics within ecosystems. Population dynamics in ecosystems describes the evolutionary laws governing changes in population size within ecological environments over time. The Logistic model is one of the most classical models in population dynamics, and its formulation is as follows [S22]:

$$\frac{dx}{dt} = r_0 x(1 - \frac{x}{K}) \tag{1}$$

Here, x denotes the population density; dx/dt represents the instantaneous rate of change of population density with respect to time; r_0 denotes the intrinsic growth rate of the population; and K denotes the carrying capacity of the environment. The model assumes that the population is spatially uniformly distributed, and that the relative growth rate of the population decreases monotonically and linearly as the population size increases, finally dropping to zero when the population density reaches the carrying capacity of the environment, K.

The classical Logistic model cannot fully and accurately describe the population dynamics of ecological systems in real-world scenarios. One of the reasons is that in practical systems, the current state of the system may depend on its state a period of time prior, namely, a time delay exists. For instance, newborn individuals in a population lack reproductive capacity and require a certain period of time to develop and reach sexual maturity.

Tumor cell division also exhibits a cycle: newly divided cells require one full cell

cycle to divide again and generate subsequent new cells. Therefore, to more accurately describe the tumor growth process, we need a modified Logistic model that can account for the time delay phenomenon. The general form of a Logistic model capable of describing time delay is given by:

$$\frac{dx(t)}{dt} = r_0 x(t)(1 - \frac{\int_{-\infty}^{t} \omega(t-s)x(s)ds}{K}) \tag{2}$$

In general, ω is a Gaussian function with a mean value of T. When the second moment of ω tends to zero, the Gaussian function evolves into a δ function Correspondingly, the form of the equation transforms to:

$$\frac{dx(t)}{dt} = r_0 x(t)(1 - \frac{x(t-\tau)}{K}) \tag{3}$$

Without considering the actual spatial distribution of the tumor and assuming that the tumor is spatially uniformly distributed, tumor growth per unit space can be described by the time-delayed Logistic model, where τ denotes the time delay induced by the cell cycle of tumor cells.

In population dynamics modeling, the most commonly used model is the classical Logistic growth model. The model accounts for the finiteness of living space and environmental resources, which cannot provide the required space and energy base for an infinite number of population individuals. Specifically, each population has a corresponding environmental carrying capacity within its residing ecosystem: if the population density is too high, the average resources per individual of that population will decrease. Additionally, excessively high population density will further cause environmental deterioration, thereby leading to phenomena such as an increase in

diseases. The consequence of these factors is a decrease in the population's birth rate and an increase in its death rate, ultimately resulting in a decline in population density. However, the Logistic growth model only accounts for constraints on population development imposed by external factors such as limited spatial resources and environmental deterioration; it fails to consider the impact of cooperation among individuals between populations on the population itself. That is to say, the fitness of individuals between populations and their cooperative behaviors should also be taken into account.

The Allee model addresses the limitations of the Logistic model. Populations exhibiting the Allee effect incorporate the cooperative behavior among individuals within the population, meaning that a minimum population density (sufficient to sustain survival) is required to ensure individuals have adequate conditions and opportunities to cooperate. When the population density falls below a certain threshold, the excessively low density leads to a lack of intrapopulation cooperation and a subsequent decrease in individual fitness, which causes the population to tend toward extinction.

By ignoring factors such as the heterogeneous spatial distribution of tumors and variations among homogeneous tumor cells, tumor growth per unit space can be described by the time-delayed Allee model:

$$\frac{dx(t)}{dt} = r_0 x(t)\left(1 - \frac{x(t-\tau)}{K}\right)\left(\frac{x(t-\tau)}{A} - 1\right) \quad (4)$$

Here, A denotes the Allee effect intensity, which is associated with the intrinsic factors

of the tumor.

Photothermal Therapy (PTT) and Photodynamic Therapy (PDT) share a similar therapeutic process: first, a photothermal converter or photosensitizer is administered to the patient; subsequently, after the agent has specifically accumulated in the tumor tissue, the tumor is irradiated with a laser, triggering a photothermal effect or a photodynamic reaction, respectively, for the purpose of eradicating the tumor. Therefore, these two therapies are naturally well-suited for combination. NIR dyes used in PDT can often double as photothermal converters for PTT. For instance, ICG and Prussian Blue have also been utilized as organic photothermal converters [S23].

Noble metal nanoparticles acting as photothermal converters also exhibit an enhancing effect on PDT [S24]. When these nanoparticles are irradiated with light of a specific frequency, the free electrons within them are driven by the light and undergo resonance on the particle surface, a phenomenon known as surface plasmons. The resonant free electrons generate strong electromagnetic fields near the metal surface. Photosensitizer molecules in the vicinity of the metal nanoparticles acquire energy from these electromagnetic fields and transition to an excited state, then transfer energy or electrons to oxygen molecules to generate ROS. This is the mechanism by which surface plasmons promote the photodynamic reaction.

By constructing composite-structured nanoparticles, a favorable photothermal-photodynamic synergistic effect can be achieved. For instance, composite nanoparticles with AuNRs as the core are coated with mesoporous silica on the surface; ICG, which acts as the photosensitizer, is encapsulated in the pores of the silica layer [S25]. ICG is

unstable under conditions such as aqueous solutions and light irradiation; encapsulating it within the silica layer can slow down its decomposition.

Meanwhile, confining the photosensitizer to the vicinity of AuNRs enables the vast majority of the photosensitizer to be excited by the localized electromagnetic fields generated by surface plasmons, thereby achieving a favorable photothermal-photodynamic synergistic effect. Assume that "core-shell" composite nanoparticles, consisting of noble metal nanoparticles as the core and a shell containing organic molecular photosensitizers, are used as the therapeutic agent, where all photosensitizers can be enhanced by the surface plasmons of the metal core. Additionally, it is assumed that the nanoparticles are uniformly distributed in tumor tissues, and the external laser light source is constant and irradiates uniformly.

To construct a model for the treatment process, a therapeutic killing term for tumor cells is introduced into the Allee model. For simplification, we can assume that the dose-response relationship curve is linear [S22]. After introducing the linear therapeutic term, the form of the equation is as follows:

$$\frac{dx(t)}{dt} = r_0 x(t)\left(1 - \frac{x(t-\tau)}{K}\right)\left(\frac{x(t-\tau)}{A} - 1\right) - \mu_H H(t)x(t) - \mu_B B(t-\varphi)x(t) \quad (5)$$

Here, A denotes the actual PTT intensity, and B denotes the actual PDT intensity; μ_H represents the killing rate of tumor cells by PTT, and μ_B represents the killing rate of tumor cells by PDT; φ stands for the time delay of the PDT process relative to the PTT process, i.e., the phase difference in their therapeutic effects between the two therapies. The value of B is directly related to both the heat generated per unit space and ROS.

While the heat conduction equation and the diffusion equation describe heat transfer and mass diffusion, respectively, their mathematical forms are actually identical. Since first-order pharmacokinetics is derived from the diffusion equation, the changes in A and B can thus be described by first-order pharmacokinetics:

$$\frac{dH(t)}{dt} = Q + \alpha C(t) - \lambda_H H(t) - \gamma_H x(t) H(t) \quad (6)$$

$$\frac{dB(t)}{dt} = \beta_C C(t) + \beta QC(t) - \lambda_B B(t) - \gamma_B x(t) B(t) \quad (7)$$

Wherein, Q is the heat source term, representing the constant heat generation by photothermal converters acting as heat sources under constant laser irradiation. α denotes the heat production rate of the photosensitizer due to photothermal effects, and C is the photosensitizer concentration. β stands for the ROS production rate of the photosensitizer without enhancement. Since the photothermal effect process of metal nanoparticles is directly related to surface plasmons, the increased ROS production rate of the photosensitizer under surface plasmon enhancement can actually be described by βQ. λ_H denotes the non-therapeutic loss rate of heat, and λ_B denotes the non-therapeutic loss rate of ROS. γ_H denotes the therapeutic process loss rate of heat, and γ_B denotes the therapeutic process loss rate of ROS.

Unstable organic photosensitizers such as Indocyanine Green (ICG) actually decompose over time following a negative exponential function of time under light irradiation[S26]. Thus, the equation describing C should be as follows:

$$\frac{dC(t)}{dt} = -\lambda_C C(t) \quad (8)$$

Assuming that the initial value of x is the maximum environmental carrying capacity

and the initial value of the photosensitizer concentration is C_0, the initial conditions of the established model are as follows:

$$x(0) = K, A(0) = 0, B(0) = 0, C(0) = C_0 \quad (9)$$

To investigate through simulation calculations the effects of factors represented by different parameters on both the therapeutic efficacy of combined photothermal and photodynamic therapy, and the synergy between photothermal therapy (using metal nanoparticles) and photodynamic therapy (using organic dyes), it is necessary to set baseline values for each parameter in the model. Based on previous work [S27,28], the environmental carrying capacity K can be set to 10, and the natural tumor growth rate r_0 to 1; reasonable value ranges for other parameters are determined accordingly. The finally determined baseline values of the parameters are shown in Table S1. Among these parameters, the Intrinsic growth rate of tumor cells r_0, environmental carrying capacity K, and Allee effect intensity (A) are intrinsic influencing parameters of the cells themselves. Meanwhile, the heat production rate of the photosensitizer via photothermal effect α and the increased ROS production rate of the photosensitizer under surface plasmon enhancement β are intrinsic performance parameters of the probe itself. The initial concentration of photosensitizer C_0 and the relative lag time of photodynamic therapy φ are man-controlled factors during probe-based therapy. The following analysis mainly focuses on exploring the model of these two categories of key parameters, namely the probe's intrinsic parameters and the man-controlled factors.

Table S1 Parameter Names, Meanings, and Baseline Values

Parameters	The meanings of parameters	Baseline values
X(t)	Tumor cell number density	Variable
H(t)	Actual photothermal therapy intensity	Variable
B(t)	Actual photodynamic therapy intensity	Variable
C(t)	Photosensitizer concentration	Variable
r_0	Intrinsic growth rate of tumor cells	1
K	Maximum environmental carrying capacity	10
τ	Time delay of tumor cell cycle	1
μ_H	Tumor cell killing rate of photothermal therapy	0.5
μ_B	Tumor cell killing rate of photodynamic therapy	1
α	Heat production rate of photosensitizer from photothermal effect	0.05
Q	Heat production of gold nanorods	3
λ_H	Non-therapeutic loss rate of heat	1
λ_B	Non-therapeutic loss rate of ROS	0.5
λ_C	Photodegradation rate of photosensitizer	0.5
β_C	ROS production rate of photosensitizer	0.1
β	Increased ROS production rate of photosensitizer under surface plasmon enhancement	0.05
γ_A	Heat loss rate during therapeutic process	0.5
γ_B	Reactive oxygen species loss rate during therapeutic process	0.3
φ	Relative time delay of PDT compared to PTT	0.5
C_0	Initial concentration of photosensitizer	20
A	Allee effect intensity	0.12*K

In recent years, improving the diagnostic sensitivity for early-stage tumors and the therapeutic efficacy for advanced-stage tumors has increasingly become two key priorities in the fight against tumor diseases. This model is a system of nonlinear ordinary differential equations. An analytical solution is difficult to derive for such a system of equations. Therefore, this study employs the fourth-order Runge-Kutta (RK4) method to compute its numerical solution.

Runge-Kutta Methods are a family of numerical algorithms developed around the 1900s by two mathematicians, Carl Runge and Martin Wilhelm Kutta. This type of algorithm is a one-step algorithm and has relatively high accuracy. This method is based on Taylor expansion and evolved from the simple Euler method. The most widely used Runge-Kutta method is the most classic fourth-order variant, which exhibits a truncation error of order $\mathrm{o}(\mathrm{h}^5)$. For the given initial conditions:

$$y' = f(x, y),\ y(x_0) = y_0 \quad (10)$$

The iterative formulation of RK4 is as follows:

$$y_{n+1} = y_n + \frac{h}{6}(k_1 + 2k_2 + 2k_3 + k_4) \quad (11)$$

Therein,

$$k_1 = f(x_n, y_n) \quad (12)$$

$$k_2 = f\left(x_n + \frac{h}{2}, y_n + \frac{h}{2}k_1\right) \quad (13)$$

$$k_3 = f\left(x_n + \frac{h}{2}, y_n + \frac{h}{2}k_2\right) \quad (14)$$

$$k_4 = f(x_n + h, y_n + hk_3) \quad (15)$$

During the solution process of this study, Mathematica and MATLAB were employed as computational tools. For the solution, the time range for t was set to 0–5,

the solution step size was 0.1, and the precision was 10^{-5}.

Section 5: Statistical Analysis

All data were presented as the mean ± standard deviation (SD), with the nature of replicates explicitly distinguished for all experiments. For *in vivo* animal experiments such as tumor growth monitoring, multimodal imaging and therapeutic efficacy evaluation, five BALB/c white mice per group were used as independent biological replicates. For *in vitro* cellular experiments including cell viability assay, reactive oxygen species (ROS) detection and immunogenic cell death (ICD) marker analysis, three independent cell culture batches per group were set as biological replicates to reflect the true biological variability among different cell populations. For material characterization experiments such as transmission electron microscopy (TEM), X-ray photoelectron spectroscopy (XPS), UV-Vis-NIR absorption spectroscopy and fluorescence spectroscopy, each sample was tested in triplicate as technical replicates to reduce random errors during instrumental detection.

The number of biological replicates (n value) is clearly indicated in all figure captions. SD is used to characterize data dispersion instead of Standard Error of the Mean (SEM) in this study because SD reflects the absolute degree of variation of raw data within each experimental group, enabling researchers to intuitively understand the distribution range of biological sample data, while SEM primarily reflects the precision of the sample mean relative to the population mean estimation and is more suitable for comparing confidence intervals of means between groups. Since this study focuses on the variability characteristics of data within each experimental group and the direct

comparison of data distributions between groups, SD is a more appropriate descriptive statistic for the research purposes. One-way analysis of variance (ANOVA) is only used for statistical comparison of biological replicate data, which is consistent with the requirements for statistical testing of independent biological sample data, and technical replicate data are only used to calculate the mean and SD with no ANOVA performed to avoid statistical bias caused by pseudo-replication.

Statistical analysis and quantitative graph generation were performed using GraphPad Prism 8 and Origin 2024 software. A p-value less than 0.05 was considered as statistically significant (*$p < 0.05$, **$p < 0.01$, and ***$p < 0.001$; ns: not significant). All data of fluorescence-based experiments were quantitatively calculated using ImageJ software.

Supplementary References

S1 Shao Y. Z., Tian X. M., Hu W. Y., Zhang Y. Y., Liu H., He H. Q., Shen Y. Y., Xie F. K., Li L. The properties of Gd_2O_3-assembled silica nanocomposite targeted nanoprobes and their application in MRI. *Biomaterials* **33**, 6438-6446 (2012).

S2 Li S., Liu H., Li L., Luo N. Q., Cao R. H., Chen D. H., Shao Y. Z. Mesoporous silica nanoparticles encapsulating Gd2O3 as a highly efficient magnetic resonance imaging contrast agent. *Appl. Phys. Lett.* **98**, 093704 (2011).

S3 Perdew J. P., Burke K., Ernzerhof M. Generalized gradient approximation made simple. *Phys. Rev. Lett*. **77**, 3865-3868 (1996).

S4 Johnson P. B., and Christy R. W., Optical constants of the noble metals. *Phys. Rev. B* **6**, 4370–4379 (1972).

S5 Palik E. D., and Ghosh G., Handbook of optical constants of solids. *Academic press*. **3**, Manhattan, NY, USA (1998).

S6 Bannwarth, C., Caldeweyher, E., Ehlert, S., Hansen, A., Pracht, P., Seibert, J., ... & Grimme, S. Extended tight - binding quantum chemistry methods. *WIRES. COMPUT. MOL. SCI.* **11**, e1493 (2021).

S7 Bannwarth, C., Ehlert, S., & Grimme, S. GFN2-xTB—An accurate and broadly parametrized self-consistent tight-binding quantum chemical method with multipole electrostatics and density-dependent dispersion contributions. *Chem. Theory Comput*. **15**, 1652-1671 (2019).

S8 Grimme, S., Ehrlich, S., & Goerigk, L. Effect of the damping function in dispersion corrected density functional theory. *J. Comput. Chem*. **32**, 1456-1465 (2011).

S9 Porto, C. M., Santana, L. C., & Morgon, N. H. Theoretical investigation of the cooperative effect of solvent: a case study. *Phys. Chem. Chem. Phys*. **24**, 14603-14615 (2022).

S10 Goerigk, L., & Grimme, S. Efficient and Accurate Double-Hybrid-Meta-GGA Density Functionals Evaluation with the Extended GMTKN30 Database for General Main Group Thermochemistry, Kinetics, and Noncovalent Interactions. *J. Chem. Theory Comput*. **7**, 291-309 (2011).

S11 Weigend, F., & Ahlrichs, R. Balanced basis sets of split valence, triple zeta valence and quadruple zeta valence quality for H to Rn: Design and assessment of accuracy. *Phys. Chem. Chem. Phys*. **7**, 3297-3305 (2025).

S12 Neese, F., Wennmohs, F., Becker, U., & Riplinger, C. The ORCA quantum chemistry program package. *J. Chem. Phys*. **152** (2020).

S13 Lu, T. Molclus program, Version 1.9.9.9, http://www.keinsci.com /research/molclus.html (accessed 12, August, 2022).

S14 Yanai, T., Tew, D. P., & Handy, N. C. A new hybrid exchange–correlation functional using the Coulomb-attenuating method (CAM-B3LYP). *Chem. Phys. Lett*. **393**, 51-57 (2004).

S15 Adamo, C., & Barone, V. Toward reliable density functional methods without adjustable parameters: The PBE0 model. *J. Chem. Phys*. **110**, 6158-6170 (1999).

S16 Andrae, D., Häußermann, U., Dolg, M., Stoll, H., & Preuss, H. Energy-adjusted ab initio pseudopotentials for the second and third row transition elements. *Theor. Chim. Acta* **77**, 123-141 (1990).

S17 Lu, T. and Chen, F. "Multiwfn: A multifunctional wavefunction analyzer," *J. Comput. Chem.* **33**, 580-592 (2012).

S18 Humphrey, W., Dalke, A., K. Schulten, K. "VMD: visual molecular dynamics," *J. Mol. Graph.* **14**, 33-38 (1996).

S19 Nakajima, T. and Hirao, K. "The Douglas–Kroll–Hess Approach," *Chem. Rev.* **112**, 385-402 (2012).

S20 Aravena, D., Neese, F., Pantazis, D. A. "Improved Segmented All-Electron Relativistically Contracted Basis Sets for the Lanthanides," *J. Chem. Theory Comput.* **12**, 1148-1156 (2016).

S21 Helmich-Paris, B., de Souza, B., Neese, F., Izsák, R. "An improved chain of spheres for exchange algorithm," *J. Chem. Phys.* **155**, 104109 (2021).

S22 Byrne, Helen M. Modelling avascular tumour growth. *Cancer Modelling and Simulation*. 2003.

S23 Zheng X., Xing D., Zhou F., et al. Indocyanine Green-Containing Nanostructure as Near Infrared Dual-Functional Targeting Probes for Optical Imaging and Photothermal Therapy. *Mol. Pharm*. **8**, 447-456 (2011).

S24 Wang S., Huang P., Nie L., et al. Single continuous wave laser induced photo dynamic/plasmonic photothermal therapy using photosensitizer-functionalized gold nanostars. *Adv. Mater*. **25**, 3055-3061 (2013).

S25 Li Y., Wen T., Zhao R., et al. Localized Electric Field of Plasmonic Nanoplatform Enhanced Photodynamic Tumor Therapy. *ACS Nano*, **8**, 11529-11542 (2014).

S26 Saxena V., Sadoqi M., Shao J. Degradation kinetics of indocyanine green in aqueous solution. *J. Pharm. Sci*, **92**, 2090-2097 (2003).

S27 Zhong W. R, Shao Y Z, He Z. H. Pure multiplicative stochastic resonance of a theoretical anti-tumor model with seasonal modulability. *Phys. Rev. E* **73**, 060902 (2006).

S28 Zhong W. R., Shao Y. Z., He Z. H. Spatiotemporal Fluctuation Induced Transition in a Tumor Model with Immune Surveillance. *Phys. Rev. E* **74**, 011916 (2006).

Supplementary Figures

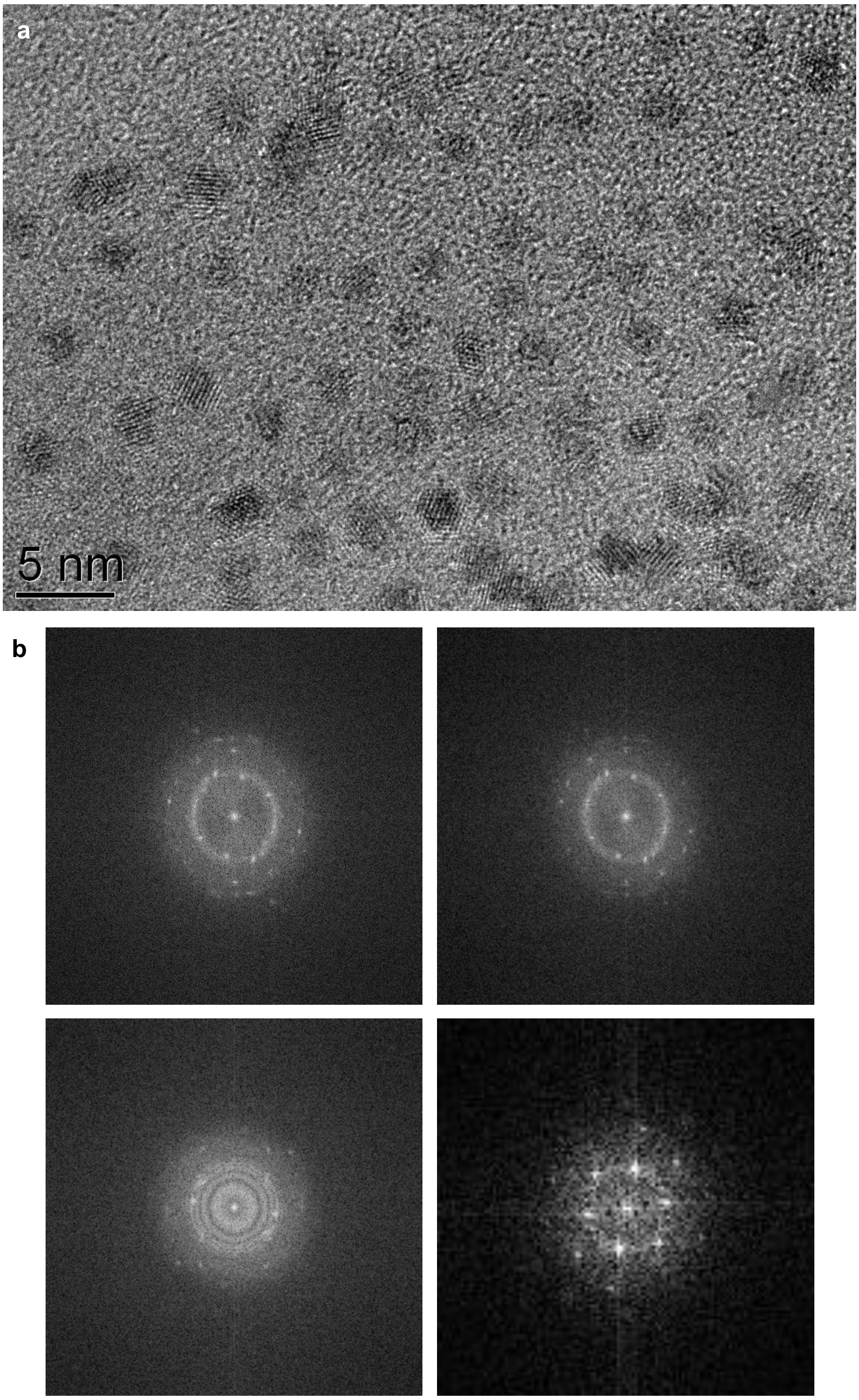

Fig. S1. a Transmission electron microscopy image of rare-earth-doped gadolinium oxide nanocrystals (ReNCs) and **b** fast Fourier transform images of different single rare-earth particles.

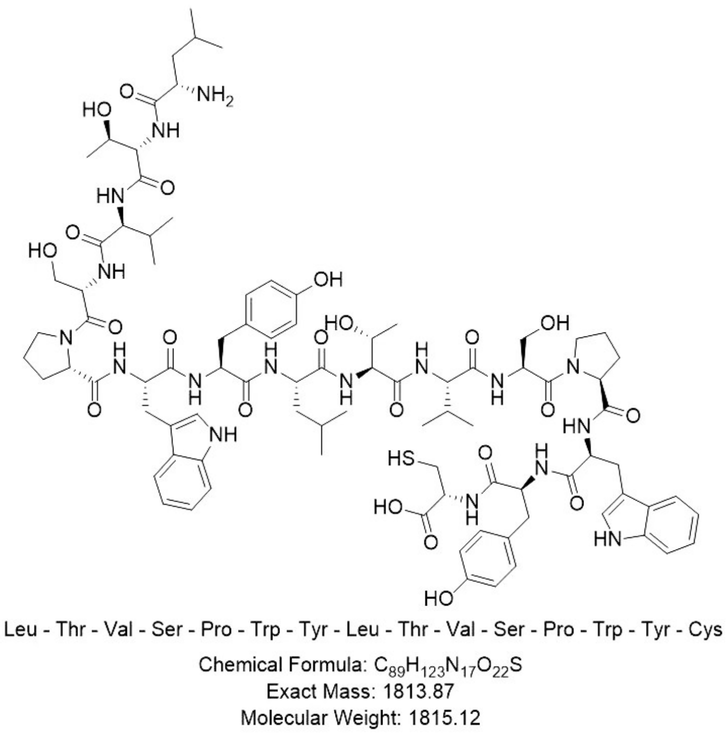

Fig. S2. Molecular structures of the nasopharyngeal carcinoma (NPC)-targeting peptides (LTVSPWYL TVSPWYC) abbreviated as NTP.

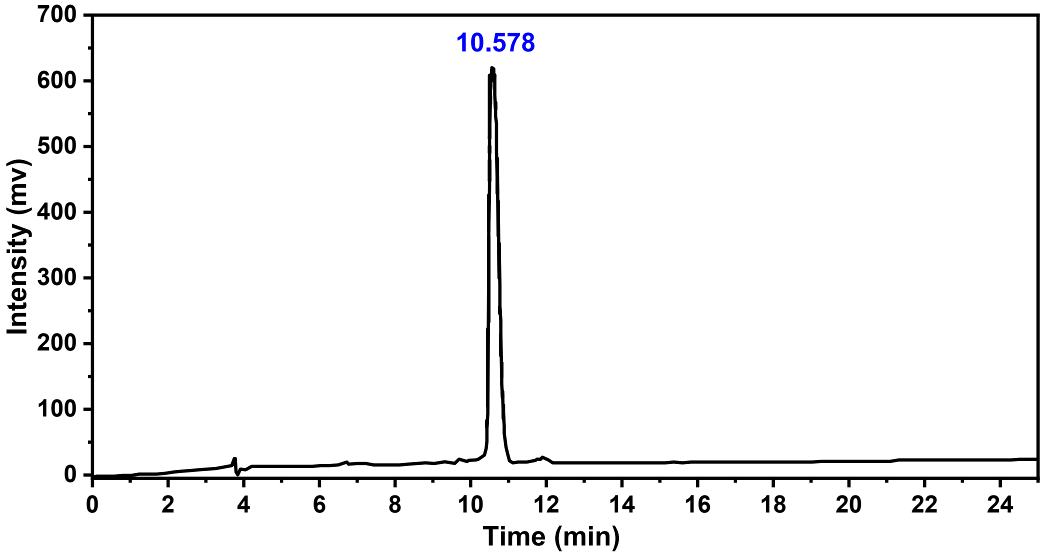

Fig. S3. The high-performance liquid chromatography (HPLC) of nasopharyngeal carcinoma-targeting peptides.

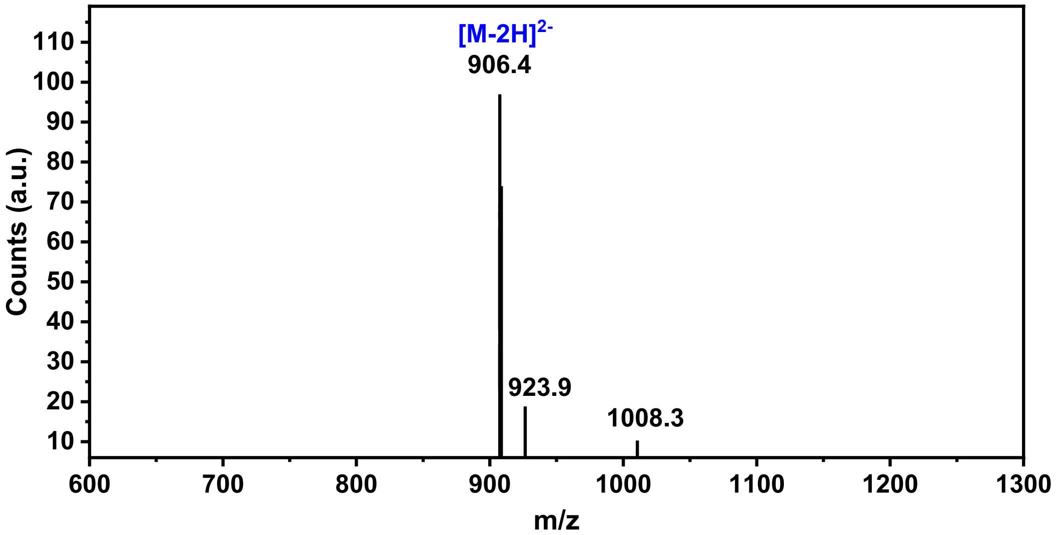

Fig. S4. The electrospray ionization mass spectrometry (ESI-MS, Agilent-6125B, USA) of nasopharyngeal carcinoma-targeting peptides (LTVSPWYLTVSPWYC).

a
b
c

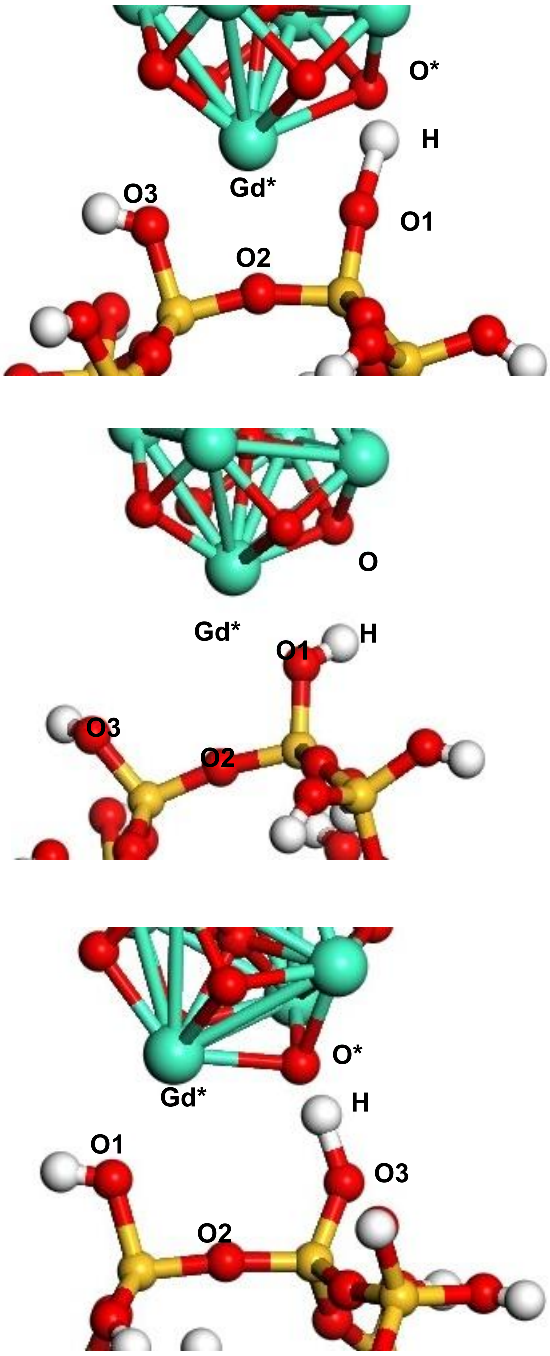

Fig. S5. Three orientations of the cluster were modeled, which were marked as Gd-O (Fig. S5a), Gd-OH (Fig. S5b) and O-H (Fig. S5c) respectively. As the name indicates, Gd-O means a gadolinium atom in Gd_6O_9 cluster approaching the oxygen atom in SiO_2, Gd-OH means a gadolinium atom in Gd_6O_9 cluster approaching the oxygen atom in hydroxyl on the surface of SiO_2, and O-H means an oxygen atom in Gd_6O_9 cluster

approaching the hydrogen atom in hydroxyl on the surface of SiO_2. Colors for the atoms: cyan-gadolinium, red-oxygen, white-hydrogen, yellow-silicon.

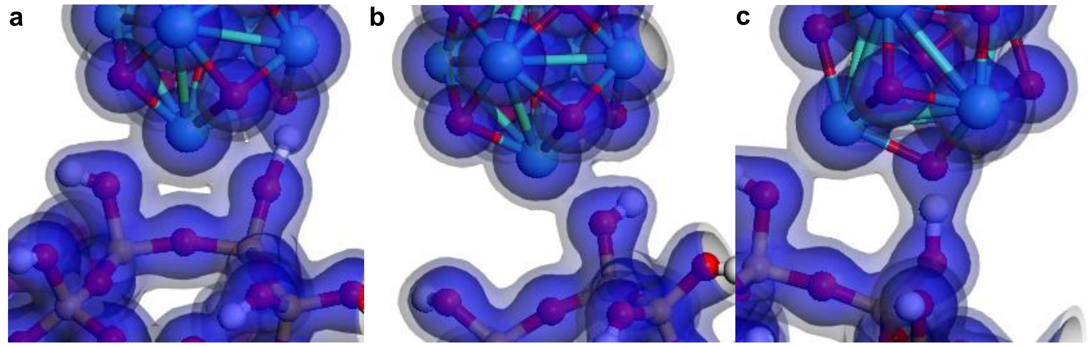

Fig. S6. a-c Electron density distributions with the same isosurface value for three orientations of the cluster as modeled and marked as Gd-O (Fig. S5a), Gd-OH (Fig. S5b) and O-H (Fig. S5c) in Fig. S5, respectively. The isosurfaces of electron density reveal that partial of the electron clouds of Gd_6O_9 cluster are connected to electron clouds of silica, indicating newly formed bonds.

To evaluate the strength of bonds, the adsorption energies were calculated by Eq. (16).

$$E_{adsorption} = E_{absorbed} - E_{isolated} \tag{16}$$

The calculated energies of three models are listed in Table S2. According to the expectation for the models, the total energies decrease, showing that the Gd_6O_9 clusters interact with the silica and form bonds. To make a comprehensive study on the interactions between Gd_6O_9 clusters and the silica, Mulliken charges were calculated and listed in Table S3, Table S4 and Table S5 to specify the interactions, the corresponding atoms were marked in Fig. S5.

Table S2. Energy differences

**Model**	$E_{Gd_6O_9}$/ Ha	E_{silica}/ Ha	$E_{absorbed}$/ Ha	$E_{adsorption}$ / Ha	$E_{adsorption}$ /eV
**Gd-O**	-65621.514422	-651764.912308	-717386.958336	-0.531606	-14.4657
**Gd-OH**	-65621.514422	-651764.967045	-717386.924652	-0.443185	-12.0597
**O-H**	-65621.514422	-651764.930694	-717386.94855	-0.497188	-13.5292

In the case of Gd-O, both of the gadolinium atom (Gd*) and the hydrogen atom (H) lost charge obviously after interaction, while the oxygen atoms (O1 and O3) have more charge. It is because of the interaction between gadolinium (Gd*) and oxygen (O1 and O3), which induces charge transfer from gadolinium and hydrogen to oxygen. As a result, there are three new bonds formed, Gd*-O1, Gd*-O3 and O*-H. Although O*-H bond was formed, the charge addition of O* is small. The calculated total charge on O* is larger than on O1 and on O3, and the electrons are denser, indicating a larger force is needed to absorb electrons. Furthermore, the expected Gd*-O2 interaction is so weak that cannot be observed. The Gd_6O_9 cluster is large compared to the adjacent vacancy between two hydroxyls, so the cluster is blocked away from the inner oxygen in SiO_2 network and form Gd*-O1, Gd*-O3 and O*-H bonds prior to Gd*-O2.

Table S3. Charge variation in Gd-O (Rows in shade stand for atoms in isolated particles, and the mark '*' represents atoms in Gd_6O_9 cluster, the same for the followings)

	s	p	d	f	total	difference
**Gd*(before)**	0.190	0.091	0.996	7.459	8.736	-
**Gd*(after)**	0.140	0.166	0.800	7.517	8.623	-0.113
**O***	1.943	4.860	0.017	-	6.820	-
**O***	1.885	4.950	0.015	-	6.850	+0.030
**H**	0.942	-	-	-	0.942	-
**H**	0.536	-	-	-	0.536	-0.406
**O1**	1.920	4.675	0.013	-	6.608	-
**O1**	1.907	4.865	0.014	-	6.786	+0.182
**O2**	1.861	4.954	0.011	-	6.826	-
**O2**	1.826	4.988	0.011	-	6.825	-0.001
**O3**	1.876	4.837	0.021	-	6.734	-
**O3**	1.837	4.986	0.019	-	6.842	+0.108

The situation is similar in the case of Gd-OH. The charges in gadolinium atom (Gd*) and the hydrogen atom (H) decreased, while the charge in oxygen atom (O1) increased. The interactions between Gd* and O1 and between O1 and H reduce the charge of H. The charge transfer suggests that the Gd*-O1 bond is formed.

The model O-H behaves similarly. There are charge transfers from Gd* to O1 and from H to O*. Therefore, Gd*-O1 bond and O*-H bond were formed. Due to the long distance, the interaction between Gd* and O1 is weaker than the previous two models, the difference of charge is accordingly smaller.

To conclude the result of the simulation of the three models, there are strong

interactions between Gd_6O_9 clusters and silica which coincide with the previous works. To be specific, there are two main interactions as follows. The first is between gadolinium atoms in Gd_6O_9 clusters and oxygen atoms in hydroxyl on the surface of silica, which is consistent with the result of XPS. And the second is between oxygen atoms in Gd_6O_9 clusters and hydrogen atoms in hydroxyl on the surface of silica. As a result, the Gd_6O_9 clusters can be firmly absorbed on the surface of silica.

Table S4. Charge variation in Gd-OH

	s	p	d	f	total	difference
**Gd*(before)**	0.190	0.091	0.996	7.459	8.736	-
**Gd*(after)**	0.140	0.166	0.800	7.517	8.623	-0.113
**O***	1.943	4.860	0.017	-	6.820	-
**O***	1.885	4.950	0.015	-	6.850	+0.030
**H**	0.942	-	-	-	0.942	-
**H**	0.536	-	-	-	0.536	-0.406
**O1**	1.920	4.675	0.013	-	6.608	-
**O1**	1.907	4.865	0.014	-	6.786	+0.178
**O2**	1.849	5.001	0.011	-	6.861	-
**O2**	1.826	4.988	0.011	-	6.825	-0.026
**O3**	1.863	4.874	0.019	-	6.756	
**O3**	1.844	4.889	0.020	-	6.753	-0.003

Table S5. Charge variation in O-H

	s	p	d	f	total	difference
**Gd*(before)**	0.190	0.091	0.996	7.459	8.736	-
**Gd*(after)**	0.163	0.176	0.848	7.523	8.710	-0.026
**O***	1.943	4.860	0.017	-	6.820	-
**O***	1.903	5.015	0.013	-	6.931	+0.111
**H’**	0.817	-	-	-	0.817	-
**H’**	0.501	-	-	-	0.501	-0.316
**O1**	1.893	4.810	0.022	-	6.725	-
**O1**	1.853	4.956	0.019	-	6.828	+0.103
**O2**	1.846	5.002	0.010	-	6.858	-
**O2**	1.819	5.001	0.011	-	6.831	-0.027
**O3**	1.896	4.769	0.014	-	6.679	-
**O3**	1.894	4.838	0.015	-	6.747	+0.068

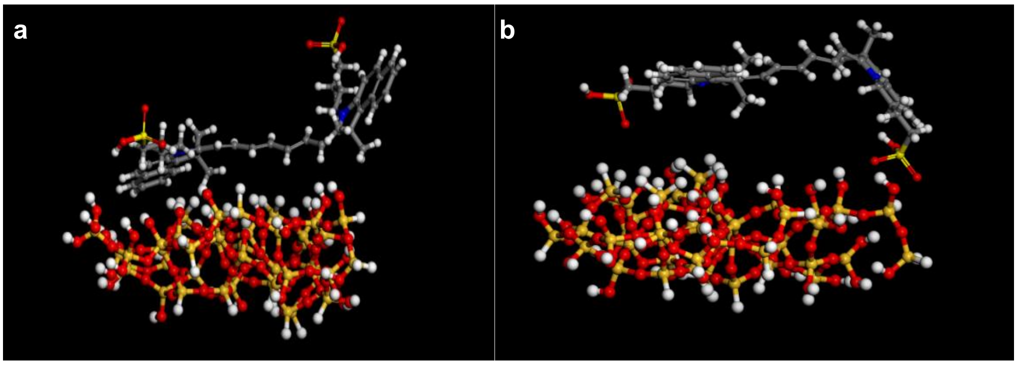

Fig. S7. On the basis of the successful structural optimization of multiple components, calculations on the interactions between the silica surface (short) and ICG were performed, and the results obtained are as follows. Two configurations of ICG-SiO_2 were designed: one with the sulfonic acid group far from the silica cluster (denoted as Configuration A), as shown in Fig. S7a; the other with the sulfonic acid group close to the silica cluster (denoted as Configuration B). The interaction diagram of Configuration A is presented in Fig. S7b.

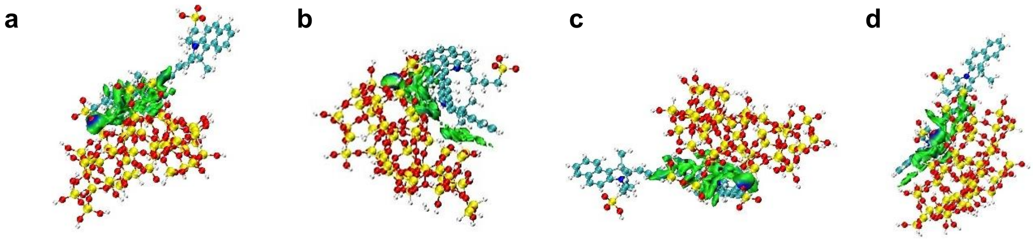

Fig. S8. Visualization results of van der Waals interactions for configuration A in Fig. S7. Fig. S8a, b, c, and d are images of the same model from different angles. The green planes represent the primary van der Waals interactions between the two molecular segments: for this type of adsorption, a greater number of green planes indicates a more stable adsorption state, and such adsorption generally exerts little influence on the chemical properties of the functional groups.

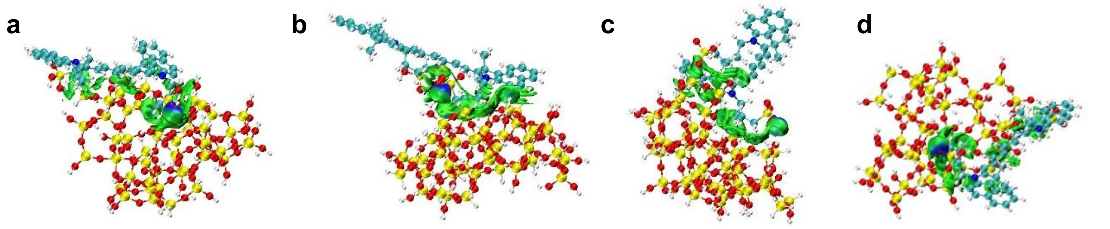

Fig. S9. Visualization results of van der Waals interactions for configuration B in Fig. S7. Fig. S9a, b, c, and d are images of the same model from different angles. The green planes represent the primary interaction, van der Waals interactions, between the two molecular segments (i.e., the silica surface and ICG). It was observed that the range and nature of the interactions involving the sulfonic acid groups in Configurations A and B remained essentially unchanged, indicating that the distance of the sulfonic acid groups from the silica surface (whether distant or close) exerted no significant impact on these interactions.

In practical experiments, it is often the case that the scale of the silica surface is larger than that of attached components such as photosensitizers. Therefore, we considered constructing a longer silica surface; after conducting structural optimization on it, we performed calculations together with ICG. The results obtained are as follows:

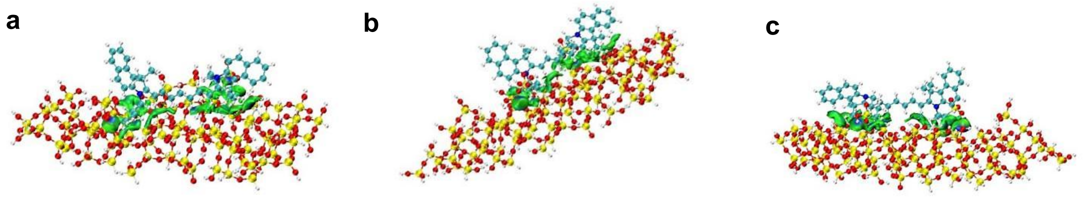

Fig. S10. Visualization results of van der Waals interactions between the long silica surface and ICG. Figs. S10a, b, c, and d are images of the same model from different angles, showing more interfaces of van der Waals interactions. In theory, as long as there are sufficient such weak interactions, the ICG component can be firmly adsorbed.

Computational analysis indicates that a silica surface with a larger scale can adsorb ICG more firmly.

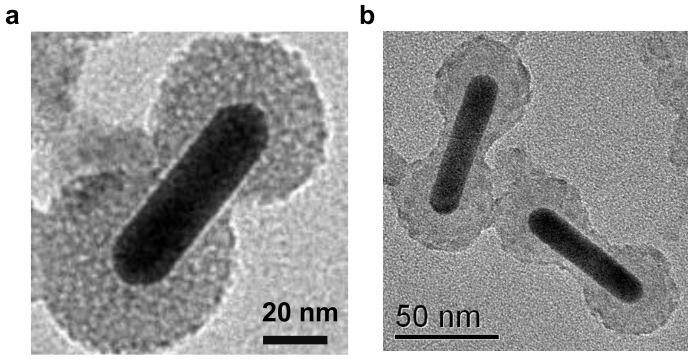

Fig. S11. Dumbbell-structured mesoporous silica ($mSiO_2$)-coated gold nanorods (AuNRs) **a** before and **b** after anchoring rare-earth-doped gadolinium oxide nanocrystals (ReNCs).

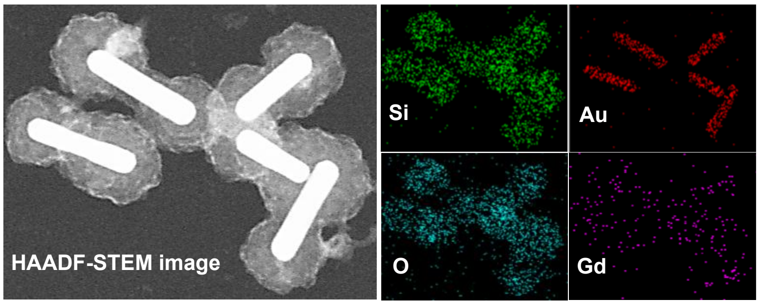

Fig. S12. High-angle annular dark-field scanning TEM (HAADF-STEM) image and the corresponding Si, Au, O and Gd EDX elemental mapping images of dumbbell-structured $mSiO_2$-coated AuNRs after anchoring ReNCs and ICG molecules.

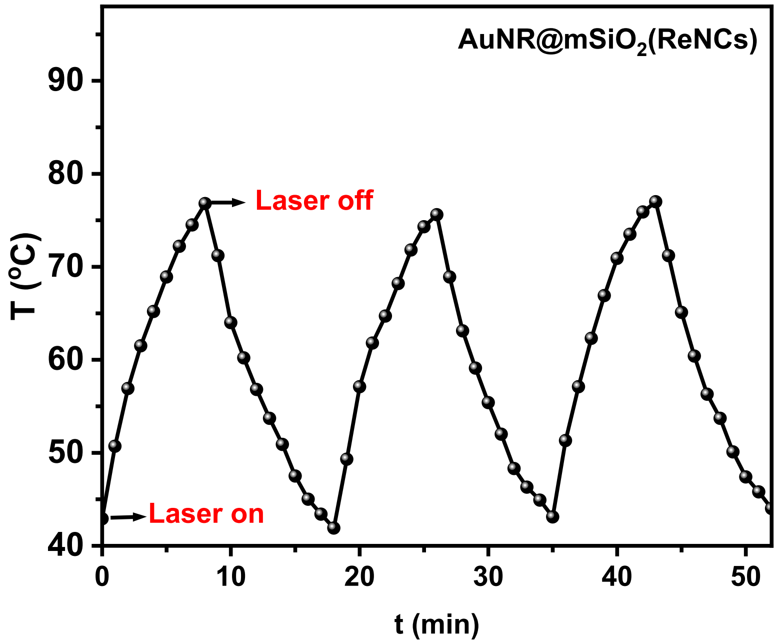

Fig. S13. Temperature variation of the $mSiO_2$-coated AuNRs after anchoring ReNCs during three photothermal heating cycles under 808 nm laser irradiation (0.5 W/cm²).

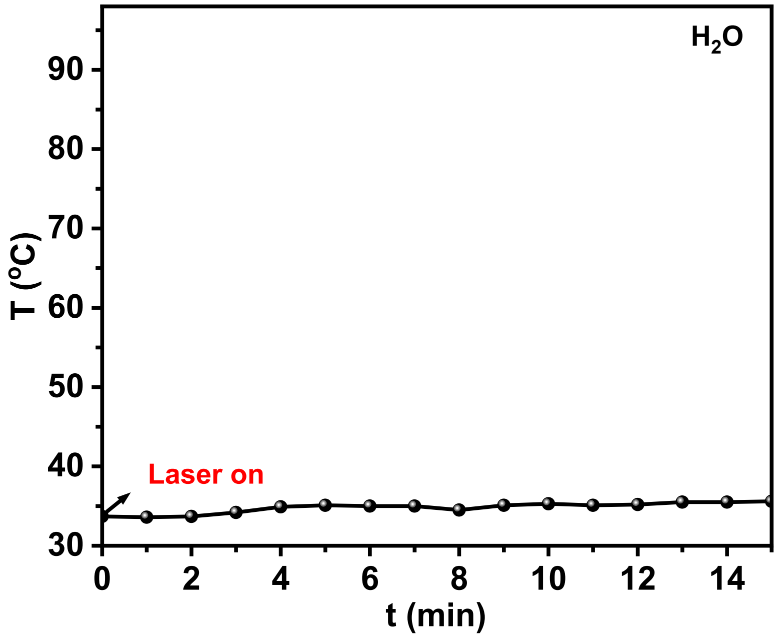

Fig. S14. Time-dependent temperature curve of the medium solution under 808 nm laser (0.5 W/cm²) irradiation.

Table S6. Variation of simulated structure models of the nanoprobes in FDTD algorithm.

	radius of $mSiO_2$	number of ICG/ ReNCs	peak of absorbance
**1**	27 nm	1:1	815 nm
**2**	26.25 nm	1:1.2	813 nm
**3**	25.5 nm	1:1	810 nm
**4**	24.75 nm	1:1.5	800 nm
**5**	24 nm	1:1.2	790 nm

The absorbance curve of model 4 is the nearest compared to that of nanoprobes in Fig.3a.

The following are the results of simulation of the other models (Fig.S15-S20).

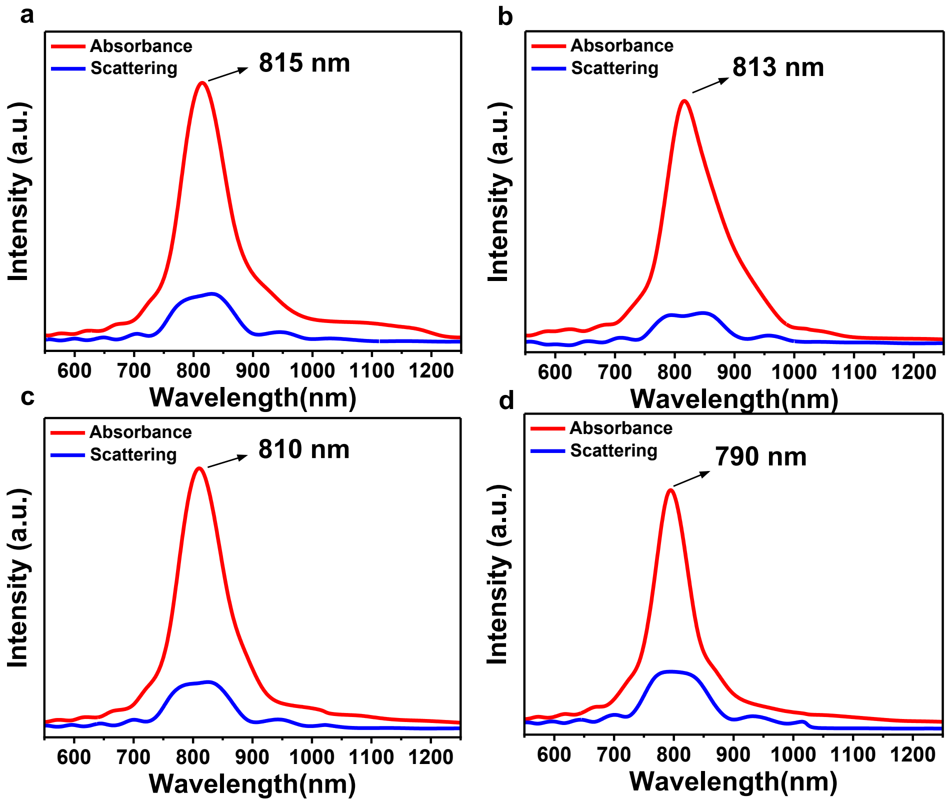

Fig. S15. NIR absorbance cross section simulated with FDTD algorithm. **a**, **b**, **c**, **d** corresponding to simulated structure models **1**, **2**, **3**, **5**, in Table S6, respectively.

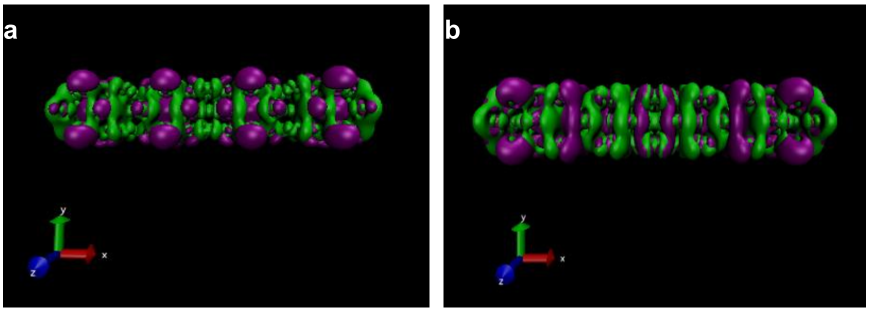

Fig. S16. Electron-hole transfer diagram at the highest absorption peak **a** and second-highest peak **b** of the gold nanorod. Calculations of electron-hole transfer at each absorption peak of the gold nanorod were conducted, and the electron-hole transfer at the highest peak and second-highest peak is presented in Fig. S16, where purple denotes electrons and green denotes holes. As shown in Fig. S16a, electrons and holes exhibit distinct morphological features characteristic of plasmonic charge transfer, leading to the conclusion that this peak corresponds to a plasmon resonance peak. In Fig. S16b, the distributions of electrons and holes are concomitant without obvious separation, indicating that this is not a plasmon resonance peak.

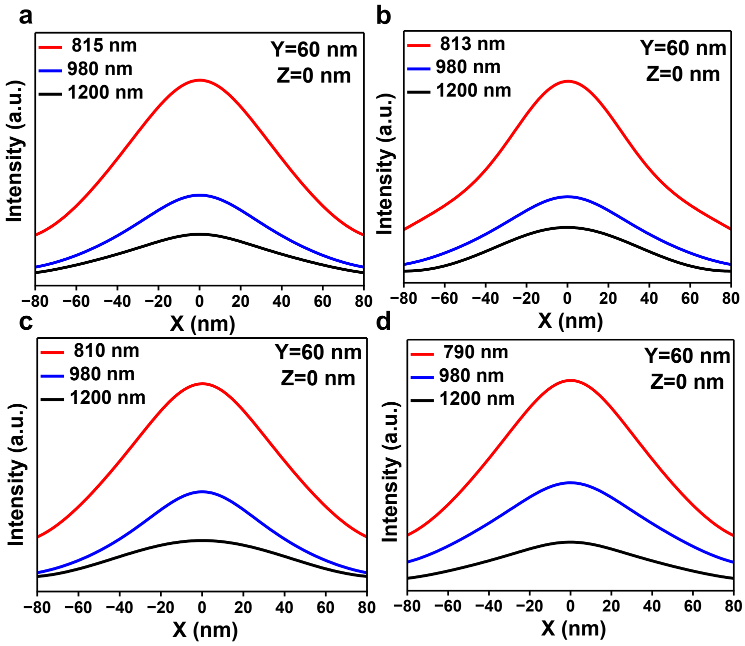

Fig. S17. Electric field enhancement curve along the x axis through different incident excitations. **a**, **b**, **c**, **d** corresponding to simulated structure models **1**, **2**, **3**, **5**, in Table S6, respectively.

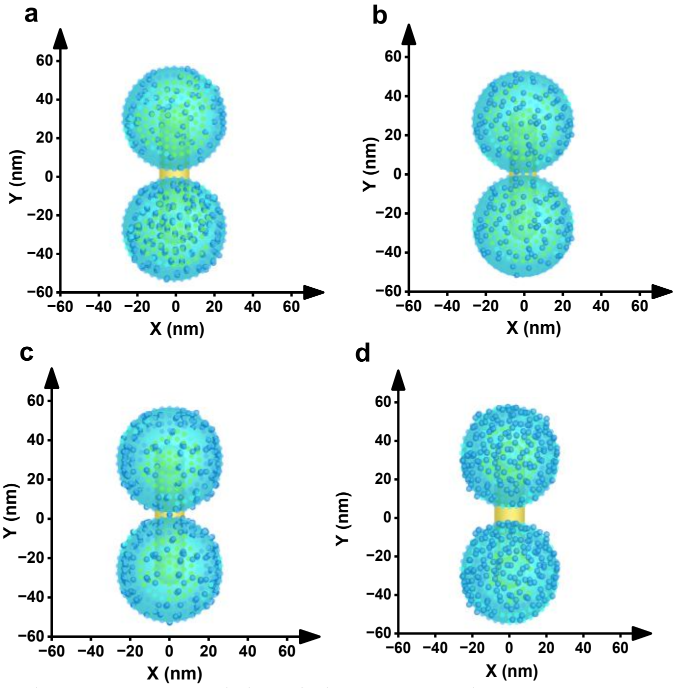

Fig. S18. Simulated structure models of the nanoprobes. **a**, **b**, **c**, **d** corresponding to simulated structure models **1**, **2**, **3**, **5**, in Table S6, respectively.

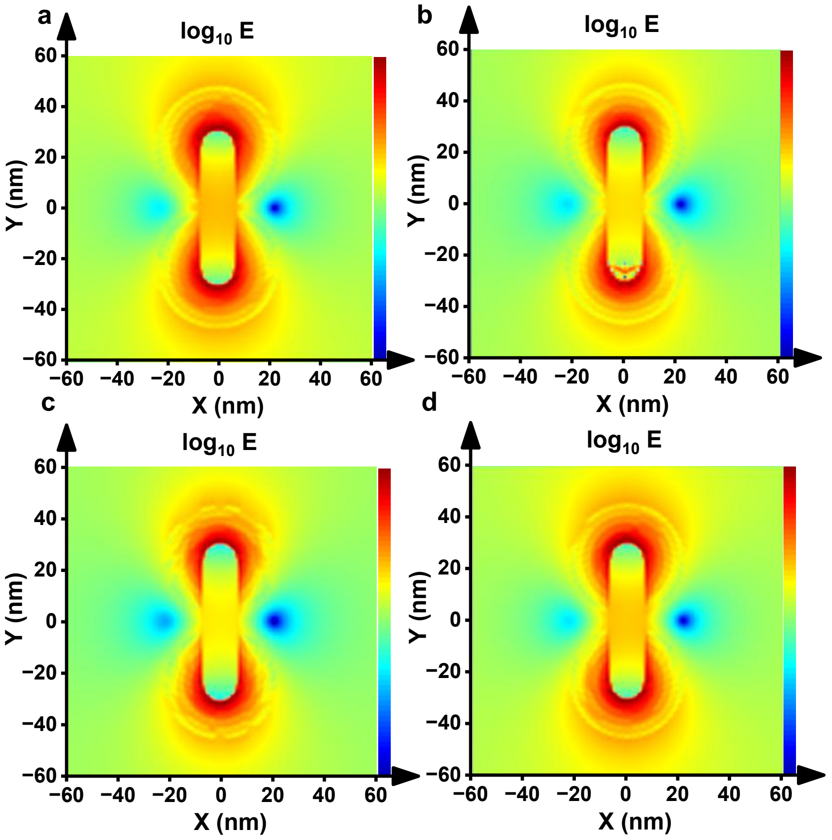

Fig. S19. The corresponding local electric field ***E*** distribution of the models. **a**, **b**, **c**, **d** corresponding to simulated structure models **1**, **2**, **3**, **5**, in Table S6, respectively.

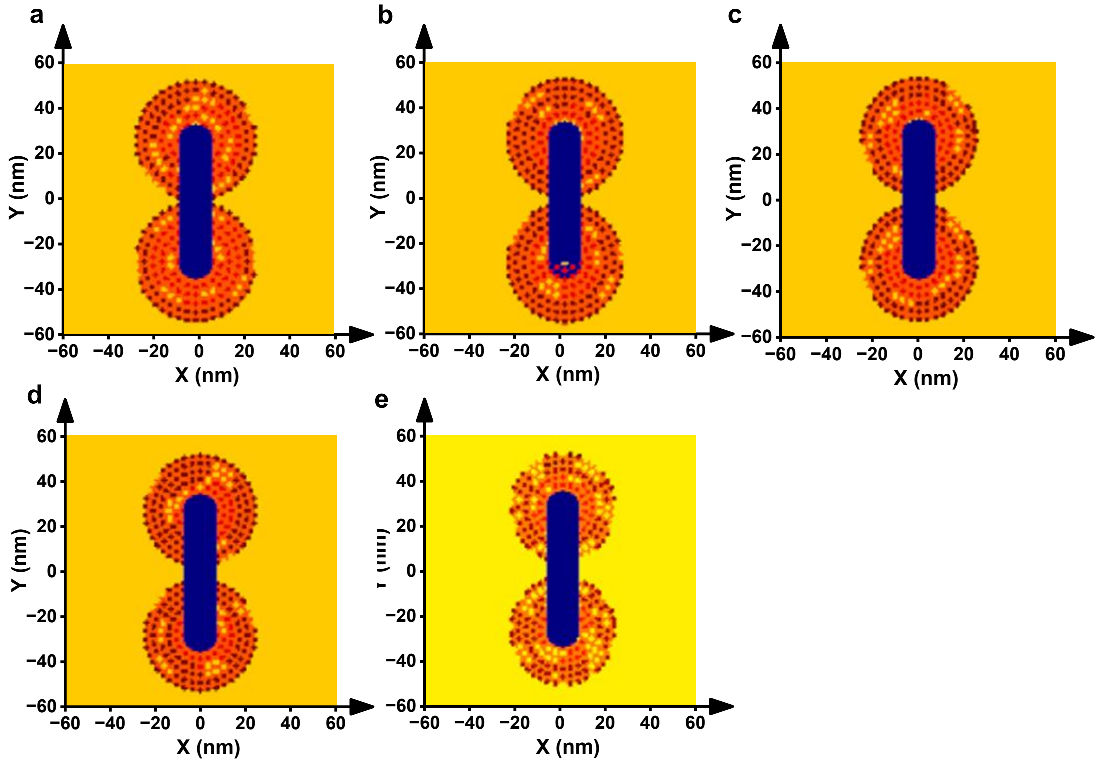

Fig. S20. The corresponding index distribution of the models. **a**, **b**, **c**, **d**, **e** corresponding to simulated structure models **1**, **2**, **3**, **4**, **5**, in Table S6, respectively.

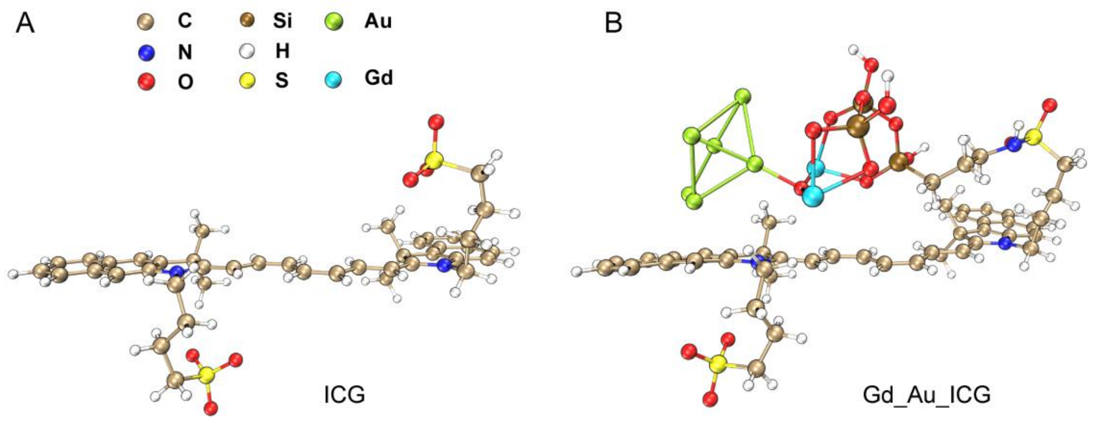

Figure S21. a Global energy minimum structure obtained in the conformational searching for ICG. **b** Constructed models for Gd_Au/ICG based on the most stable structure of ICG.

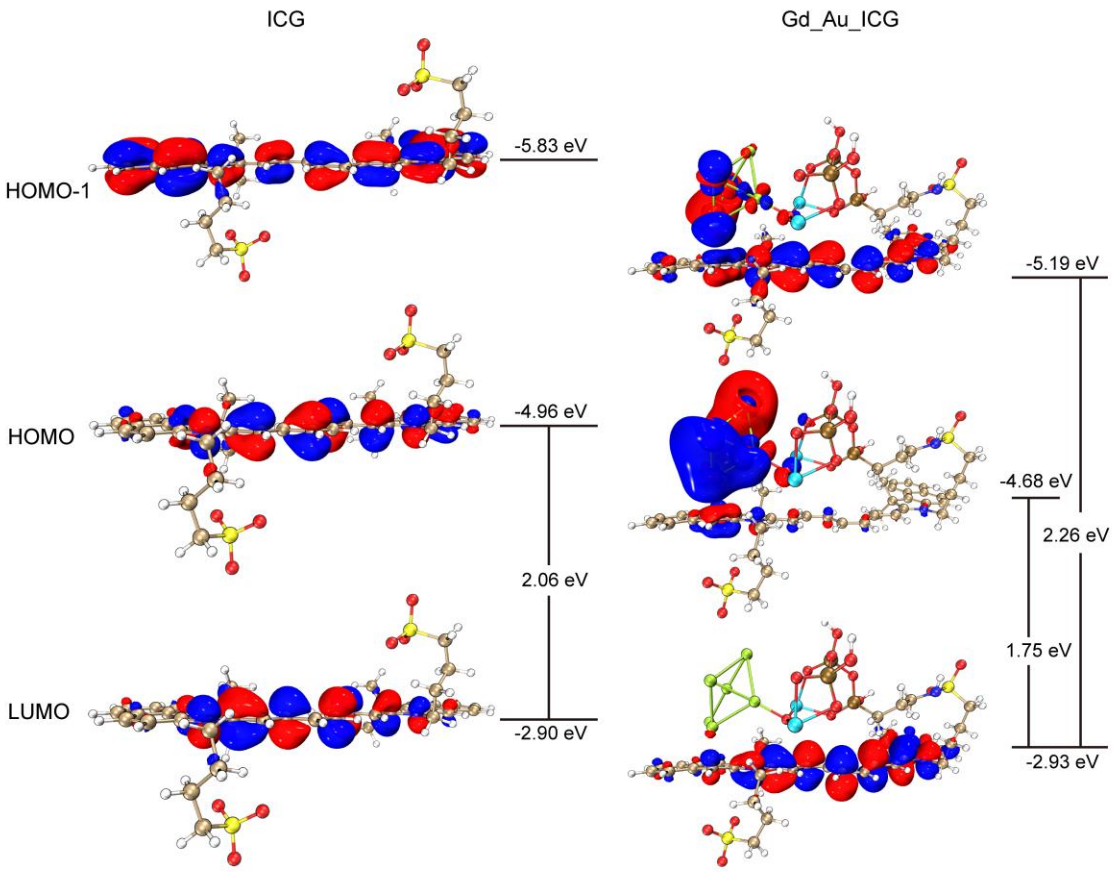

Figure S22. The orbital energy and distribution of HOMO-1, HOMO and LUMO for ICG and Gd_Au/ICG.

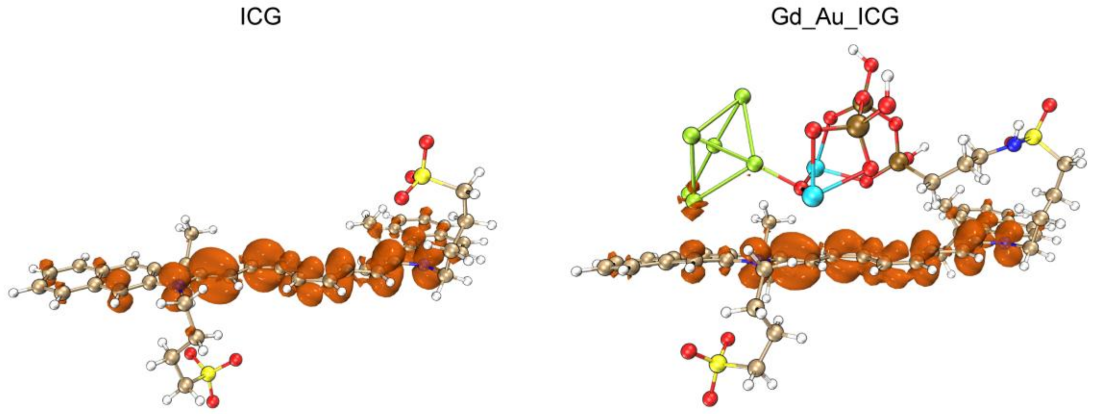

Figure S23. The real space overlap distributions of hole and electron for ICG and Gd_Au/ICG.

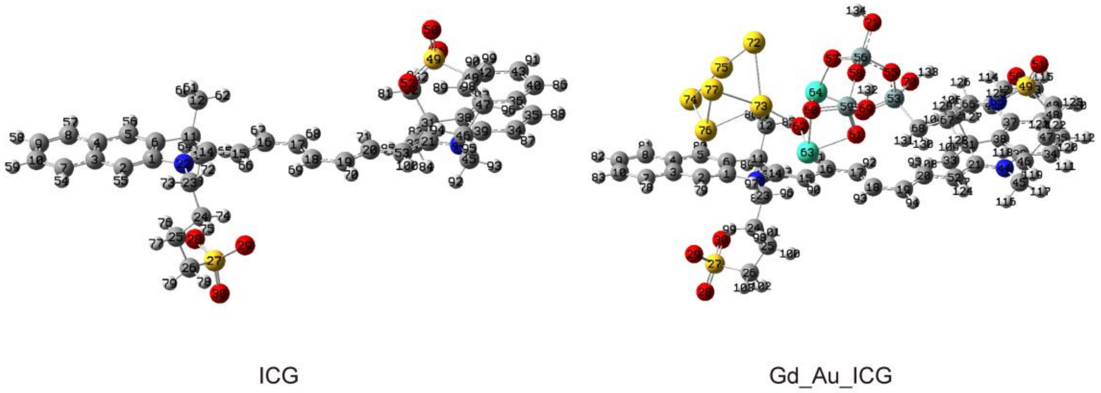

Figure S24. Geometry structures labelled with non-hydrogen atoms order for ICG and Gd_Au/ICG.

Table S7. The calculated matrix elements of the SOC operator $|\langle T_n|H_{SO}|S_1\rangle|$ (cm^{-1}) and corresponding energy gaps ΔE_{S1Tn} (eV).

ISC	ΔE_{S1Tn} (eV)		$\|\langle T_n\|H_{SO}\|S_1\rangle\|$ (cm^{-1})	
	ICG	Gd_Au/ICG	ICG	Gd_Au/ICG
$S_1 - T_1$	0.94	0.84	0.042	468.620
$S_1 - T_2$	-0.09	0.48	0	318.478
$S_1 - T_3$	—	0.02	—	63.939

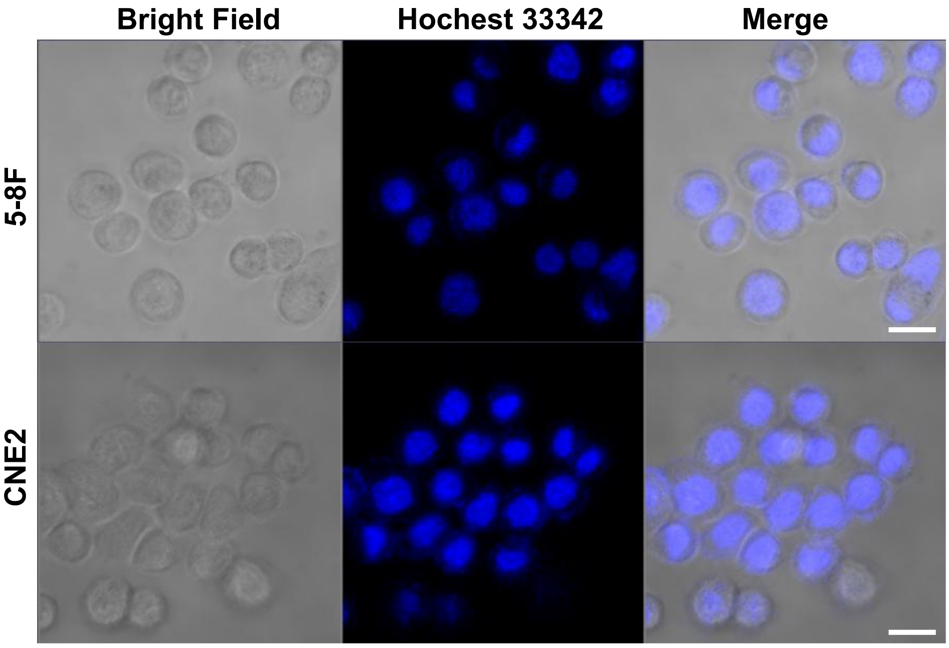

Fig. S25. CLSM images of 5-8F and CNE2 cancer cells incubated with no nanoprobes and stained with Hochest 33342. (scale bar: 20 μm).

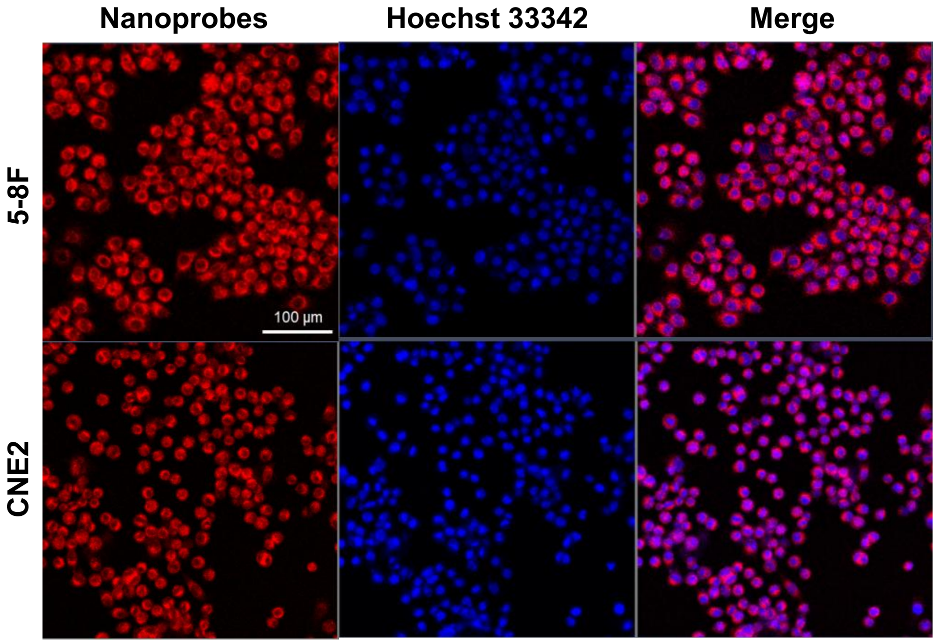

Fig. S26. CLSM images of 5-8F and CNE2 cancer cells incubated with nanoprobes for 4 h and stained with Hochest 33342. (scale bar: 100 μm).

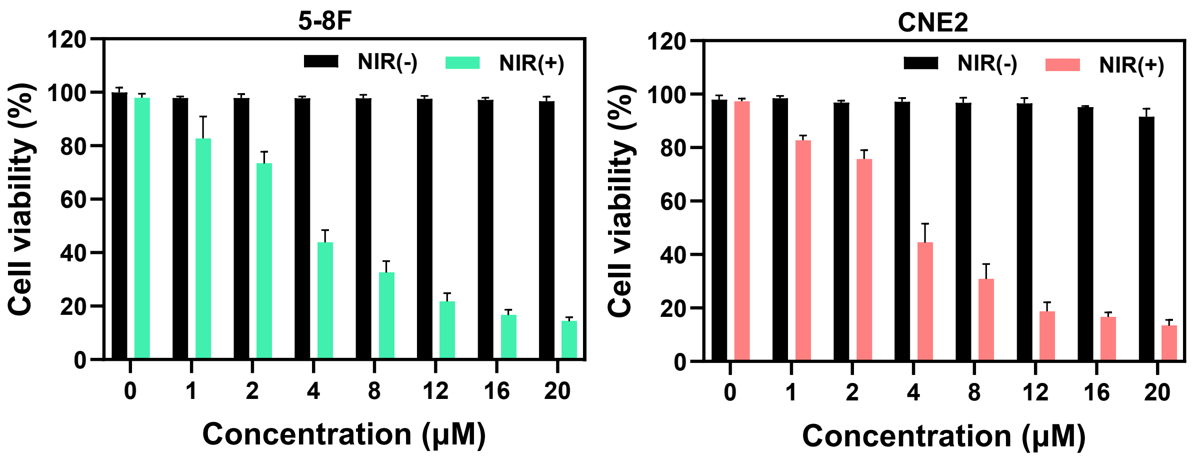

Fig. S27. Cell viabilities of 5-8F cells and CNE2 cells after incubation with different concentration of nanoprobes without and with 808 nm laser irradiation (0.5 W cm^{-2}). Data are presented as mean ± SD (n = 3/group).

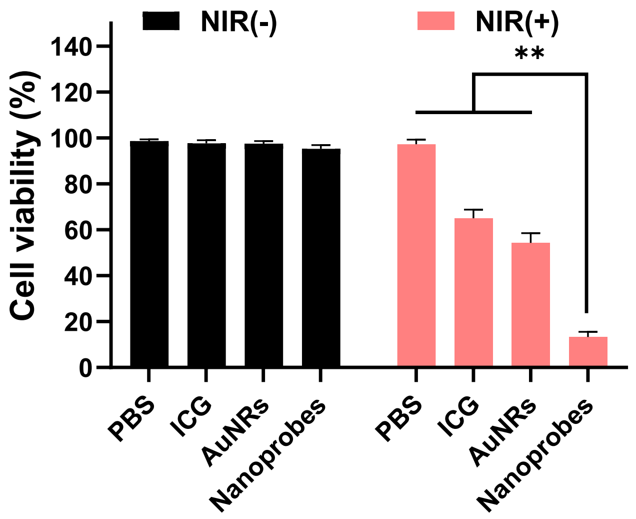

Fig. S28. Cell viability of CNE2 cells after different treatments. NIR(+) indicates the 808 nm laser is on while NIR(-) denotes the 808 nm laser is off. Data are presented as mean ± SD, n = 3, *$p < 0.05$, **$p < 0.01$, ***$p < 0.001$.

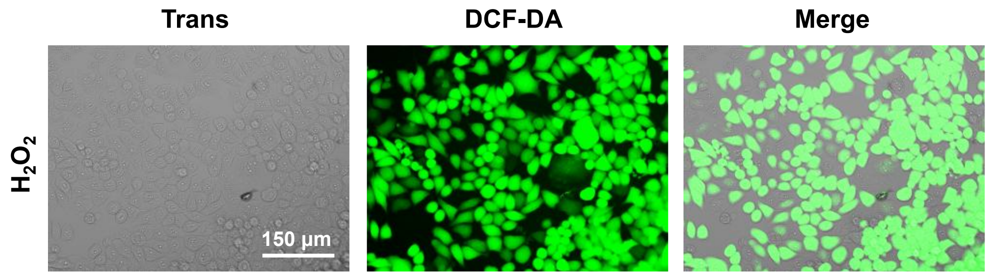

Fig. S29. Fluorescence images of 5-8F cells stained with DCFH-DA after treated with H_2O_2 as positive control group.

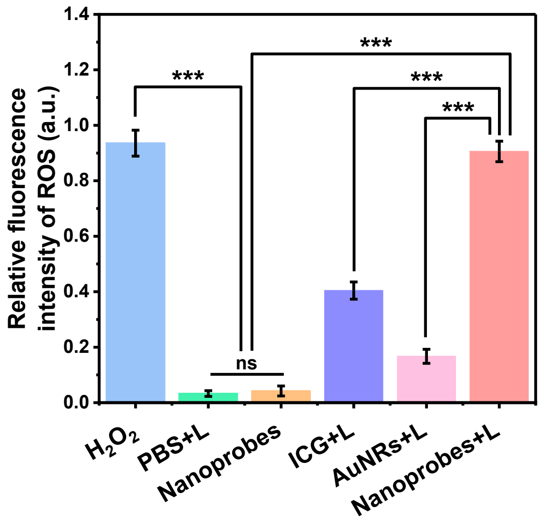

Figure S30. Quantitative analysis of the reactive oxygen species (ROS) detection assay under different treatments. L represents the 808 nm laser. Data are presented as mean ± SD, n = 3, *$p < 0.05$, **$p < 0.01$, ***$p < 0.001$; ns: not significant.

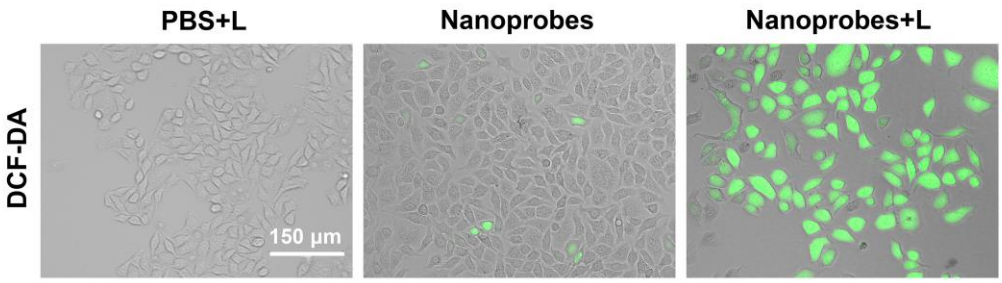

Fig. S31. Fluorescence images of CNE2 cells stained with DCFH-DA after treatments with PBS + L, nanoprobes and nanoprobes + L. L denotes 808 nm laser (0.5W cm^{-2}, 5 min).

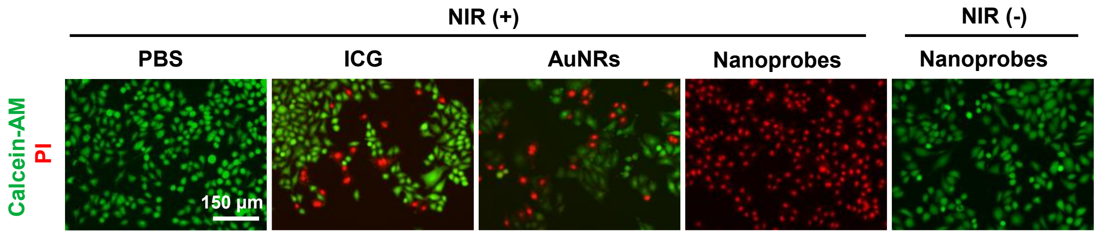

Fig. S32. Live/dead fluorescence images of 808 nm laser (0.5W cm^{-2}, 5 min) and nanoprobes treated CNE2 cells stained with calcein-AM (green) and PI (red).

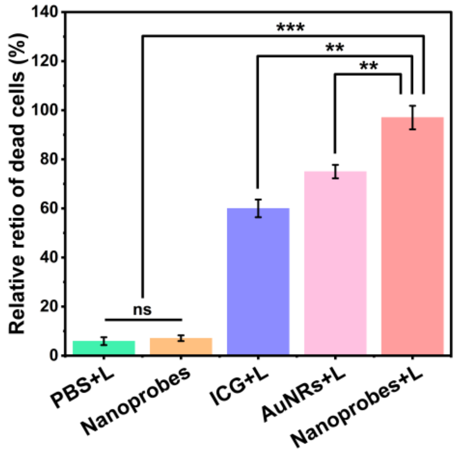

Figure S33. Quantitative analysis of cytotoxic effects induced by different treatments. The relative ratio of dead cells under each treatment was determined by live/dead cell staining, reflecting the cytotoxic effect of the corresponding treatment. Data are presented as mean ± SD, n = 3, *p < 0.05, **p < 0.01, ***p < 0.001; ns: not significant.

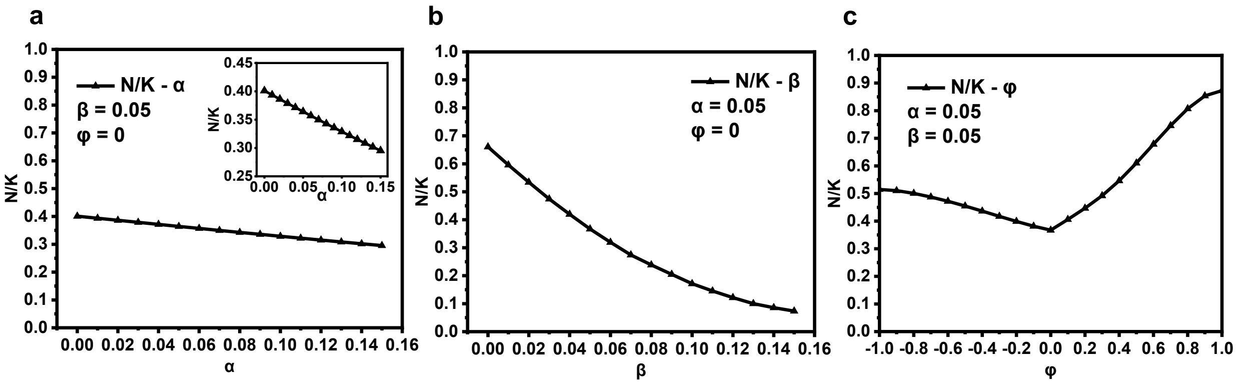

Fig. S34. Schematic diagram of a) the curve showing the effect of α on N/K, b) the curve showing the effect of β on N/K and c) the curve showing the effect of φ on N/K.

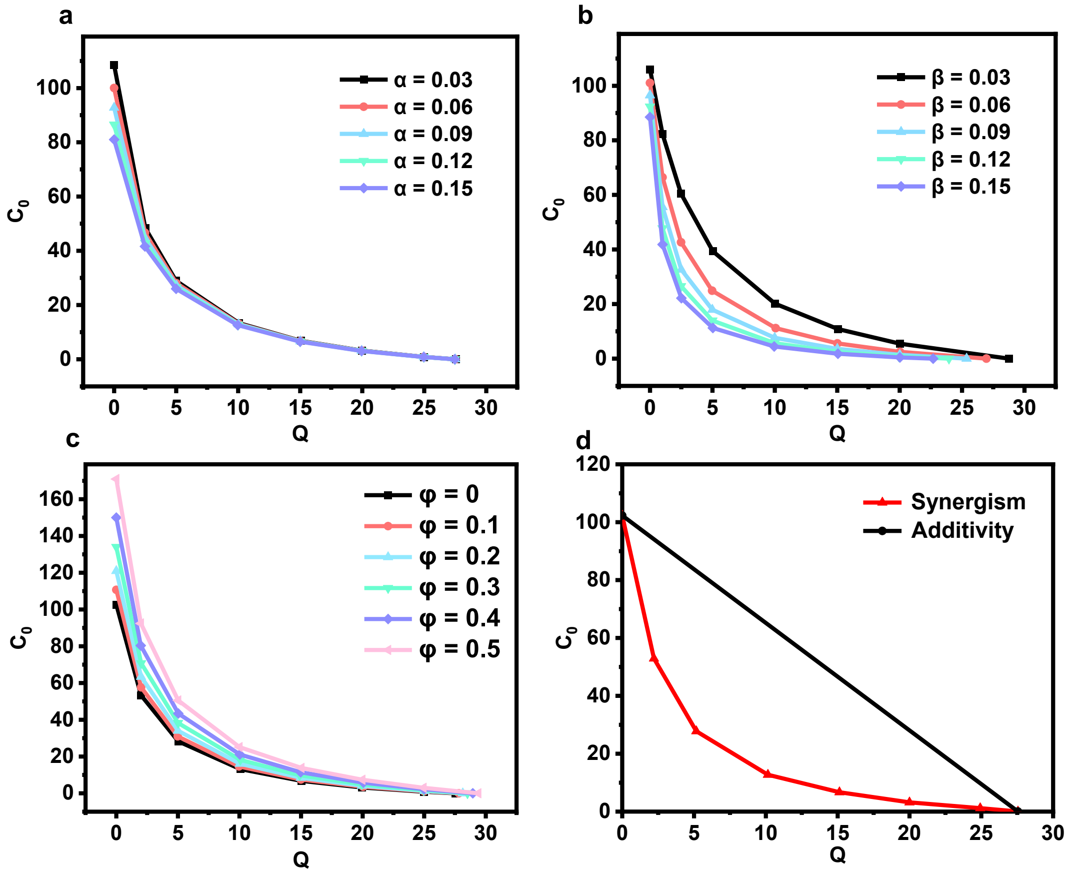

Fig. S35. Schematic diagram of a) the isoeffect curve corresponding to α values, b) the isoeffect curve corresponding to β values, c) the isoeffect curve corresponding to φ values and d) the isoeffect line of gold nanorod heat generation Q and initial photosensitizer concentration C_0 when the value of N/K is 0.5.

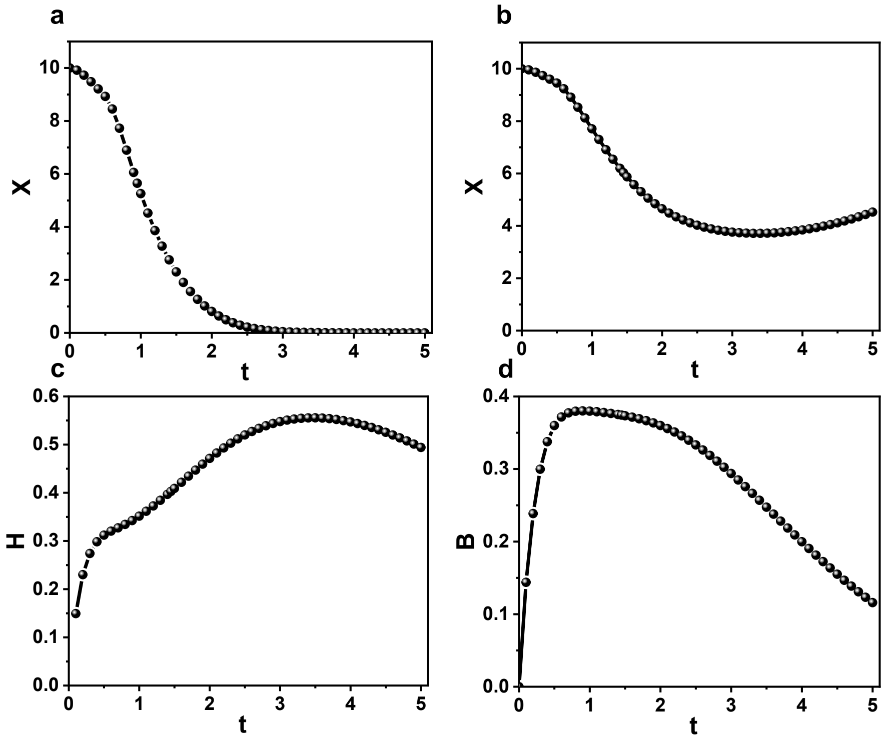

Fig. S36. Schematic diagram of the numerical solution image of the model for a) the change of x with time t when the time delay $\tau = 1$, $Q = 1.5$, and $C_0 = 10$, b) the change of x with time t when $\tau = 10$, $Q = 3$, and $C_0 = 20$, c) the change of H with time t when $\tau = 1$, $Q = 1.5$, and $C_0 = 10$ and d) the change of B with time t when $\tau = 1$, $Q = 1.5$, and $C_0 = 10$.

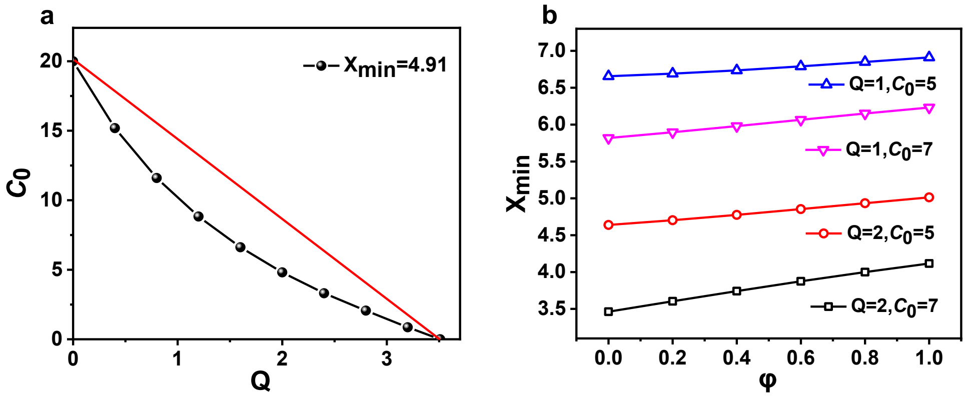

Fig. S37. Schematic diagram of a) the isoeffect curve when the value of x_{min} is 4.91 and b) the change of x_{min} value with φ under different Q and C_0.

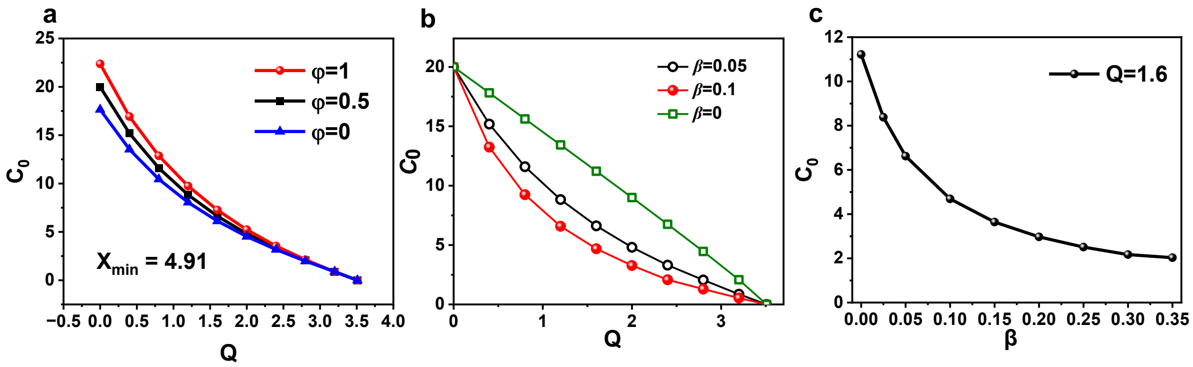

Fig. S38. Schematic diagram of a) the isoeffect curve corresponding to different φ values when x_{min} = 4.91, b) the isoeffect curve under different β values when x_{min} = 4.91 and c) the β-C_0 curve when x_{min} = 4.91 and Q = 1.6.

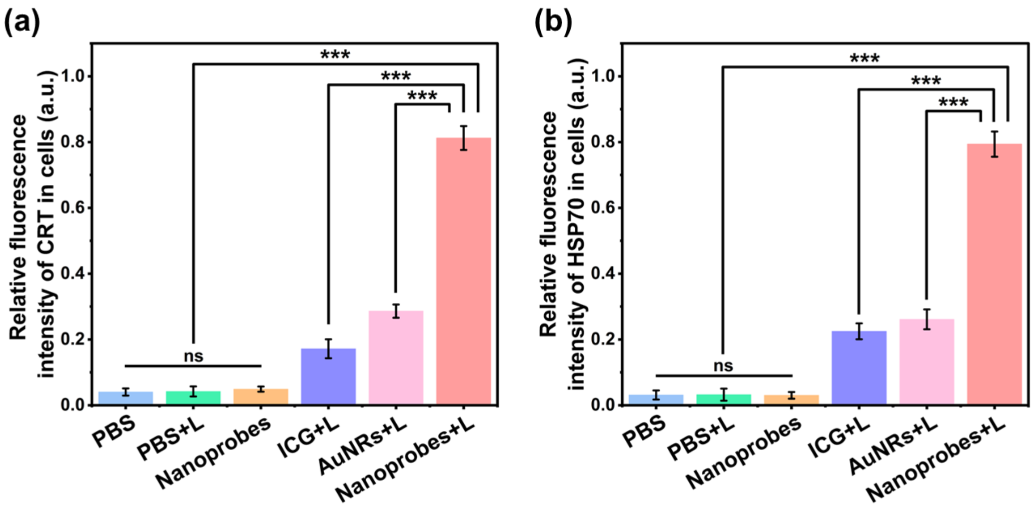

Figure S39. Quantitative analysis of immunogenic cell death (ICD). (a) Relative fluorescence intensity of CRT in cells under different treatments, indicating the level of calreticulin exposure, a key marker of ICD. (b) Relative fluorescence intensity of HSP70 in cells under different treatments, representing the expression of HSP70, a critical marker for the immunoadjuvant effect during ICD. Data are presented as mean ± SD, n = 3, *$p < 0.05$, **$p < 0.01$, ***$p < 0.001$; ns: not significant.

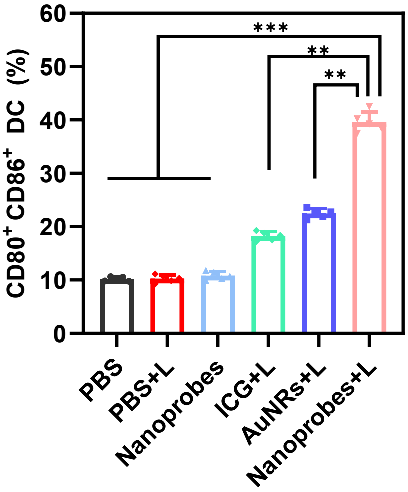

Figure S40. Quantitative analysis of DC maturation (stained with $CD80^+$ and $CD86^+$) detected by flow cytometry after different treatments. Data are presented as mean ± SD, *$p < 0.05$, **$p < 0.01$, ***$p < 0.001$.

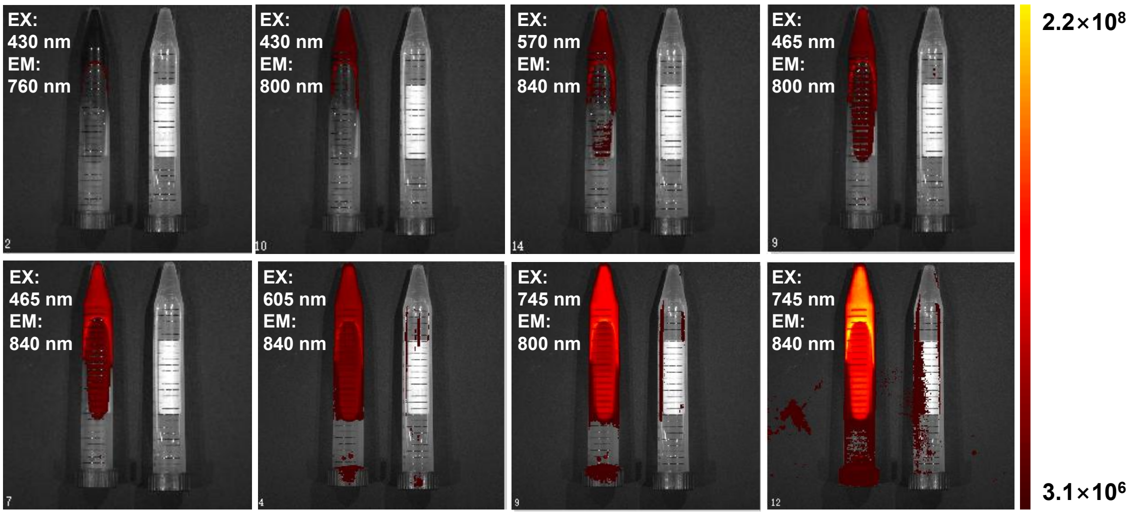

Fig. S41. NIR fluorescence imaging of the nanoprobe aqueous solution under varied laser excitations at emission wavelengths of 760 nm, 800 nm, and 840 nm compared to PBS group. The value of color bar indicates the max and min values of radiant efficiency.

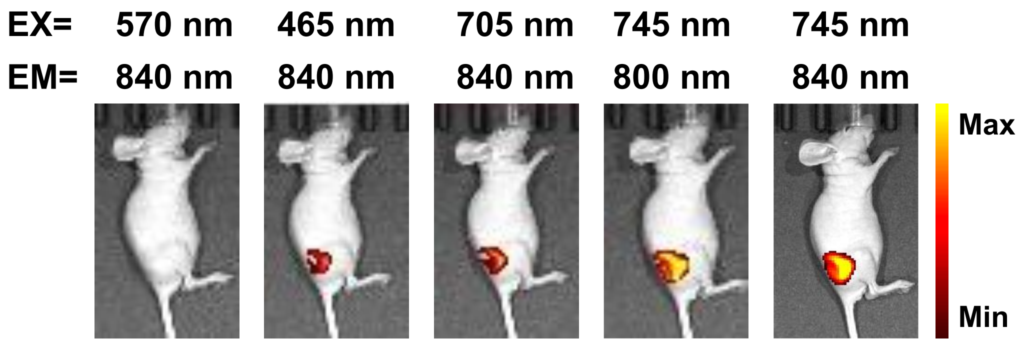

Fig. S42. NIR fluorescence imaging of tumor-bearing mice at 24 h after tail vein injection of the nanoprobe solution under varied laser excitations at emission wavelengths of 800 nm and 840 nm.

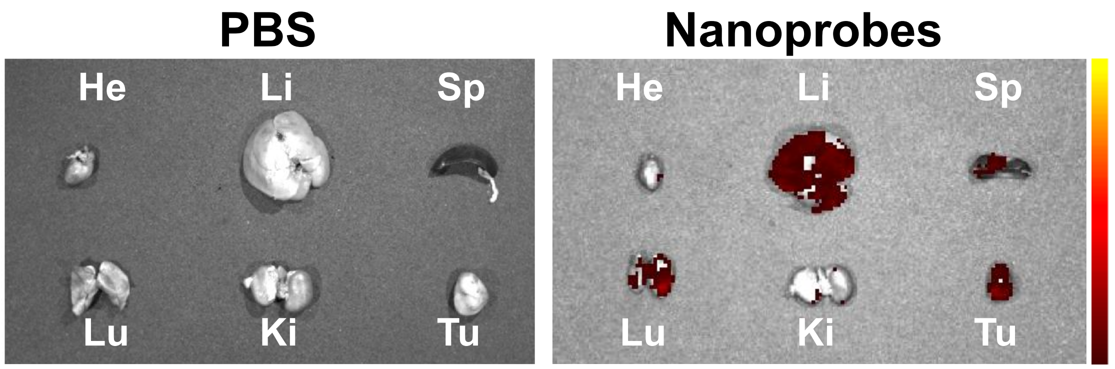

Fig. S43. Fluorescence images of major organs including heart, liver, spleen, lung, kidney and tumor at 7 days after tail vein injection with nanoprobes.

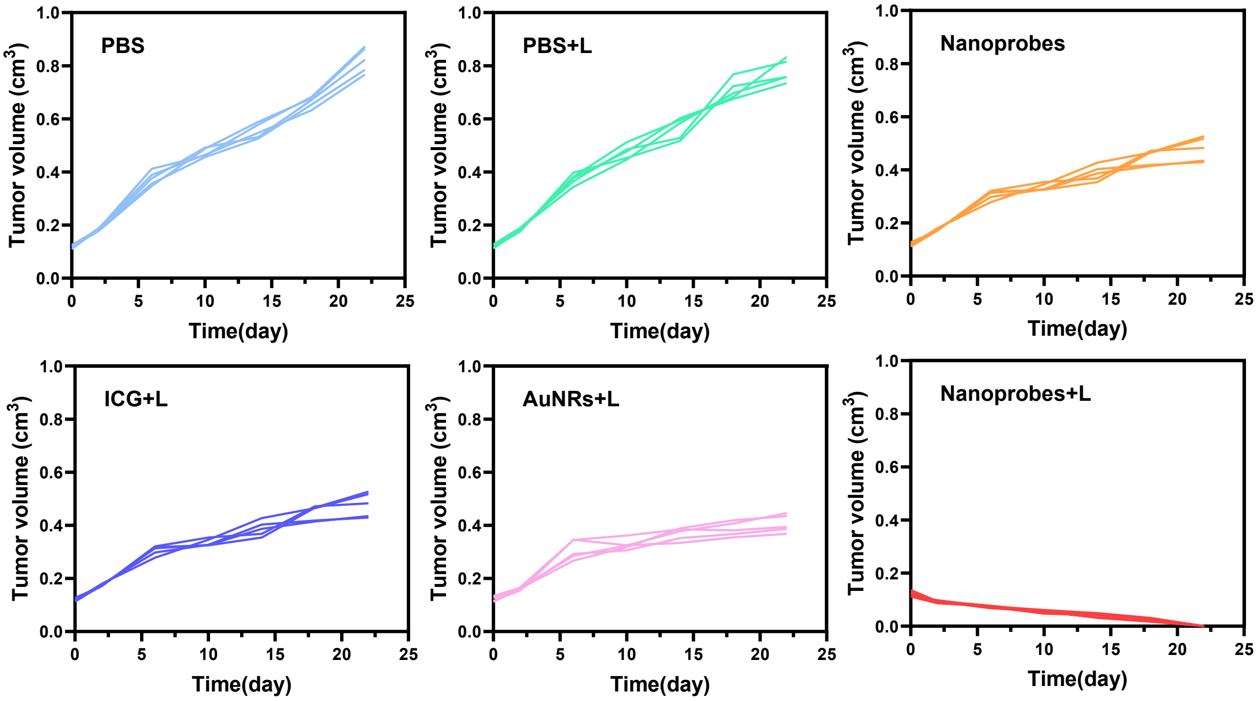

Fig. S44. Time-dependent tumor growth curves after different treatments (n = 5). L denotes 808 nm laser.

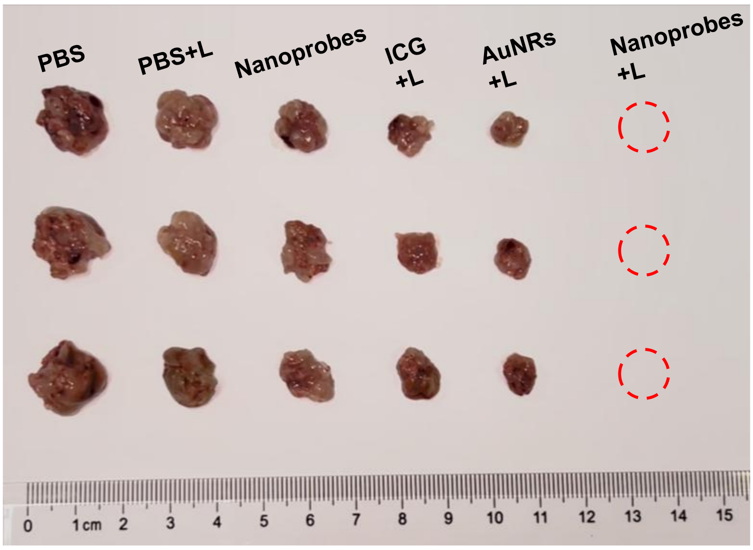

Fig. S45. Photographs of excised tumors from NPC-bearing mice 22 days after different treatments (n = 3). L denotes 808 nm laser.

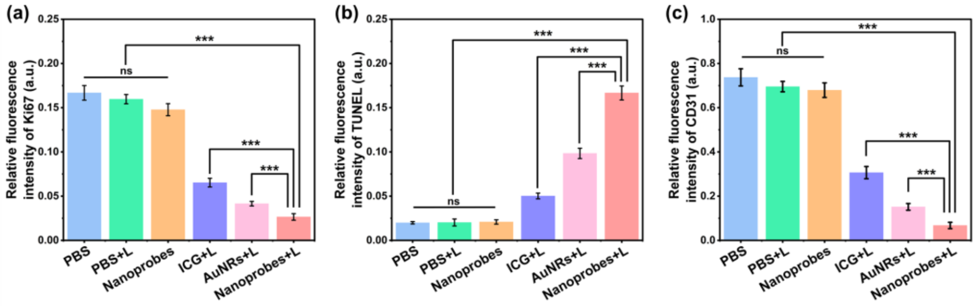

Figure S46. Quantitative analysis of cell proliferation, apoptosis, and angiogenesis under different treatments. (a) Relative fluorescence intensity of Ki67 in cells under different treatments, reflecting the level of cell proliferation. (b) Relative fluorescence intensity of TUNEL in cells under different treatments, indicating the level of cell apoptosis. (c) Relative fluorescence intensity of CD31 in cells under different treatments, representing the level of angiogenesis. L denotes 808 nm laser. Data are presented as mean ± SD, n = 5, *$p < 0.05$, **$p < 0.01$, ***$p < 0.001$; ns: not significant.

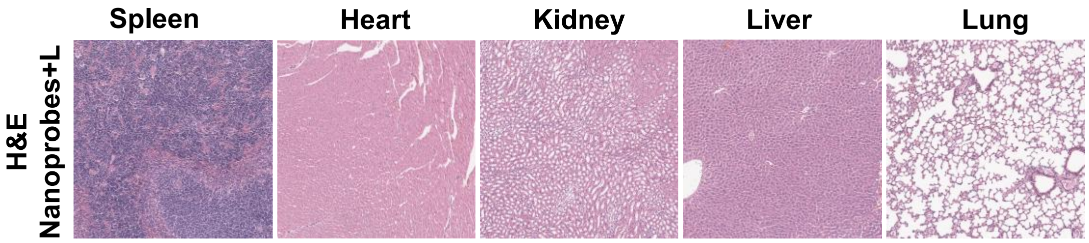

Fig. S47. HE staining images of major organs including heart, liver, spleen, lung and kidney from nanoprobes-treated mice after phototherapy (scale bar: 200 µm)

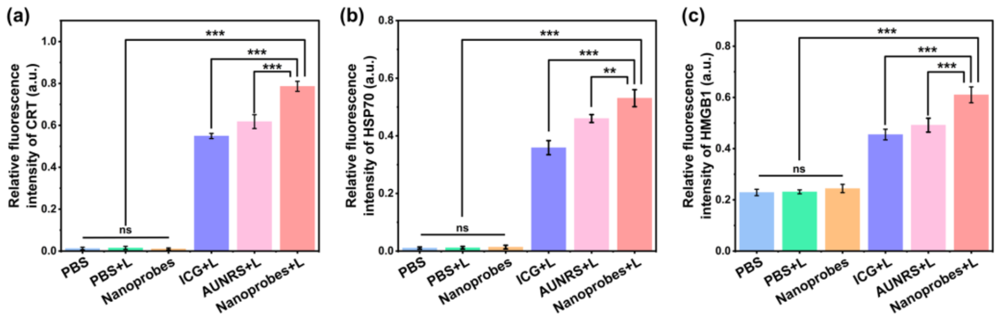

Figure S48. Quantitative analysis of key markers of immunogenic cell death (ICD) induced by different treatments. (a) Relative fluorescence intensity of CRT in cells under different treatments. (b) Relative fluorescence intensity of HSP70 in cells under different treatments. (c) Relative fluorescence intensity of HMGB1 in cells under different treatments. L denotes 808 nm laser. Data are presented as mean ± SD, n = 5, *p < 0.05, **p < 0.01, ***p < 0.001; ns: not significant.

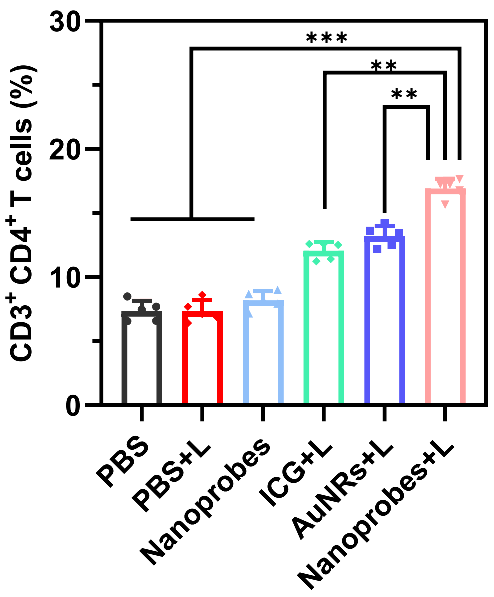

Fig. S49. The percentages of $CD3^{+}$ $CD4^{+}$ T cells in NPC-bearing mice with different treatments analyzed with flow cytometry. L denotes 808 nm laser. Data are presented as mean ± SD, n = 5, *p < 0.05, **p < 0.01, ***p < 0.001.

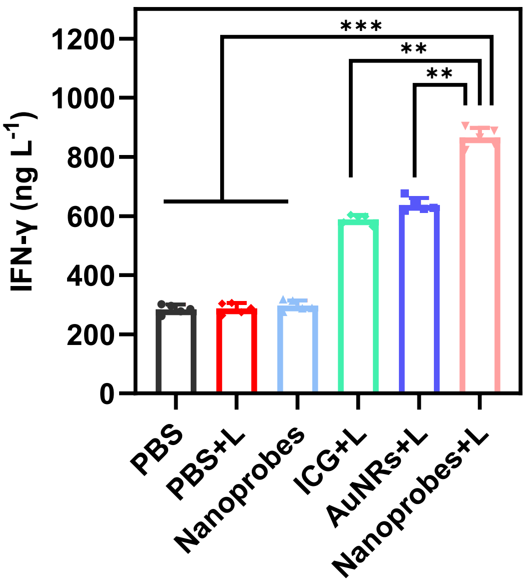

Fig. S50. Quantitative analysis of IFN-γ in serum after different treatments. L denotes 808 nm laser. Data are presented as mean ± SD, n = 5, *p < 0.05, **p < 0.01, ***p < 0.001.
